# Extraction of Extracellular Matrix in Static and Dynamic *Candida* Biofilms Using Cation Exchange Resin and Untargeted Analysis of Matrix Metabolites by Ultra-High-Performance Liquid Chromatography-Tandem Quadrupole Time-of-Flight Mass Spectrometry (UPLC-Q-TOF-MS)

**DOI:** 10.3389/fmicb.2019.00752

**Published:** 2019-04-10

**Authors:** Wenyue Da, Jing Shao, Qianqian Li, Gaoxiang Shi, Tianming Wang, Daqiang Wu, Changzhong Wang

**Affiliations:** Laboratory of Pathogenic Biology and Immunology, College of Integrated Chinese and Western Medicine (College of Life Science), Anhui University of Chinese Medicine, Hefei, China

**Keywords:** *Candida albicans*, cation exchange resin, extraction, biofilms, matrix, continuous flow

## Abstract

Fungal infections caused by *Candida albicans* poses a great threat to human health. The ability of biofilm formation is believed to be associated with resistance-related *Candida* infections. Currently, knowledge on extracellular matrix (EM) of *C. albicans* biofilm is limited. In this study, we introduced ion exchange resin, i.e., cation exchange resin (CER) and anion exchange resin (AER), in EM extraction of *C. albicans* biofilm as well as several non-*albicans Candida* (NAC) biofilms under static and dynamic states in combination with vortexing and ultrasonication (VU). The metabolites extracted from the dynamic *C. albicans* biofilm matrix using the CER-VU and VU were identified with ultra-high-performance liquid chromatography-tandem quadrupole time-of-flight mass spectrometry (UPLC-Q-TOF-MS) via untargeted filtration. Compared with other physical and chemical extraction methods, CER-VU was demonstrated to be an ideal approach with high-yield acquisitions of EM constituents including proteins, triglycerides and carbohydrates and low-level damages on fungal cell viability and integrity. The untargeted MS analysis further showed the high efficacy of CER-VU, as a large quantity of metabolites (217 versus 198) was matched comprising a great number of lipids, carbohydrates, amino acids, nucleic acids and their derivatives together with a high involvement of signaling pathways compared with the VU alone. However, combining the results from both the CER-VU and VU methods could generate more metabolites. In summary, the EM analysis of the dynamic *C. albicans* biofilm expands our understanding upon a comprehensive depiction of matrix components and provides another effective approach for EM extraction.

## Introduction

*Candida albicans*, a commonly isolated fungus normally colonizing the human intestinal tract, skin and vagina, can be transformed into an opportunistic pathogen which is able to cause anything from mild mucosal discomforts to deadly systematic infections when the host endures long-time antibiotic treatment or immunosuppressive difficulty (Gow et al., 2012). One of the most dangerous traits of *C. albicans* is its resistance to traditional antifungal agents, which is associated with the ability of biofilm formation, especially in patients with central venous catheters (CVCs)-related candidemia (Cuéllar-Cruz et al., 2012). The *C. albicans* biofilm is a heterogeneous, complex and three-dimensional architecture conferring protections to fungal cells encased by the secreted extracellular matrix (EM) from attacks of host immune system and antifungal agents (Tobudic et al., 2012). As for the first contact point with environmental cues, comprehensive information related to the delicate components of *C. albicans* biofilm EM is a primary task to search for potential antifungal targets (Bjarnsholt et al., 2013).

Growing demands are being raised for proper analytical technology and instrumentation to detect, quantify and characterize bacterial/fungal biofilms *in vitro* and *in vivo* for clinical and antimicrobial purposes (Vertes et al., 2012). To analyze intracellular metabolites in microorganisms, a group of extraction methods are developed, such as perchloric acid, hot water, boiling ethanol, chloroform–methanol, freezing–thawing cycles in methanol and acidic acetonitrile-methanol, etc. (Villas-Boas et al., 2005; Pinu et al., 2017). These methods aim to lyse target cell wall/membrane, release intracellular metabolic substances, and avoid metabolite degradations. By contrast, the biofilm EM is a congregate of extracellular metabolites, requiring an ideal extraction technique to maximize the secreted matrix (number and quantity) and minimize cell damage which is dependent on extraction procedures and operating conditions (Sun et al., 2012). The available studies on fungal biofilm EM extraction are scarce to date, and we summarized the literature concerning mainly the EM extraction of activated sludge and bacterial biofilms to the best of our knowledge (Supplementary Table S1). The extracted matrix components can be identified via untargeted or targeted filtration. As an advanced combination technique with high separation capacity and high efficiency in compound identification, ultra-high-performance liquid chromatography coupled to quadrupole time-of-flight mass spectrometry (UPLC-Q-TOF-MS) accelerates the elucidation of characteristic compound structures and accurate molecular weights compared to traditional HPLC-MS/MS (Zhao et al., 2014).

*In vitro* cultivation of *Candida* biofilms was usually performed under two states, i.e., static state and dynamic state. The static *C. albicans* biofilm formed on a 96-well flat-bottomed microplate is the most widely used model. The matrix constituents by proteomics and lipidomics as well as the matrix construction manner have been investigated in the static *Candida* biofilms (Lattif et al., 2008, 2011; Zarnowski et al., 2014). However, increasing evidence has revealed that the dynamic *C. albicans* biofilm had different phenotype and pathogenicity due to a denser and more complex structure as well as high resistance to conventional antifungal drugs compared with the static counterparts, and it was commonly employed to mimic *in vivo* biofilm development (Uppuluri et al., 2009; Uppuluri and Lopez-Ribot, 2010; Shao et al., 2015). The dynamic *C. albicans* biofilm EM possibly owns distinct constituents which might provide meaningful clues to the diverse responses of this fungal pathogen to external stresses and can broaden our views on *Candida* resistance and antifungal preventions.

This study aims to investigate the efficiencies of seven methods in *C. albicans* biofilm EM extraction, namely vortexing plus ultrasonication (VU), formaldehyde plus vortexing and ultrasonication (Formaldehyde-VU), EDTA plus vortexing and ultrasonication (EDTA-VU), NaOH plus vortexing and ultrasonication (NaOH-VU), ethanol plus vortexing and ultrasonication (Ethanol-VU), ion exchange resin plus vortexing and ultrasonication (IER-VU)—including cation/anion-exchange resin plus vortexing and ultrasonication (CER/AER-VU)—under the static and dynamic states. Subsequently, the extracted metabolites are analyzed with the UPLC-Q-TOF-MS tool via untargeted filtration.

## Materials and Methods

### Strains and Cultivation

*Candida albicans* SC5314 was kindly provided by Prof. Yuanying Jiang from the College of Pharmacy, the Second Military Medical University (Shanghai, China). All the stock cultures of these strains were conventionally preserved in Sabouraud agar and propagated in liquid Sabouraud medium (Hope Biotech, Co., Qingdao, China) at 37°C for 12–16 h when the cells reached the exponential phase. The revived *Candida* cells were collected at 5200 rpm (Leiboer Medical Devices, Beijing, China) and washed twice with sterile phosphate-buffered saline (PBS, Leagene, Beijing, China). The fungal cells were then resuspended in RPMI-1640 medium (Invitrogen, Carlsbad, CA, United States) and adjusted to a defined cell density using a hemocytometer prior to the following experiments.

### Biofilm Formation

The *Candida* biofilms were formed under two different states, i.e., the static state and the dynamic state as described in previous work but with fewer modifications (Shao et al., 2015). In brief, both the static and dynamic *Candida* biofilms were formed on a 6 cm × 0.5 cm (long × width) grooved polyvinyl chloride (PVC) catheter (Shuguang Jianshi, Luohe, China) which was pre-sterilized with 75% ethanol (v/v, Tianjin, China) overnight and rinsed several times by sterile H_2_O (Milli-Q Direct 8, Millipore, Billerica, MA, United States) before biofilm formation (Zarnowski et al., 2016). As for static biofilm formation, the revitalized *Candida* strains were resuspended in RPMI-1640 to a final concentration of 1 × 10^6^ CFU/mL. The aseptic PVC catheters were coincubated with the adjusted *Candida* strains in a rotator with 220 rpm at 37°C for 4 h to facilitate initial adherence. Then, the catheters were taken out by a sterile forceps, rinsed twice by sterile H_2_O, and immersed in 40 mL RPMI-1640 for another 20 h of incubation at 37°C. The dynamic biofilm formation possessed the same experimental processes as the static one prior to initial adherence. Following cleaning, the dynamic biofilm was formed on the PVC catheter surface in our modified gravity-supported free-flow biofilm incubator (GS-FFBI) (Shao et al., 2015) with continuous RPMI-1640 at a constant flow rate of 1 mL/min for another 20 h of incubation at 37°C.

### Extraction Procedures

The extraction methods were performed according to several previous studies with small modifications (Brown and Lester, 1980; Liu and Fang, 2002; D’Abzac et al., 2010; Sun et al., 2012; Zarnowski et al., 2014).

(a) Vortexing+Ultrasonication (VU): After 24 h of incubation, the static and dynamic *Candida* biofilms on the PVC catheters were removed mildly with a sterilized forceps into a test tube laden with 6 mL sterile H_2_O successively for 1 min of vortexing (Quick Mixer SK-1, Guowang Experimental Instrument Factory, Jintan, China) and 20 min of 50 kHz gentle sonication (DSA50-GL1, Desen Ultrasonic Equipment, Fuzhou, China) at an amplitude of 0.45–0.55 w/cm^2^ at room temperature. The biofilm sample was then centrifuged at 7350 rpm for 3 min to separate fungal cells and EM. The precipitates were discarded, while the matrix-containing supernatant was filtered with a 0.22 μm millipore membrane (Millipore, Billerica, MA, United States) for subsequent chemical analysis.
(b) Formaldehyde+vortexing+ultrasonication (Formaldehyde-VU): Formaldehyde (0.025%, v/v, Shanghai Pilot Chemical Corporation, Shanghai, China) was used for incubation along with the formed static and dynamic biofilms for 1 h at room temperature and then heated at 80°C for 10 min. The following procedures were the same as those described in **a**.
(c) NaOH+vortexing+ultrasonication (NaOH-VU): NaOH (0.04 M, Macklin, Shanghai, China) was incubated with the matured static and dynamic biofilms for 3 h at room temperature. The remaining procedures were the same as those described in **a**.
(d) Ethanol+vortexing+ultrasonication (Ethanol-VU): Ethanol (1%, v/v) was adopted for 1 h of incubation with the static and dynamic biofilms. The other procedures were the same as those described in **a**.
(e) EDTA+vortexing+ultrasonication (EDTA-VU): The static and dynamic biofilms were treated with EDTA (2%, Sinopharm, Binjing, China) for 3 h. The residual procedures were the same as those described in **a**.
(f) Cation/Anion-exchange resin+vortexing+ultrasonication (CER/AER-VU): A twofold volume of CER/AER (Macklin, Shanghai, China) was transferred to a clean syringe. The preformed static and dynamic biofilms were exposed to 6 mL of NaH_2_PO_4_-Na_2_HPO_4_ buffer solution (pH 5.8, 6.8, 7.8; Yongda, Tianjin, China) or Tris-HCl buffer solution (pH 7.6, 8.1, 8.6; Solarbio, Beijing, China) successively for 1 min of vortex and 20 min of 50 kHz gentle sonication at an amplitude of 0.45–0.55 w/cm^2^ at room temperature. The matrix-containing buffer solution was inhaled into the CER/AER-laden syringe and handled with the resins for 3 h at a rotator of 400 rpm. The treated buffer solution was successively centrifuged at 7350 rpm for 3 min and filtered with a 0.22 μm millipore membrane. The supernatant was removed for the following experiments, while the resins were eluted with 500 mM NaCl (Suyi, Shanghai, China) for 2 h at a rotation of 400 rpm. After 3 min of centrifugation at 7350 rpm and filtration with a 0.22 μm millipore membrane, the elution was preserved for further evaluations.

### Quantification of the EM Components

The quantitative analyses of the proteins, triglycerides and carbohydrates in the extracted *Candida* EM were performed by an enhanced BCA protein assay kit (Beyotime, Jiangsu, China), Triglyceride (TG) test kit (Bestbio, Shanghai, China), and Carbohydrate test kit (Solarbio, Beijing, China) according to the kit instructions.

### XTT Assay

The metabolic activity of the fungal cell membrane was routinely measured by XTT assay. Herein, the XTT assay was employed to monitor the effect of the extraction method on fungal cell viability and integrity. The XTT solution was freshly prepared by dissolving 50 mg XTT (Solarbio, Beijing, China) into 100 mL Riger’s solution which was then mixed with newly-made menadione solution (Dibo, Shanghai, China) being dissolved in acetone to a final concentration of 1.72 mg/mL at a proportion of 5000:1 (v/v) immediately before use. The prepared XTT solution was filtered with 0.22 μm millipore membrane. The broth containing the fungal cells (200 μL) was added into 50 μL of XTT solution in a 96-well microplate for 2 h at 37°C in the dark. The metabolic activity of the fungal cells was measured at a wavelength of 492 nm using a 318-microplate reader (Sanco Instruments, Shanghai, China).

### Colony-Forming Unit (CFU) Counting

After treatment with the extraction methods, the sample solution containing the *Candida* biofilm cells was spread onto YPD plates to quantify the fungal CFU with 10-fold serial dilution at 37°C following 24 h of incubation.

### Dry Weight Analysis

The dry weight of *Candida* biofilm EM was examined as described previously with less modifications (Shao et al., 2015). In brief, the dynamic biofilms were formed on the PVC catheters and then gently removed with tweezers into a sterile and empty Petri dish without disturbing the biofilm structures. Following respective extractions with the VU and CER-VU methods, the EM-containing solution was vacuum-dried at -80°C until the dish weight did not alter. The total dry weight of biofilm EM extracted was calculated by subtracting the pure weight of empty Petri dish from the weight of the one with dried EM.

### Identification of the EM Components

#### Sample Pretreatments

The EM samples were thawed at 4°C on ice. Then, 100 μL of the sample was transferred to an Eppendorf (EP) tube, extracted with 300 μL of methanol, mixed with 10 μL of internal standard (IS), vibrated for 30 s, treated with ultrasound for 10 min in ice water and incubated for 1 h at -20°C to precipitate proteins. Following by centrifugation at 12,000 rpm for 15 min at 4°C, 200 μL of supernatant was transferred to LC–MS vials and 20 μL from each sample (*n* = 6) was pooled as a QC sample. Another 200 μL of supernatant was analyzed by the UPLC-Q-TOF-MS. Both the samples treated with the CER-VU (SZ group) and VU (ST group) were prepared in sextuplicate.

#### UPLC-Q-TOF-MS Procedures

The separation was performed using a 1290 UPLC system (Agilent Technologies) equipped with a UPLC BEH Amide column (1.7 μm, 2.1 mm × 100 mm, Waters) coupled with a Q-TOF 6600 mass spectrometer (AB Sciex) with an electrospray ionization (ESI) source. The mobile phase consisted of 25 mM NH_4_OAc and 25 mM NH_4_OH (pH = 9.75, solvent A) and acetonitrile (solvent B). The gradient elution procedure was set as follows: 0–7 min, 95% B; 7–8 min, 65% B; 8–9.1 min, 40% B; and 9.1–12 min, 95% B. The flow rate was 0.5 mL/min. The IS was 2-chloro-L-phenylalanine. The ESI-Q-TOF-MS was applied on an information-dependent basis (IDA) in both positive and negative ion modes with the conditions as follows: ion source gas 1 pressure (GS1), 60 Psi; ion source gas 2 pressure (GS2), 60 Psi; curtain gas pressure, 35 Psi; source temperature, 650°C; ion spray voltage floating (ISVF), 5000 V (positive ion mode) or -4000 V (negative ion mode); mass range, m/z 60–1200.

#### Data Acquisition and Processing

The injection volumes for the positive and negative mode were 4 and 2 μL, respectively. During the operation, the acquisition software Analyst TF1.7 (AB Sciex) continuously evaluated the full scan survey MS data it collected and triggered the acquisition of MS/MS spectra depending on preselected criteria. In each cycle, 12 precursor ions whose intensity greater than 100 were chosen for fragmentation at a collision energy (CE) of 30 eV (15 MS/MS events with product ion accumulation time of 50 ms each). MS raw data were converted to the mzXML format using ProteoWizard^1^ and processed by self-programmed R package XCMS (version 3.2^2^). The preprocessing results generated a data matrix that consisted of the retention time (RT), mass-to-charge ratio (m/z) values, and peak intensity. The R package CAMERA was used for peak annotation after the XCMS data processing. An in-house MS2 database was applied in the metabolite identifications.

### Statistical Analysis

The extraction and quantification procedures were performed in triplicate. The results were reported as mean ± standard deviation, calculated by SPSS 17.0, and processed by one-way ANOVA with least significant difference (LSD) method. The comparison among groups was done by Student’s *t*-test. The statistical significance was defined as *p* < 0.05. The output data including the peak areas of all metabolites were normalized to the IS prior to multivariate data analyses, including principal component analysis (PCA) and orthogonal projections to latent structures-discriminant analysis (OPLS-DA), with SIMCA-P software (Version 14.1, Umeå, Sweden). The data for the multivariate analyses required log transformation to eliminate the unit error. The differentially expressed metabolites were identified according to a threshold of variable importance in the projection values above 1 which could be generated after the OPLS-DA processing combined with *p* < 0.05. The metabolites of interest were identified by matching the measured accurate mass and isotopic pattern with the database (HMDB/PubChem/ChEBI/KEGG/METLIN). We used the matched KEGG compound ID to identify differentially-expressed signaling pathways^3^.

## Results

### Extraction Yields of the Biofilm EM in *Candida* spp.

**FIGURE 1:**
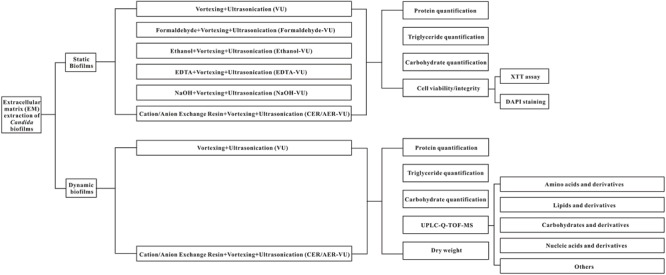
Schematic flowchart of the EM extraction metabolite profiling used in this study.

**FIGURE 2:**
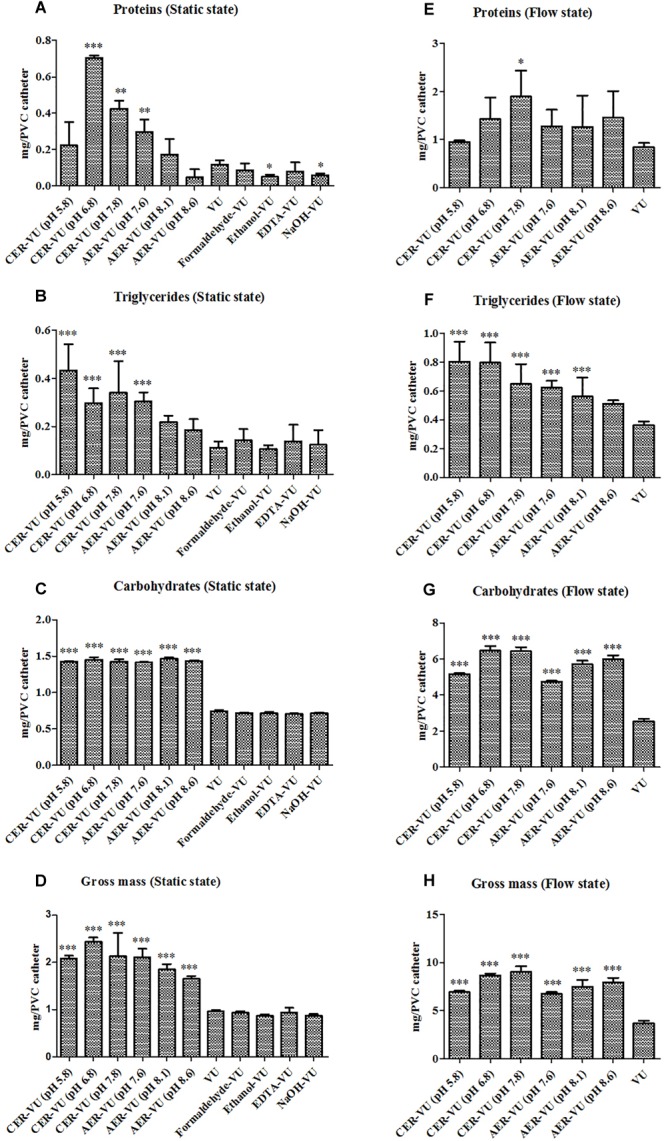
Chemical analyses of the **(A,E)** proteins, **(B,F)** triglycerides, and **(C,G)** carbohydrates as well as the calculation of the **(D,H)** gross mass in *Candida albicans* biofilm EM extraction using vortexing plus ultrasonication (VU), formaldehyde plus vortexing and ultrasonication (Formaldehyde-VU), EDTA plus vortexing and ultrasonication (EDTA-VU), NaOH plus vortexing and ultrasonication (NaOH-VU), ethanol plus vortexing and ultrasonication (Ethanol-VU), and cation/anion exchange resin plus vortexing and ultrasonication (CER/AER-VU) at pH 5.8, 6.8, 7.8 for the CER and pH 7.6, 8.1, 8.6 for the AER under static and dynamic states. ^∗^*p* < 0.05; ^∗∗^*p* < 0.01; ^∗∗∗^*p* < 0.001; compared with those extracted by the VU.

The experimental design is assembled and plotted in detail in Figure 1. In the static state, the CER-VU (pH = 6.8/7.8) and AER-VU (pH 7.6) acquired significantly higher concentrations of the proteins and triglycerides than the VU alone did, with 2.55–6.05 (*p* < 0.01 and *p* < 0.001)- and 2.68–3.08 (*p* < 0.001)-fold increases, respectively (Figure 2A,B), while in the six pH points tested (i.e., pH 5.8, 6.8, 7.8 and 7.6, 8.1, 8.6), both IERs extracted more carbohydrates than the VU alone, with a 1.90–1.98-fold increase (*p* < 0.001, Figure 2C). The extraction yields of the other four methods, i.e., Formaldehyde-VU, EDTA-VU, NaOH-VU and Ethanol-VU, were comparable to those of the VU but much less than those of the CER/AER-VU in the extractions of the proteins, triglycerides, and carbohydrates (Figure 2A–C). The gross mass of the three EM components with the CER/AER-VU method were also considerably higher than those with the VU alone, with a 1.72–2.54-fold increase (*p* < 0.001, Figure 2D). The study on the static biofilm extractions showed a desired extraction efficiency with the CER/AER-VU compared with the other methods including the VU.

Based on the results in the static condition, we further evaluated the extraction yields with the CER/AER-VU method in comparison to the VU via measuring the concentrations of the proteins, triglycerides, and carbohydrates extracted under the dynamic state. We observed that the protein concentration increased significantly by 2.26-fold using the CER-VU (pH 7.8) compared with that using the VU (*p* < 0.05, Figure 2E), while the extracted triglycerides and carbohydrates with the CER/AER-VU were remarkably abundant compared to those extracted with the VU, and the concentrations of both bio-macromolecules increased in the range of 1.55–2.22- and 1.87–2.56-fold, respectively (*p* < 0.001, Figure 2F,G). We further noted that the CER/AER-VU promoted significant ascending of gross mass, ranging 1.81–2.42-fold compared with the VU (*p* < 0.001, Figure 2H). The results on the dynamic biofilm extractions favored the extraction efficiency of the CER/AER-VU method. Thus, their effects on the viability and integrity of fungal cells were deserved for further evaluation. We further measured the dry weights of *Candida* biofilm EM and calculated the extraction recoveries with the VU and CER-VU at pH 5.8, 6.8 and 7.8. The proteins, triglycerides and carbohydrates had 81.07%, 90.49% and 90.30% of recoveries with the CER-VU at pH 5.8, 6.8 and 7.8 compared to 58.65% with the VU (Table 1).

**Table 1:** Dry weight measurement in the dynamic *Candida albicans* biofilms.

Extraction methods	Extracted components (mg/catheter)				Recovery (%)
	Proteins	Triglycerides	Carbohydrates	Gross dry weight	
VU	0.84	0.36	2.53	6.36	58.65
CER-VU (pH 5.8)	0.95	0.80	5.17	8.57	81.07
CER-VU (pH 6.8)	1.43	0.79	6.47	9.58	90.49
CER-VU (pH 7.8)	1.90	0.65	6.43	10.02	90.30

Using the CER-VU method at pH 5.8, 6.8 and 7.8, the biofilm EM extraction of three non-*albicans Candida* (NAC) species, i.e., *C. glabrata, C. krusei*, and *C. tropicalis*, which are the clinically major isolated NACs (Cheng et al., 2005), was performed under both the static and dynamic states. The results showed that, in most cases, the CER-VU method at pH 7.8 had the best performance with an increased yields of proteins, triglycerides and carbohydrates as well as gross mass compared with the VU (Supplementary Figures S1–S3). Combined with the results from *C. albicans* and NAC species, more metabolites could possibly be extracted with the CER-VU at pH 7.8, and further analysis of the differentially expressed metabolites by the untargeted screening became necessary.

### Stability of the Extraction Methods

**FIGURE 3:**
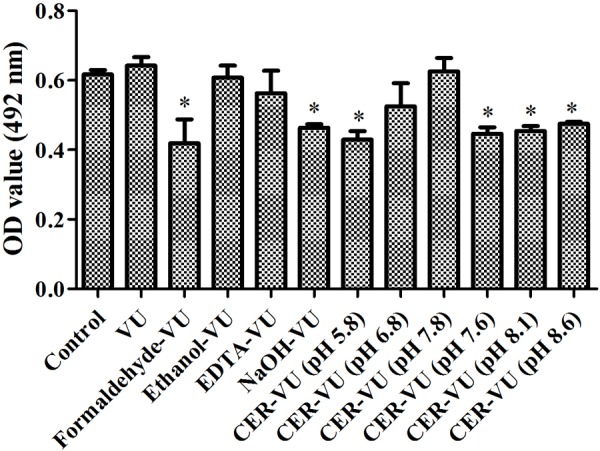
Metabolic activities by XTT assay in *C. albicans* biofilm EM extraction using vortexing plus ultrasonication (VU), formaldehyde plus vortexing and ultrasonication (Formaldehyde-VU), EDTA plus vortexing and ultrasonication (EDTA-VU), NaOH plus vortexing and ultrasonication (NaOH-VU), ethanol plus vortexing and ultrasonication (Ethanol-VU), and cation/anion exchange resin plus vortexing and ultrasonication (CER/AER-VU) at pH 5.8, 6.8, 7.8 for the CER and pH 7.6, 8.1, 8.6 for the AER under static state. ^∗^*p* < 0.05; compared with the control free of any extraction treatment.

**FIGURE 4:**
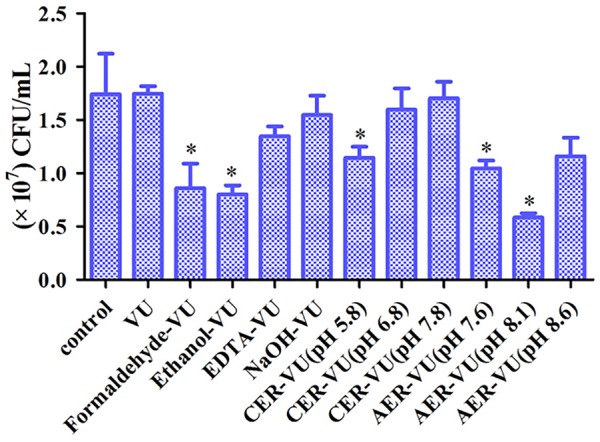
Colony-forming unit (CFU) counting of the biofilm cells after treatment with the extraction methods in *C. albicans* SC5314. The experimental conditions consisted of control, vortexing plus ultrasonication (VU), ethanol plus vortexing and ultrasonication (Ethanol+VU), formaldehyde plus vortexing and ultrasonication (Formaldehyde-VU), NaOH plus vortexing and ultrasonication (NaOH-VU), EDTA plus vortexing and ultrasonication (EDTA-VU), cation/anion exchange resin plus vortexing and ultrasonication (CER/AER-VU) at pH 5.8, pH 6.8, pH 7.8, pH 7.6, pH 8.1, and pH 8.6. ^∗^*p* < 0.05; compared with the control free of any extraction treatment.

XTT assay monitors the metabolic activity of the cell membrane and reflects fungal cell viability. CFU counting is a widely used approach to evaluate the survival of fungal isolates. As shown, the CER-VU at 7.8 had a negligible effect on the viability of *C. albicans* SC5314 (Figure 3, 4).

### Differentially Expressed Metabolites by the VU-CER and VU

We further used the CER-VU method to extract the dynamic biofilm EM and analyzed the matched metabolites by untargeted screening of the database. An entire quantity of 3219 ion peaks was generated and 1217 were retained by aligning the mass spectrometric data using XCMS upon both the negative and positive ion modes. The total ion flows of the VU and CER-VU in the negative and positive ion modes are presented in the supplementary information (Supplementary Figures S4–S7). The PCA, an unsupervised method, was applied to explore correlations between the CER-VU and VU groups. Good separations of the two groups were shown in the negative (R^2^X = 0.922) and positive (R^2^X = 0.929) ion modes (Supplementary Figures S8A, S9A). To confirm the discrimination of ion peaks between the two groups, the OPLS-DA was employed. Compared with the results of PCA, a similar separation tendency was also observed in the negative (R^2^Y = 0.991, Q^2^= 0.968) and positive (R^2^Y = 0.998, Q^2^= 0.974) ion modes (Supplementary Figures S8B, S9B). The permutation test (200 times) for model validation was also performed with intercepts generated in the negative (R^2^Y = 0.54, Q^2^= -1.23) and positive (R^2^Y = 0.7, Q^2^= -1.09) ion modes (Supplementary Figures S8C, S9C), indicating that the prediction ability and reliability of this model were quite satisfactory. The model validity is generally defined as if all the permuted R^2^ and Q^2^ values to the left are no higher than the original point to the right, and the intercept of the Q^2^ should be negative (Chen et al., 2014). To compare the efficiencies of the two extraction methods (i.e., CER-VU and VU), the variable influence in projection (VIP > 1) which revealed the significance of a metabolite to the model and *p* < 0.05 were adopted to screen the candidates that could be considered differentially expressed in the CER-VU or VU group. Based on this algorithm, 63 metabolites were selected, and their relative expression changes and categories were presented accordingly. It was shown that 22 amino acids and derivatives (14 upregulations vs. 8 downregulations), 9 lipids and derivatives (6 upregulations vs. 3 downregulations), 5 carbohydrates and derivatives (4 upregulations vs. 1 downregulation), 8 nucleic acids and derivatives (6 upregulations vs. 2 downregulations) as well as 19 other metabolites (11 upregulations vs. 8 downregulations) were identified to be differentially expressed (Supplementary Table S3). The expression changes of the selected metabolites verified that the extraction efficacy of the CER-VU was relatively superior to that of the VU.

### Untargeted Matched Metabolites by UPLC-Q-TOF-MS

**FIGURE 5:**
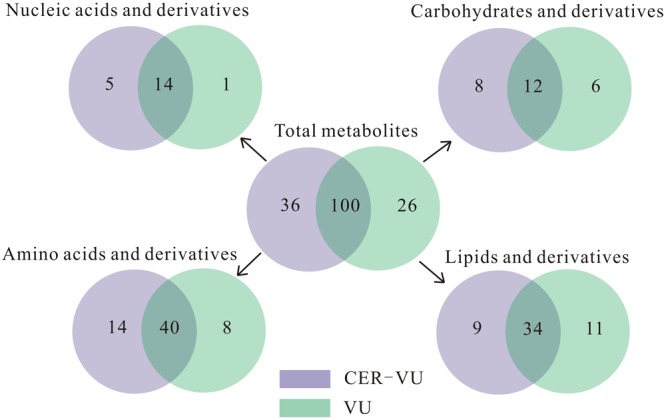
Metabolite numbers of *C. albicans* biofilm EM via untargeted filtration using cation exchange resin (CER) plus vortexing and ultrasonication (CER-VU, light purple) at pH 7.8 and vortexing plus ultrasonication (VU, light green) under continuous flow state. The metabolites were classified into four groups: lipids and derivatives, carbohydrates and derivatives, amino acids and derivatives, and nucleic acids and derivatives.

Upon the untargeted filtration of the ion peaks and removal of the same metabolites, 218 and 199 metabolites with 154 in common were matched according to the data from the UPLC-MS with the CER-VU and VU. We also categorized these metabolites into four classes, namely the lipids and derivatives, carbohydrates and derivatives, amino acids and derivatives, as well as nucleic acids and derivatives, which amounted to 136, with the CER-VU including 36 different metabolites, and 126, with the VU including 26 disparate ones (Figure 5).

#### Lipids and Derivatives

Of the extracted lipids and derivatives, 43 and 45 were respectively identified with the CER-VU and VU methods among which 34 were identical (Figure 5). The lipid profile constituted a very small portion of sphingolipids (<0.05%) and polar glycerolipids (<0.4%) in both methods. In polar glycerolipids, phosphatidylcholine and phosphatidylethanolamine were found, but phosphatidylserine and phosphatidylinositol were not tracked directly despite the presence of glycerol, serine, and myo-inositol. A vast proportion of fatty acids was detected consisting of 12:0, 14:0, 15:0, 16:0, 16:1, 17:0, 18:0, 18:1, 18:2, 20:3, 20:4, 22:0, 24:0, and 26:0 (total carbons : total carbon-carbon double bonds) with the CER-VU method and 12:0, 12:3, 12:5, 14:0, 14:1, 15:0, 16:0, 16:1, 17:0, 18:0, 18:1, 18:2, 20:4, 22:0, 22:3, 22:6, 24:0, and 26:0 with the VU method. The most abundant fatty acid was stearic acid (79.55%/49.53%) followed by palmitic acid (12.14%/16.43%) and 20-HETE (22.25% extracted by the VU only), whereas dodecanoic acid (12:0), *cis-*9-palmitoleic acid (16:1), heptadecanoic acid (17:0), and hexacosanoic acid (26:0) occupied smaller portions using both methods (Supplementary Table S2). The lipids and derivatives identified with the CER-VU were implicated into four differentially regulated pathways, namely arachidonic acid metabolism, fatty acid elongation and metabolism, and alpha-linolenic acid metabolism, whereas those extracted with the VU were related to two pathways, i.e., inositol phosphate metabolism and the phosphatidylinositol signaling system (Table 2).

**Table 2:** Differential expressed pathways yusing the CER-VU and VU extraction methods.

CER-VU	VU
cal00230 Purine metabolism^a^	cal00400 Phenylalanine, tyrosine, and tryptophan biosynthesis^b^
cal00260 Glycine, serine, and threonine metabolism^b^	cal00350 Tyrosine metabolism^b^
cal00240 Pyrimidine metabolism^a^	cal00750 Vitamin B6 metabolism
cal00500 Starch and sucrose metabolism^c^	cal00480 Glutathione metabolism^b^
cal00220 Arginine biosynthesis^b^	cal00650 Butanoate metabolism
cal00040 Pentose and glucuronate interconversions^c^	cal00460 Cyanoamino acid metabolism^b^
cal00310 Lysine degradation^b^	cal00051 Fructose and mannose metabolism^c^
cal00332 Carbapenem biosynthesis	cal00053 Ascorbate and aldarate metabolism
cal00300 Lysine biosynthesis^b^	cal00130 Ubiquinone and other terpenoid-quinone biosynthesis
cal00590 Arachidonic acid metabolism^d^	cal00620 Pyruvate metabolism
cal00190 Oxidative phosphorylation cal00010 Glycolysis/gluconeogenesis^c^	cal00640 Propanoate metabolism cal00562 Inositol phosphate metabolism^d^
cal00062 Fatty acid elongation^d^	cal04070 Phosphatidylinositol signaling system^d^
cal00472 D-Arginine and D-ornithine metabolism^b^	
cal01212 Fatty acid metabolism^d^	
cal00592 alpha-Linolenic acid metabolism^d^	

*The superscripts a–d represents nucleic acids and derivatives-, amino acids and derivatives-, carbohydrates and derivatives-, lipids and derivatives-related pathways respectively.*

#### Carbohydrates and Derivatives

We identified 20 carbohydrates and derivatives with the CER-VU method and 18 with the VU method, among which 12 were in common (Figure 5). In *C. albicans*, the carbohydrates of concern are mannan, chitin and glucan, which are the main constituents of the cell wall and are believed to be linked with host immune recognition and response (Brown and Gordon, 2003; Keppler-Ross et al., 2010; McKenzie et al., 2010; Mora-Montes et al., 2011). In this study, the mannan, chitin and glucan were absent in the metabolite list, but their construction units, i.e., mannose, *N*-acetyl-glucosamine and D-glucose-1-phosphate, were defined using the CER-VU/VU method (Supplementary Table S2). We also found that the most abundant carbohydrate and derivative was 2-keto-D-gluconic acid/ribitol (18.03/45.30%) followed by D-erythrose 4-phosphate/xylitol (17.96/10.02%) and alpha-D-glucose 1-phosphate/*N*-acetyl-D-galactosamine (17.96/9.59%) using the CER-VU/VU. The KEGG analysis showed there were three remarkably upregulated pathways including starch and sucrose metabolism, pentose and glucuronate interconversions and glycolysis/gluconeogenesis with the CER-VU method, while only one pathway, i.e., fructose and mannose metabolism, was downregulated (Table 2).

#### Amino Acids and Derivatives

Prior to metabolite analyses, most proteins were removed from the extraction solution. Thus, the amino acids and derivatives matched might not be the protein degradations in the EM. In this study, there were 55 and 49 amino acids and derivatives identified using the CER-VU and VU methods, respectively (Figure 5). Among the 20 commonly-found amino acids, which are also the brick subset of proteins, glycine, cysteine, histidine, lysine, and valine were not present in monomeric state but in dipeptide form by the both extraction methods, while leucine and isoleucine could only be extracted with the VU or CER-VU (Supplementary Table S2). The upregulated pathways associated with amino acids and derivatives consisted of five, including glycine, serine, and threonine metabolism; arginine biosynthesis; lysine degradation; lysine biosynthesis; D-Arginine and D-ornithine metabolism vs. four containing phenylalanine, tyrosine and tryptophan biosynthesis; tyrosine metabolism; glutathione metabolism; and cyanoamino acid metabolism in the CER-VU vs. VU (Table 2).

#### Nucleic Acids and Derivatives

In the mature *C. albicans* biofilm matrix, nucleic acids, especially extracellular DNA, were demonstrated to be an essential element conferring a heterogeneity of biofilm formation and a great contribution to biofilm structural integrity and maintenance (Martins et al., 2009; Rajendran et al., 2014). As shown, 19 and 15 of nucleic acids and derivatives were determined, with 14 shared in both the CER-VU and VU groups (Figure 5). The upregulated pathways relevant to nucleic acids and derivatives included purine metabolism; pyrimidine metabolism vs. zero in the CER-VU vs. VU (Table 2).

## Discussion

### The CER-VU Preferentially Produced High Yields of *Candida* Biofilm EM

As indicated, the extraction methods and experimental conditions determined the extraction efficiency to a large extent which, at least, should take two aspects into consideration, namely the extraction yield and cell integrity (Sun et al., 2012). Heating promotes EM production and has already been adopted for EM extraction. Meanwhile, however, this approach may destroy microbial cell structure due to its high temperature (Tapia et al., 2009), consistent with our results (Figure 3, 4). Sonication is a common physical extraction method and supposed to promote the EM extraction by breaking bonds, as electrostatic and hydrophobic bonds are prevalent in the EM of varying sources (Dignac et al., 1998). We found that sonication was a critical step for *Candida* biofilm EM extraction, as this step can yield more proteins, triglycerides, and carbohydrates (data not shown). EDTA may form complexes with the EM which can be removed with difficulty and cause microbial cell lysis, the release of intracellular DNA and plenty of unidentified constituents (Liu and Fang, 2002). Formaldehyde has been verified to react with amino groups of proteins or aminopolysaccharides, generating the alkylation of cell wall molecules or EM and increasing the EM extraction yield in activated sludges (Comte et al., 2006). It should be noted that the residual formaldehyde can react with amino groups of the molecules present in the EM solution and then alter the properties of these molecules (D’Abzac et al., 2010). NaOH increases pH, dissociates acidic groups in the EM and generates repulsion among negatively charged EM, which promotes the EM solubility for extraction (Liu and Fang, 2002). Ethanol is a frequently-used method mainly for polysaccharide extraction (Ratto et al., 2006). Except for the VU, the other four methods including the formaldehyde-VU, EDTA-VU, NaOH-VU, and ethanol-VU were all chemical extraction techniques. Although they have satisfactory performance in the EM extractions of activated sludges and bacterial biofilms which might be possibly associated with extraction conditions and extracted samples (Supplementary Table S1), the chemical extractions are more inclined to contaminate the EM samples and cause dramatically high recoveries (>100%), which may be due to the cell lysis and unsuitable modifications of the cell wall molecules (D’Abzac et al., 2010). The CER enacts two types of different mechanisms: the application of shear force by stirring (physical extraction) and the removal of bivalent cations such as Ca^2+^ (chemical extraction) (Frølund et al., 1996). The combination of the two extraction mechanisms of CER facilitates disorganization of the EM structure and extraction of the EM components, and minimizes the risk for microbial cell lysis. It has been reported that a mild extraction method with minimum risk of cell lysis demanded a short extraction time (0.5–1 h) and relatively low stirring intensity (≤600 rpm), whereas an effective extraction condition with comparatively high but acceptable cell lysis needed a longer extraction time (≥12 h) and relatively high stirring intensity (≤900 rpm) (Frølund et al., 1996). Herein, we used a shaker with a lower rotation speed (400 rpm) than that in activated sludge extractions and employed a scheme of relatively short extraction time (5 h) to avoid fungal cell damage. Based on these results, the CER-VU at pH 7.8 was proved to be a reliable and efficient extraction technique with high yield and less cell injury.

The proteins, triglycerides and carbohydrates have been described to account for approximately 55, 15, and 25% of the dry weight of *C. albicans* biofilm matrix, respectively (Pierce et al., 2017). In this study, however, the carbohydrates were found to take the greatest proportion in the three components under both the static and dynamic biofilms followed by the proteins and triglycerides (Figure 2). Uppuluri et al. (2009) showed that the dynamic *Candida* biofilms appeared to be very sticky and compact, implying that the dynamic biofilm was composed of massive carbohydrates with striking adherence, which was also confirmed by other reports (Al-Fattani and Douglas, 2006; Shao et al., 2015). During *Candida* biofilm development, adherence is a pivotal step for biofilm maturation in the early phase (Tobudic et al., 2012). Ghannoum and colleagues demonstrated *C. albicans*- and *C. tropicalis*-derived lipids had a significant block on adherence of the respective yeast cells to the buccal epithelial cells, but the same case was not observed in the weakly-adherent *Candida pseudotropicalis*. They also noticed that individual phospholipids, sterols and steryl esters were the main inhibitors of adherence, while the effects of triacylglycerols and free fatty acids could be neglected (Ghannoum et al., 1986). According to a documented study pertaining to lipid compositions, the percentage of phospholipids reached approximately 10.2% in the *C. albicans* biofilm matrix (Zarnowski et al., 2014). By comparison, the phospholipids in this study decreased to 0.14% with the CER-VU and 0.15% with the VU (Supplementary Table S2), inferring that the dynamic biofilm cells promoted adherence via decreasing the phospholipid proportion.

To our knowledge, the CER has not been applied in fungal biofilm EM extraction. Although, in this experiment, the extraction conditions (such as buffer types, pH, extraction time) were not optimized, we demonstrated that the significantly high-yield proteins, triglycerides, and carbohydrates were achieved with the CER-VU compared with the other physical or chemical methods used. We noticed that the CER was usually not a method of the first choice compared with several chemical extractions alone or in combination with, for example, NaOH and/or formaldehyde, in activated sludge EM extractions (Supplementary Table S1). Nevertheless, the CER-VU is still a promising alternative method for fungal biofilm EM extraction considering its efficacy and mild effects on cell viability and integrity (Figure 3, 4).

### The CER-VU Generated a High Variety of Metabolites

Lipids are a group of important constituents in the *Candida* biofilm matrix. The alterations in the expression of genes and proteins responsible for lipid composition have been demonstrated to be linked with *Candida* biofilm formation and azole resistance (Mukherjee et al., 2003; Yeater et al., 2007; Lattif et al., 2008). As defined, decreased sterol levels and unsaturation index as well as increased fatty acid levels along with shortened chain length in biofilms (incubated for ≥ 12 h) contributed to fungal biofilm maturation and drug resistance (Lomb et al., 1975; Pesti et al., 1985; Mukherjee et al., 2003). Ergosterol is a key component in the cell membrane of *C. albicans* and can be detected in the *Candida* biofilm matrix (Zarnowski et al., 2014). Herein, however, we could not trace any type of sterols with the UPLC-MS. We calculated the proportions of unsaturated fatty acids and those with short chain length (≤18 carbon) from the data of a published paper which mainly surveyed the EM in static biofilm (Zarnowski et al., 2014). It was found that the unsaturated fatty acids extracted with the CER-VU/VU declined to 1.79%/30.23% compared with 72.92%, and the percentage of fatty acids with short chain lengths with the CER-VU method were comparable to those in the static biofilm. We further observed that the phosphatidylcholine:phosphatidylethanolamine (PC:PE) ratio rose to 8.54/11.61 using the CER-VU/VU method vs. 2.34 reported by Lattif et al. (2011). Carbohydrates as a key constituent are widely distributed in bacterial and fungal biofilm EM, with diverse molecular structures and functions (Pierce et al., 2017). Although the intricate structure of mannan-glucan complexes (MGCxs) denotes a strong implication of community behavior in *Candida* biofilms (Zarnowski et al., 2014), it is unlikely to be evaluated by UPLC-MS. Amino acids have been witnessed to take an extensive part in signaling transduction, gene regulation, syntheses, and degradations of low-molecular weight nitrogenous substances with enormously biological interest (Wu, 2009). Consistent with the results by Chen et al. (2014), hydroxyproline, citrulline, and ornithine were matched with both the CER-VU and VU. However, it was odd that there were no clues of aspartic acid and tryptophan in the extracted EM of the dynamic biofilm with either method.

In comparison to the VU method, a larger number of metabolites were extracted and involved in signaling pathways using the CER-VU, suggesting the CER-VU could be a promising extraction method with better performance than the VU in the extraction of *C. albicans* biofilm EM. By analyzing the results of this study and other works associated with the *Candida* mature biofilm matrix, a list of expected consistencies as well as unexpected inconsistencies were present in qualitative descriptions and quantitative determinations of metabolites. Several factors deserve consideration. Firstly, the biofilm was formed under the dynamic state in this study but under the static state in others (Lattif et al., 2008; Chen et al., 2014; Zarnowski et al., 2014). A growing number of reports revealed the enormous discrepancies between the dynamic and static biofilms in the three-dimensional architecture, antifungal resistance and especially included constituents (Al-Fattani and Douglas, 2006; Uppuluri et al., 2009; Shao et al., 2015). Secondly, the fungal strain adopted in this work was the commonly used standard reference *C. albicans* SC5314, while clinical strains were employed in other research (Al-Fattani and Douglas, 2006; Zarnowski et al., 2014). It has been known that there were significant differences of cell wall compositions between azoles-resistant and -susceptible *Candida* strains (Vitali et al., 2017). Taff et al. (2012) declared that certain components (such as glucan) of the biofilm matrix were transported across the cell wall, inferring that the cell wall might be a key site for synthesizing and sorting several crucial components of the biofilm matrix. Thirdly, different extraction methods might have variable biological interpretations of metabolic data (Duportet et al., 2012). Carbohydrates, fatty acids, nucleic acids, and most proteins (pI < 7.0) usually take negative charges under physiological conditions. In that case, the AER is a preferable choice to the CER-VU for the EM extraction. Of interest, a higher yield of extraction with the CER-VU was realized in this study. Nevertheless, it cannot be excluded that the extracts with the AER-VU might identify more metabolites than those with the CER-VU due to the fact that a specific extraction method could be suitable for certain functional classes. Thus, the experimental conditions including sample preservation and pre-column derivatization, instruments for detection as well as detection mode (targeted or untargeted) should be optimized in the following investigations. Fourthly, the involvement of the quenching process is another crucial factor for extract efficiency. Increasing evidence displayed that quenching can avoid extracted intracellular metabolite degradations (Mashego et al., 2007; Winder et al., 2008; Canelas et al., 2009; Schneider et al., 2009; Kim et al., 2013; Pinu et al., 2017). In *C. albicans* biofilms, a great number of enzymes were existent with extensively diverse biological functions (Lattif et al., 2008). These enzymes originated from either abnormal intracellular release caused by fungal cell rupture or normal intracellular release via exosome transportation (Vargas et al., 2015; Nimrichter et al., 2016). It is odd, however, that we do not notice any report introducing quenching in bacterial or fungal biofilm EM extractions. We speculate that the current frequently-used quenching methods may cause huge losses of biofilm EM and have a severely negative impact on the following analyses of metabolites extracted.

### Enlightenment: Combining the Metabolites Extracted by Both the CER-VU and VU

The results of the untargeted metabolites exhibited that neither the CER-VU nor VU could extract the maximum amount of biological substances. On one hand, the relative number of specific metabolites in common (mean value in Supplementary Table S2) was greatly different in each method. Excluding 154 identical metabolites, on the other hand, there was still a large quantity of different extracts with both methods (63 versus 44, Figure 5). These discrepancies are suggestive of an idea mentioned before that comprehensive coverage of metabolites can be maximally realized by combining parallel extraction methods (Canelas et al., 2009). This phenomenon of disparity was firstly noted by Maharjan and Ferenci and Villas-Boas and colleagues Maharjan and Ferenci observed that different extraction methods had diverse performances, and the absence of one method could cover all metabolites based on the relative measurement of 13 metabolites (Maharjan and Ferenci, 2003). Villas-Boas et al. (2005) also found similar disparity among 27 metabolites and presented their results in the form of metabolites instead of individual compounds. These groups were aware of the difficulty in the development of a method to extract a great variety of metabolites due to a fact that each extraction performance was different for certain specific classes. Based on these observations, several groups proposed that high efficacies and recoveries of metabolites with at least two extraction methods should be well-considered (Mashego et al., 2007; Winder et al., 2008).

In summary, we introduced a series of methods to extract the EM of the static and dynamic *C. albicans* biofilms and showed that the CER-VU at pH 7.8 was the most effective condition for high-yield EM extractions with negligible loss of cell viability and integrity in *Candida* species. In the future, the performance of CER-VU should be adequately validated in different microbial species and growth conditions by more accurate determination (e.g., targeted filtration). From our perspective, firstly, the analysis of *Candida* biofilm EM should combine the results from two or even more extraction methods using diverse *in vitro* and *in vivo* biofilm models (Coenye and Nelis, 2010); secondly, more precise analytical instruments should be used to elucidate the complex structure of biofilm and the roles of biofilm EM substances under the dynamic state in *C. albicans*; thirdly, the interaction of *Candida* biofilm EM with immune cells (e.g., macrophage and neutrophil) can also be surveyed by analytical methods—for example, enzyme digestion of specific EM component. In short, the completion of this study expands our understanding on the matrix constituents of the dynamic biofilm in *C. albicans* and provides deep insight into antifungal prevention and treatment.

## Author Contributions

WD and QL performed the experiments. GS and TW analyzed the data. DW reviewed the whole manuscript. JS and CW devised the whole experiments. JS arranged the data and wrote the manuscript.

## Conflict of Interest Statement

The authors declare that the research was conducted in the absence of any commercial or financial relationships that could be construed as a potential conflict of interest.

## Footnotes

1 http://proteowizard.sourceforge.net/projects.html

2 http://enigma.lbl.gov/xcms-online/

3 https://www.kegg.jp/kegg/tool/map_pathway2.html

## References

[B1] Al-Fattani M. A. Douglas L. J. (2006). Biofilm matrix of *Candida albicans* and *Candida tropicalis*: chemical composition and role in drug resistance. *J. Med. Microbiol.* 55 999–1008. 10.1099/jmm.0.46569-0 16849719

[B2] Bjarnsholt T. Ciofu O. Molin S. Givskov M. Hoiby N. (2013). Applying insights from biofilm biology to drug development-can a new approach be developed? *Nat. Rev. Drug Discov.* 12 791–808. 10.1038/nrd4000 24080700

[B3] Brown G. D. Gordon S. (2003). Fungal beta glucans and mammalian immunity. *Immunity* 19 311–315. 10.1016/S1074-7613(03)00233-4 14499107

[B4] Brown M. J. Lester J. N. (1980). Comparison of bacterial extracellular polymer extraction methods. *Appl. Environ. Microbiol.* 40 179–185. 16345600 10.1128/aem.40.2.179-185.1980 PMC291550

[B5] Canelas A. B. Pierick A. T. Ras C. Seifar R. M. Dam J. C. V. Gulik W. M. V. (2009). Quantitative evaluation of intracellular metabolite extraction techniques for yeast metabolomics. *Anal. Chem.* 81 7379–7389. 10.1021/ac900999t 19653633

[B6] Chen X. Wu H. Cao Y. Yao X. Zhao L. Wang T. (2014). Ion-pairing chromatography on a porous graphitic carbon column coupled with time-of-flight mass spectrometry for targeted and untargeted profiling of amino acid biomarkers involved in *Candida albicans* biofilm formation. *Mol. BioSyst.* 10 74–85. 10.1039/c3mb70240e 24150280

[B7] Cheng M. F. Yang Y. L. Yao T. J. Lin C. Y. Liu J. S. Tang R. B. (2005). Risk factors for fatal candidemia caused by *Candida albicans* and non-*albicans Candida* species. *BMC Infect. Dis.* 5:22. 10.1186/1471-2334-5-22 15813977 PMC1090575

[B8] Coenye T. Nelis H. J. (2010). *In vitro* and *in vivo* model systems to study microbial biofilm formation. *J. Microbiol. Methods* 83 89–105. 10.1016/j.mimet.2010.08.018 20816706

[B9] Comte S. Guibaud G. Baudu M. (2006). Relations between extraction protocols for activated sludge extracellular polymeric substances (EPS) and EPS complexation properties: part I. Comparison of the efficiency of eight EPS extraction methods. *Enzyme. Microb. Technol.* 38 237–245. 10.1016/j.enzmictec.2005.06.016

[B10] Cuéllar-Cruz M. López-Romero E. Villagómez-Castro J. C. Ruiz-Baca E. (2012). *Candida* species: new insights into biofilm formation. *Future Microbiol.* 7 755–771. 10.2217/fmb.12.48 22702528

[B11] D’Abzac P. Bordas F. Van Hullebusch E. Lens P. N. Guibaud G. (2010). Extraction of extracellular polymeric substances (EPS) from anaerobic granular sludges: comparison of chemical and physical extraction protocols. *Appl. Microbiol. Biotechnol.* 85 1589–1599. 10.1007/s00253-009-2288-x 19862516

[B12] Dignac M. F. Urbain V. Rybacki D. Bruchet A. Snidaro D. Scribe P. (1998). Chemical description of extracellular polymers: implication on activated sludge floc structure. *Water Sci. Technol.* 38 45–53. 10.2166/wst.1998.0789

[B13] Duportet X. Aggio R. B. M. Carneiro S. N. Villas-Bˆas S. G. (2012). The biological interpretation of metabolomic data can be misled by the extraction method used. *Metabolomics* 8 410–421. 10.1007/s11306-011-0324-1

[B14] Frølund B. Palmgren R. Keiding K. Nielsen P. H. (1996). Extraction of extracellular polymers from activated sludge using a cation exchange resin. *Water Res.* 30 1749–1758. 10.1016/0043-1354(95)00323-1

[B15] Ghannoum M. A. Burns G. R. Elteen K. A. Radwan S. S. (1986). Experimental evidence for the role of lipids in adherence of *Candida* spp. to human buccal epithelial cells. *Infect. Immun.* 54 189–193. 3759234 10.1128/iai.54.1.189-193.1986 PMC260135

[B16] Gow N. A. Van De Veerdonk F. L. Brown A. J. Netea M. G. (2012). *Candida albicans* morphogenesis and host defence: discriminating invasion from colonization. *Nat. Rev. Microbiol.* 10 112–122. 10.1038/nrmicro2711 22158429 PMC3624162

[B17] Keppler-Ross S. Douglas L. Konopka J. B. Dean N. (2010). Recognition of yeast by murine macrophages requires mannan but not glucan. *Eukaryot. Cell* 9 1776–1787. 10.1128/EC.00156-10 20833894 PMC2976302

[B18] Kim S. Lee D. Y. Wohlgemuth G. Park H. S. Fiehn O. Kim K. H. (2013). Evaluation and optimization of metabolome sample preparation methods for *Saccharomyces cerevisiae*. *Anal. Chem.* 85 2169–2176. 10.1021/ac302881e 23289506

[B19] Lattif A. A. Chandra J. Chang J. Liu S. Zhou G. Chance M. R. (2008). Proteomics and pathway mapping analyses reveal phase-dependent over-expression of proteins associated with carbohydrate metabolic pathways in *Candida albicans* biofilms. *Open Proteom. J.* 1 5–26. 10.2174/1875039700801010005

[B20] Lattif A. A. Mukherjee P. K. Chandra J. Roth M. R. Welti R. Rouabhia M. (2011). Lipidomics of *Candida albicans* biofilms reveals phase-dependent production of phospholipid molecular classes and role for lipid rafts in biofilm formation. *Microbiology* 157 3232–3242. 10.1099/mic.0.051086-0 21903752 PMC3352276

[B21] Liu H. Fang H. H. (2002). Extraction of extracellular polymeric substances (EPS) of sludges. *J. Biotechnol.* 95 249–256. 10.1016/S0168-1656(02)00025-1 12007865

[B22] Lomb M. Fryberg M. Oehlschlager A. C. Unrau A. M. (1975). Sterol and fatty acid composition of polyene macrolide antibiotic resistant *Torulopsis glabrata*. *Can. J. Biochem.* 53 1309–1315. 10.1139/o75-177 1240787

[B23] Maharjan R. P. Ferenci T. (2003). Global metabolite analysis: the influence of extraction methodology on metabolome profiles of *Escherichia coli*. *Anal. Biochem.* 313 145–154. 10.1016/S0003-2697(02)00536-5 12576070

[B24] Martins M. Uppuluri P. Thomas D. P. Cleary I. A. Henriques M. Lopez-Ribot J. L. (2009). Presence of extracellular DNA in the *Candida albicans* biofilm matrix and its contribution to biofilms. *Mycopathologia* 169 323–331. 10.1007/s11046-009-9264-y 20012895 PMC3973130

[B25] Mashego M. R. Rumbold K. De Mey M. Vandamme E. Soetaert W. Heijnen J. J. (2007). Microbial metabolomics: past, present and future methodologies. *Biotechnol. Lett.* 29 1–16. 10.1007/s10529-006-9218-0 17091378

[B26] McKenzie C. G. J. Koser U. Lewis L. E. Bain J. M. Mora-Montes H. M. Barker R. N. (2010). Contribution of *Candida albicans* cell wall components to recognition by and escape from murine macrophages. *Infect. Immun.* 78 1650–1658. 10.1128/IAI.00001-10 20123707 PMC2849426

[B27] Mora-Montes H. M. Netea M. G. Ferwerda G. Lenardon M. D. Brown G. D. Mistry A. R. (2011). Recognition and blocking of innate immunity cells by *Candida albicans* chitin. *Infect. Immun.* 79 1961–1970. 10.1128/IAI.01282-10 21357722 PMC3088140

[B28] Mukherjee P. K. Chandra J. Kuhn D. M. Ghannoum M. A. (2003). Mechanism of fluconazole resistance in *Candida albicans* biofilms: phase-specific role of efflux pumps and membrane sterols. *Infect. Immun.* 71 4333–4340. 10.1128/IAI.71.8.4333-4340.2003 12874310 PMC165995

[B29] Nimrichter L. De Souza M. M. Del Poeta M. Nosanchuk J. D. Joffe L. Tavares Pde M. (2016). Extracellular vesicle-associated transitory cell wall components and their impact on the interaction of fungi with host cells. *Front. Microbiol.* 7:1034. 10.3389/fmicb.2016.01034 27458437 PMC4937017

[B30] Pesti M. Horvath L. Vigh L. Farkas T. (1985). Lipid content and ESR determination of plasma membrane order parameter in *Candida albicans* sterol mutants. *Acta Microbiol. Hung.* 32 305–313. 3012928

[B31] Pierce C. G. Vila T. Romo J. A. Montelongo-Jauregui D. Wall G. Ramasubramanian A. (2017). The *Candida albicans* biofilm matrix: composition, structure and function. *J. Fungi* 3:14. 10.3390/jof3010014 28516088 PMC5431293

[B32] Pinu F. R. Villas-Boas S. G. Aggio R. (2017). Analysis of intracellular metabolites from microorganisms: quenching and extraction protocols. *Metabolites* 7:53. 10.3390/metabo7040053 29065530 PMC5746733

[B33] Rajendran R. Sherry L. Lappin D. F. Nile C. J. Smith K. Williams C. (2014). Extracellular DNA release confers heterogeneity in *Candida albicans* biofilm formation. *BMC Microbiol.* 14:303. 10.1186/s12866-014-0303-6 25476750 PMC4262977

[B34] Ratto M. Verhoef R. Suihko M. L. Blanco A. Schols H. A. Voragen A. G. (2006). Colanic acid is an exopolysaccharide common to many enterobacteria isolated from paper-machine slimes. *J. Ind. Microbiol. Biotechnol.* 33 359–367. 10.1007/s10295-005-0064-1 16418870

[B35] Schneider K. Kromer J. O. Wittmann C. Alves-Rodrigues I. Meyerhans A. Diez J. (2009). Metabolite profiling studies in *Saccharomyces cerevisiae*: an assisting tool to prioritize host targets for antiviral drug screening. *Microb. Cell Fact.* 8:12. 10.1186/1475-2859-8-12 19183481 PMC2658664

[B36] Shao J. Lu K. Q. Tian G. Cui Y. Y. Yan Y. Y. Wang T. M. (2015). Lab-scale preparations of *Candida albicans* and dual *Candida albicans*-*Candida glabrata* biofilms on the surface of medical-grade polyvinyl chloride (PVC) perfusion tube using a modified gravity-supported free-flow biofilm incubator (GS-FFBI). *J. Microbiol. Methods* 109 41–48. 10.1016/j.mimet.2014.12.006 25528294

[B37] Sun M. Li W. W. Yu H. Q. Harada H. (2012). A novel integrated approach to quantitatively evaluate the efficiency of extracellular polymeric substances (EPS) extraction process. *Appl. Microbiol. Biotechnol.* 96 1577–1585. 10.1007/s00253-012-4478-1 23064456

[B38] Taff H. T. Nett J. E. Zarnowski R. Ross K. M. Sanchez H. Cain M. T. (2012). A *Candida* biofilm-induced pathway for matrix glucan delivery: implications for drug resistance. *PLoS Pathog.* 8:e1002848. 10.1371/journal.ppat.1002848 22876186 PMC3410897

[B39] Tapia J. M. Munoz J. A. Gonzalez F. Blazquez M. L. Malki M. Ballester A. (2009). Extraction of extracellular polymeric substances from the acidophilic bacterium Acidiphilium 3.2Sup(5). *Water Sci. Technol.* 59 1959–1967. 10.2166/wst.2009.192 19474490

[B40] Tobudic S. Kratzer C. Lassnigg A. Presterl E. (2012). Antifungal susceptibility of *Candida albicans* in biofilms. *Mycoses* 55 199–204. 10.1111/j.1439-0507.2011.02076.x 21793943

[B41] Uppuluri P. Chaturvedi A. K. Lopez-Ribot J. L. (2009). Design of a simple model of *Candida albicans* biofilms formed under conditions of flow: development, architecture, and drug resistance. *Mycopathologia* 168 101–109. 10.1007/s11046-009-9205-9 19370400 PMC3972753

[B42] Uppuluri P. Lopez-Ribot J. L. (2010). An easy and economical in vitro method for the formation of *Candida albicans* biofilms under continuous conditions of flow. *Virulence* 1 483–487. 10.4161/viru.1.6.13186 21178492 PMC3073357

[B43] Vargas G. Rocha J. D. Oliveira D. L. Albuquerque P. C. Frases S. Santos S. S. (2015). Compositional and immunobiological analyses of extracellular vesicles released by *Candida albicans*. *Cell. Microbiol.* 17 389–407. 10.1111/cmi.12374 25287304

[B44] Vertes A. Hitchins V. Phillips K. S. (2012). Analytical challenges of microbial biofilms on medical devices. *Anal. Chem.* 84 3858–3866. 10.1021/ac2029997 22424152

[B45] Villas-Boas S. G. Hojer-Pedersen J. Akesson M. Smedsgaard J. Nielsen J. (2005). Global metabolite analysis of yeast: evaluation of sample preparation methods. *Yeast* 22 1155–1169. 10.1002/yea.1308 16240456

[B46] Vitali A. Vavala E. Marzano V. Leone C. Castagnola M. Iavarone F. (2017). Cell wall composition and biofilm formation of azoles-susceptible and -resistant *Candida glabrata* strains. *J. Chemother.* 29 164–172. 10.1080/1120009X.2016.1199507 27439026

[B47] Winder C. L. Dunn W. B. Schuler S. Broadhurst D. Jarvis R. Stephens G. M. (2008). Global metabolic profiling of *Escherichia coli* cultures: an evaluation of methods for quenching and extraction of intracellular metabolites. *Anal. Chem.* 80 2939–2948. 10.1021/ac7023409 18331064

[B48] Wu G. (2009). Amino acids: metabolism, functions, and nutrition. *Amino Acids* 37 1–17. 10.1007/s00726-009-0269-0 19301095

[B49] Yeater K. M. Chandra J. Cheng G. Mukherjee P. K. Zhao X. Rodriguez-Zas S. L. (2007). Temporal analysis of *Candida albicans* gene expression during biofilm development. *Microbiology* 153 2373–2385. 10.1099/mic.0.2007/006163-0 17660402

[B50] Zarnowski R. Sanchez H. Andes D. R. (2016). Large-scale production and isolation of *Candida* biofilm extracellular matrix. *Nat. Protoc.* 11 2320–2327. 10.1038/nprot.2016.132 27809313

[B51] Zarnowski R. Westler W. M. Lacmbouh G. A. Marita J. M. Bothe J. R. Bernhardt J. (2014). Novel entries in a fungal biofilm matrix encyclopedia. *mBio* 5:e1333-14. 10.1128/mBio.01333-14 25096878 PMC4128356

[B52] Zhao M. Du L. Tao J. Qian D. Shang E. X. Jiang S. (2014). Determination of metabolites of diosmetin-7-O-glucoside by a newly isolated *Escherichia coli* from human gut using UPLC-Q-TOF/MS. *J. Agric. Food Chem.* 62 11441–11448. 10.1021/jf502676j 25382172

