# Supplementary material for: Extraction of Extracellular Matrix in Static and Dynamic Candida Biofilms Using Cation Exchange Resin and Untargeted Analysis of Matrix Metabolites by Ultra-High-Performance Liquid Chromatography-Tandem Quadrupole Time-of-Flight Mass Spectrometry (UPLC-Q-TOF-MS)
Source: Front Microbiol. 2019 Apr 10;10:752. doi: 10.3389/fmicb.2019.00752 (PMC6499207; doi:10.3389/fmicb.2019.00752)
Supplement: Supplementary file 1 [file Table_1.DOC]

**Supplemental Materials**

**Submitted to:** Frontiers in Microbiology

Extraction of extracellular matrix in static and dynamic *Candida* biofilms using cation exchange resin and untargeted analysis of matrix metabolites by ultra-high performance liquid chromatography-tandem quadrupole time-of-flight mass spectrometry (UPLC-Q-TOF-MS)

**Running title:** Extraction and analysis of Candida biofilm EM

Wenyue Da^1^,^♀^, Jing Shao^1,♀,*^, Qianqian Li^1^, Gaoxiang Shi^1^, Tianming Wang^1^, Daqiang, Wu^1^, Changzhong Wang^1,*^

^1^Laboratory of Pathogenic Biology and Immunology, College of Integrated Chinese and Western Medicine (College of Life Science), Anhui University of Chinese Medicine, 436 Room, Zhijing Building, No. 1 Qianjiang Road, Xinzhan District, Hefei 230012, Anhui, China;

^♀^ The authors contribute equally to this paper.

^*^ Corresponding authors: Dr. Jing Shao, E-mail: ustcnjnusjtu@126.com; Prof. Changzhong Wang, E-mail: wangchangzhong53@126.com; Tel/Fax: +86-551-6812-9457.

**Materials and methods**

**Non-*albicans Candida* species and cultivation.** *Candida tropicalis* ATCC750, *Candida glabrata* ATCC15126 and *Candida krusei* ATCC1182 were purchased from Bianzhen Biotech. Co. (Nanjing, China). The cultivation procedures were the same as those of *C. albicans* SC5314. The initial inoculum of the non-albicans Candida (NAC) species were of 4-6×10^6^ CFU/mL and the experimental procedures for static and flow biofilm formations of NAC could be referred to those of *C. albicans* SC5314.

**Results**


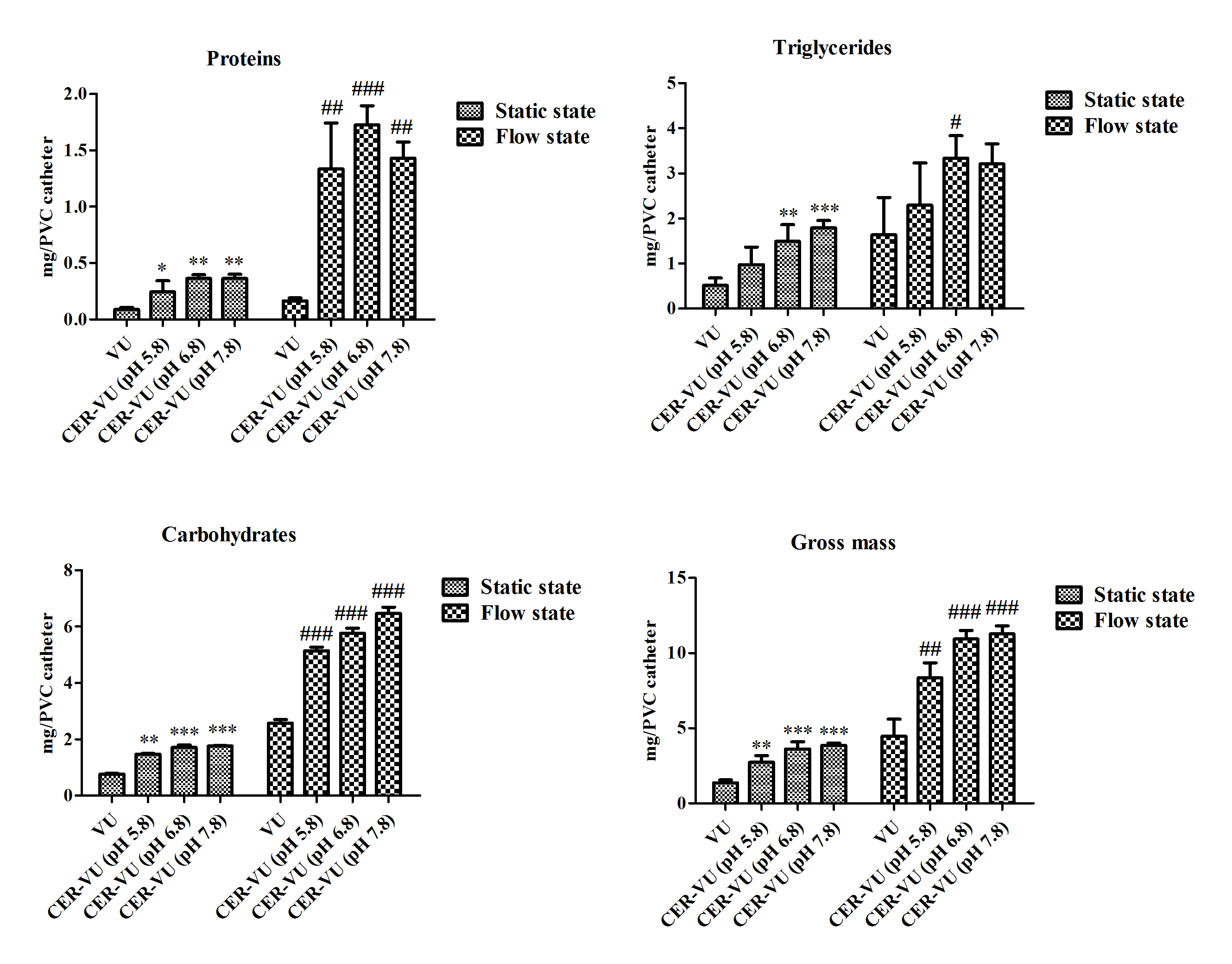


**Figure S1.** Biofilm EPS extraction of *Candida glabrata* ATCC15126 with VU and CER-VU methods at pH 5.8, 6.8 and 7.8 under static and flow states. *, p<0.05; **, p<0.01; ***, p<0.001; compared with VU method under static state. #, p<0.05; ##, p<0.01; ###, p<0.001; compared with VU method under flow state.


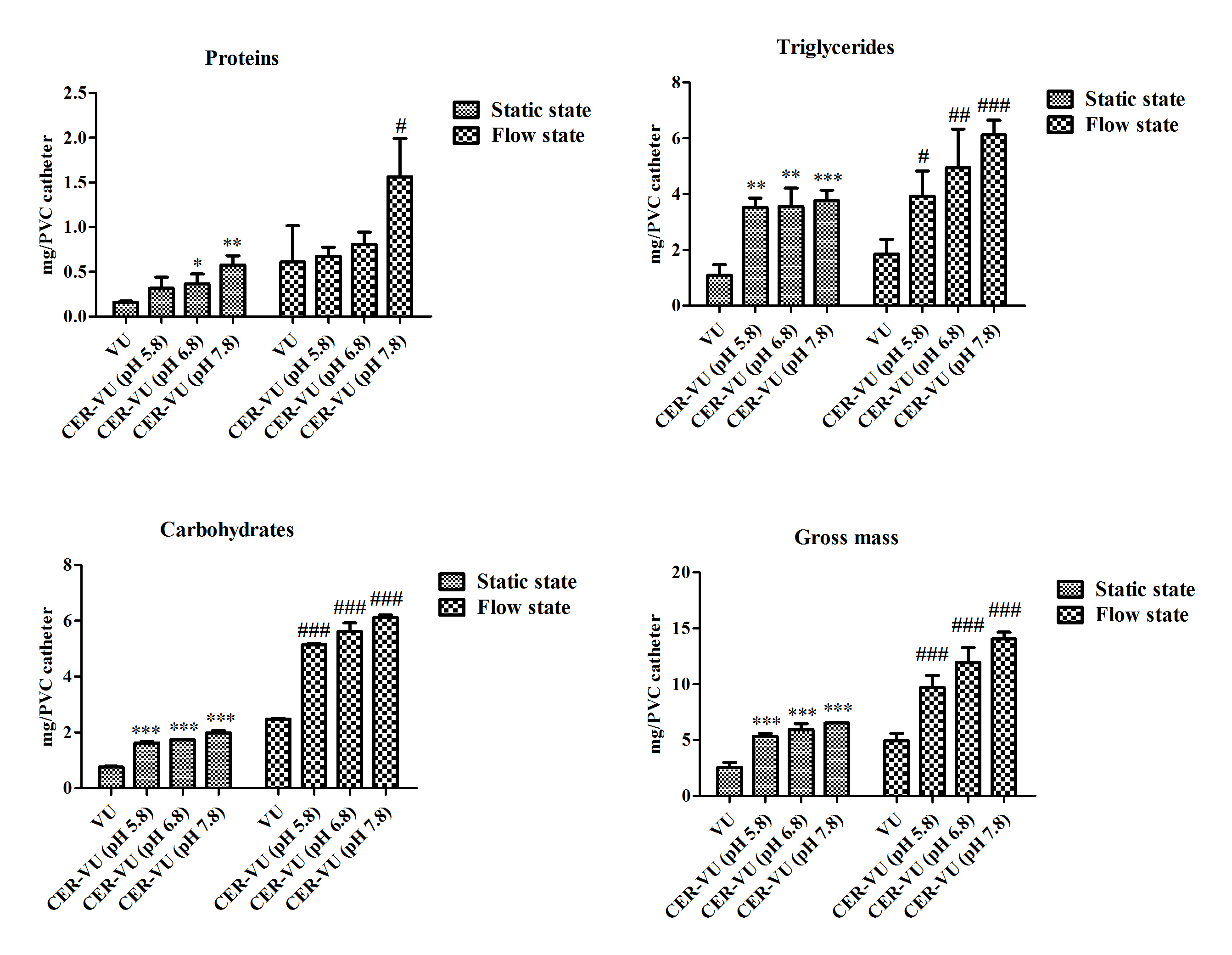


**Figure S2.** Biofilm EPS extraction of *Candida krusei* ATCC1182 with VU and CER-VU methods at pH 5.8, 6.8 and 7.8 under static and flow states. *, p<0.05; **, p<0.01; ***, p<0.001; compared with VU method under static state. #, p<0.05; ##, p<0.01; ###, p<0.001; compared with VU method under flow state.


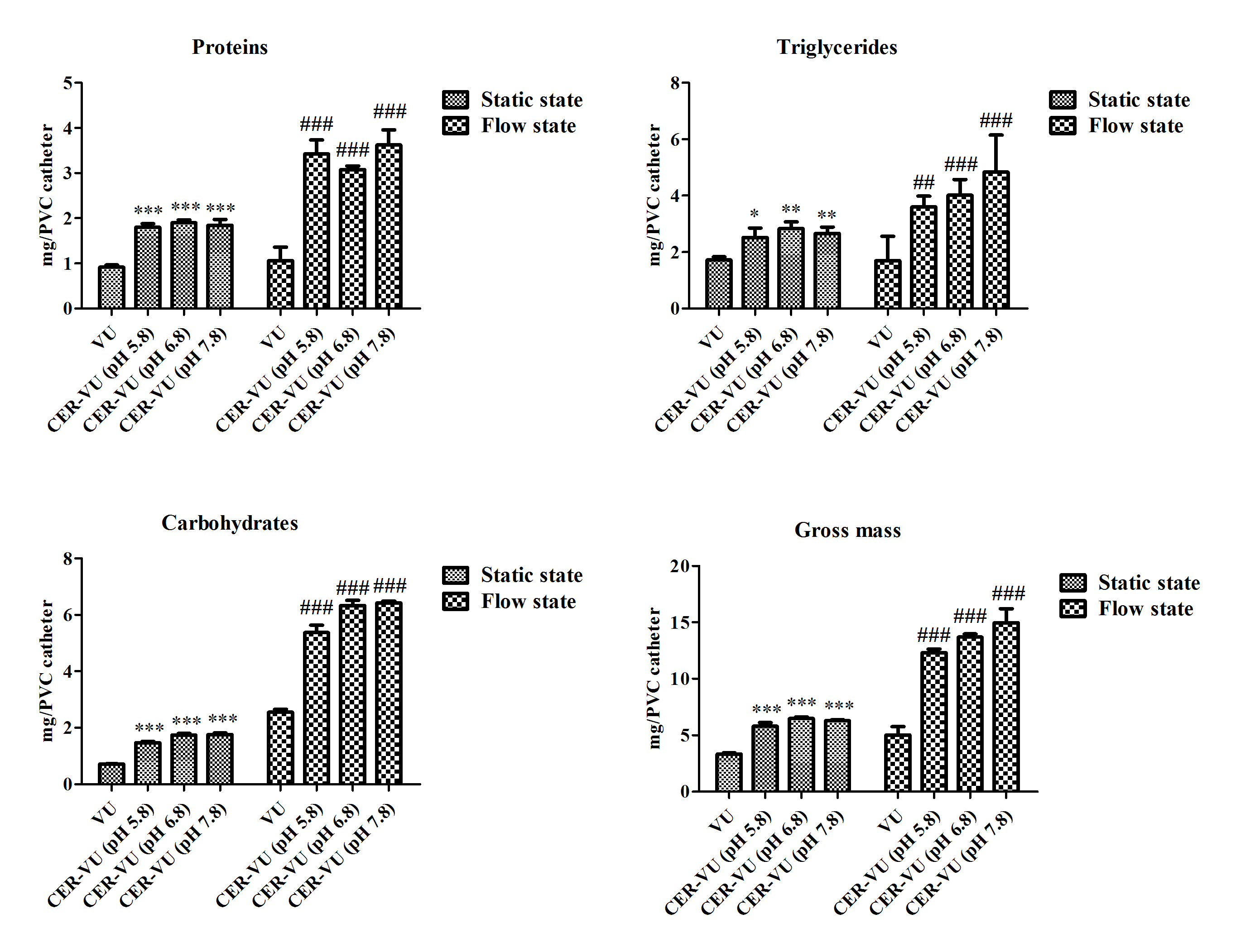


**Figure S3.** Biofilm EPS extraction of *Candida tropicalis* ATCC750 with VU and CER-VU methods at pH 5.8, 6.8 and 7.8 under static and flow states. *, p<0.05; **, p<0.01; ***, p<0.001; compared with VU method under static state. #, p<0.05; ##, p<0.01; ###, p<0.001; compared with VU method under flow state.


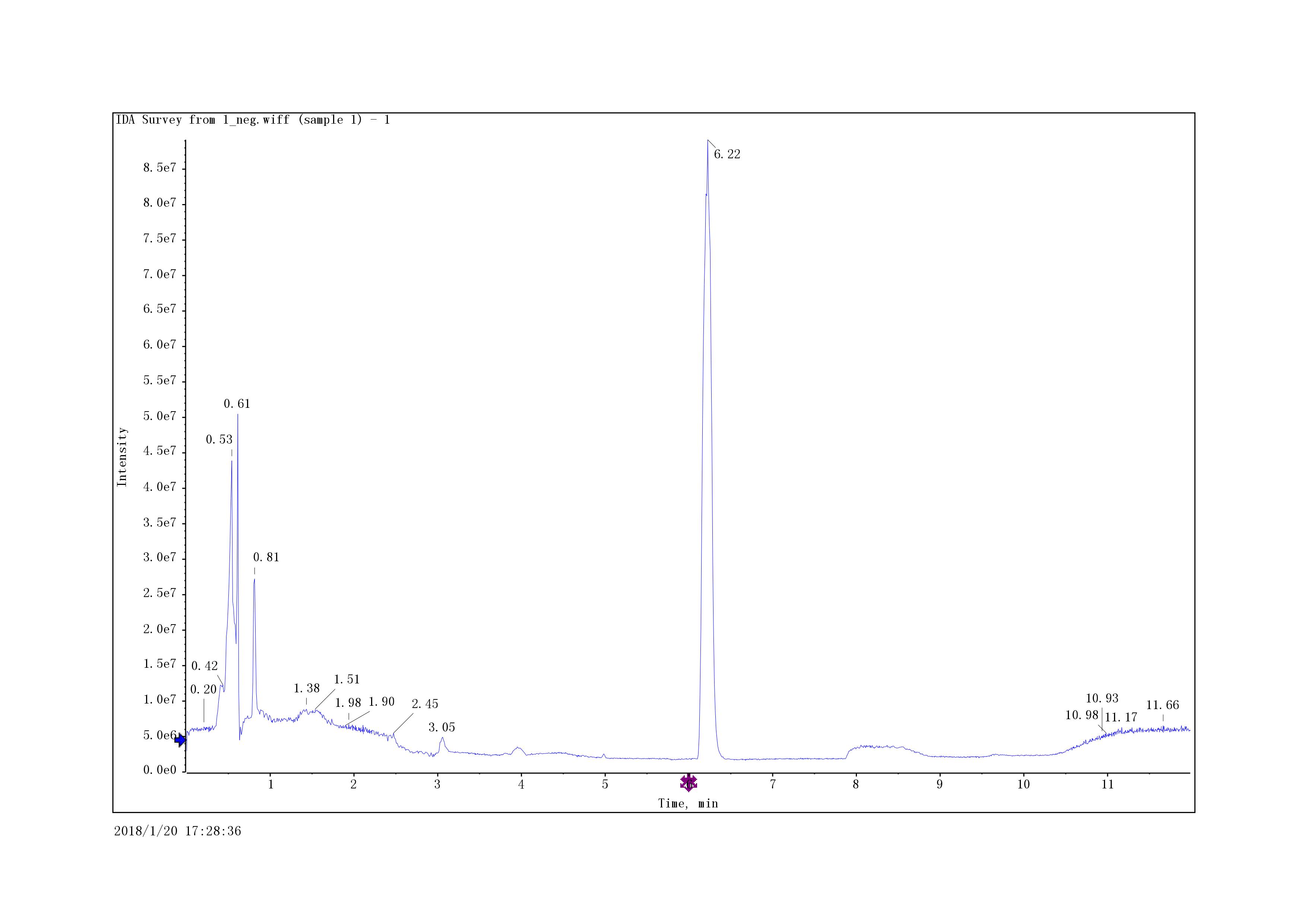


**Figure S4.** Total negative ion flow with CER-VU method


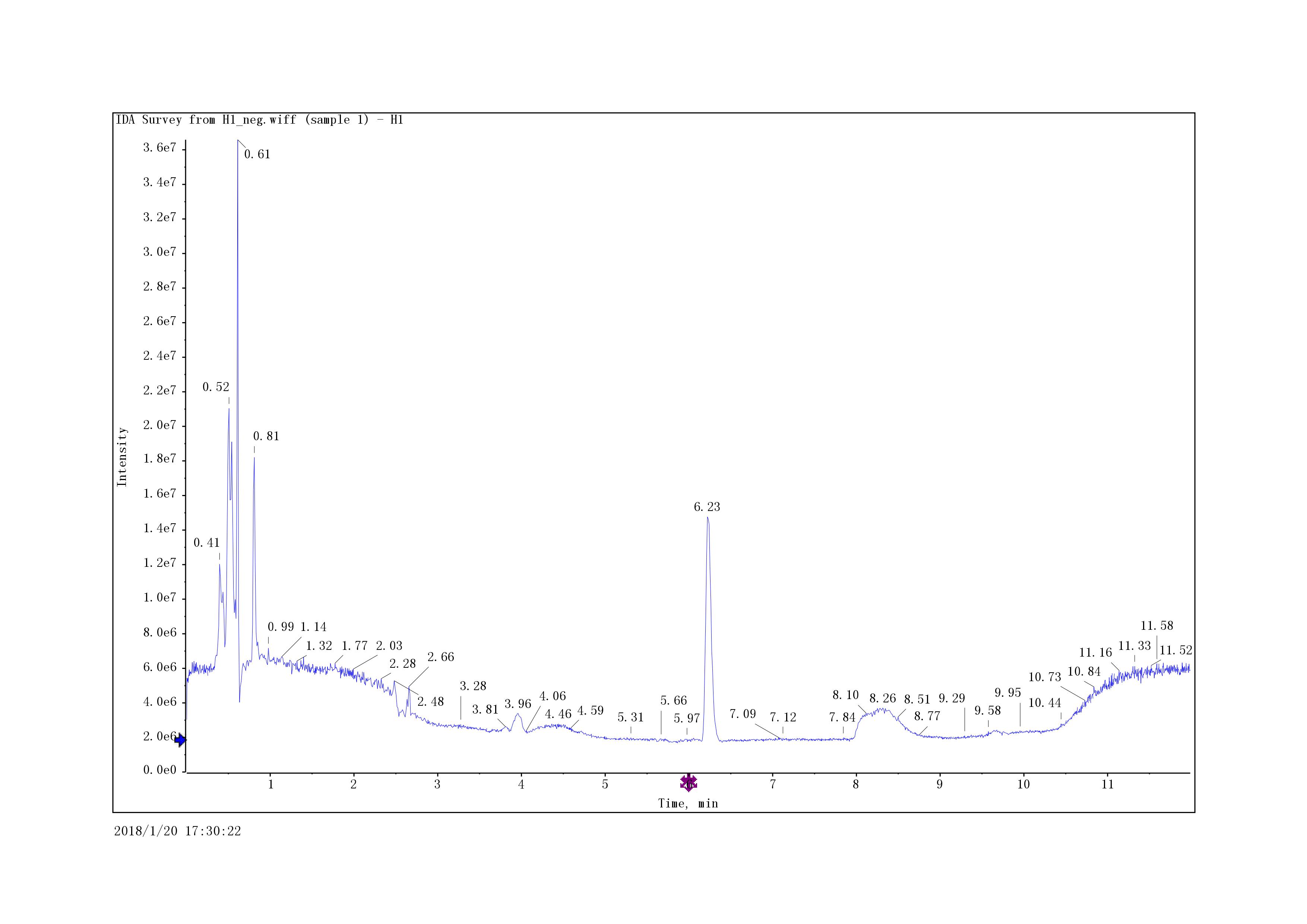


**Figure S5.** Total negative ion flow with VU method.


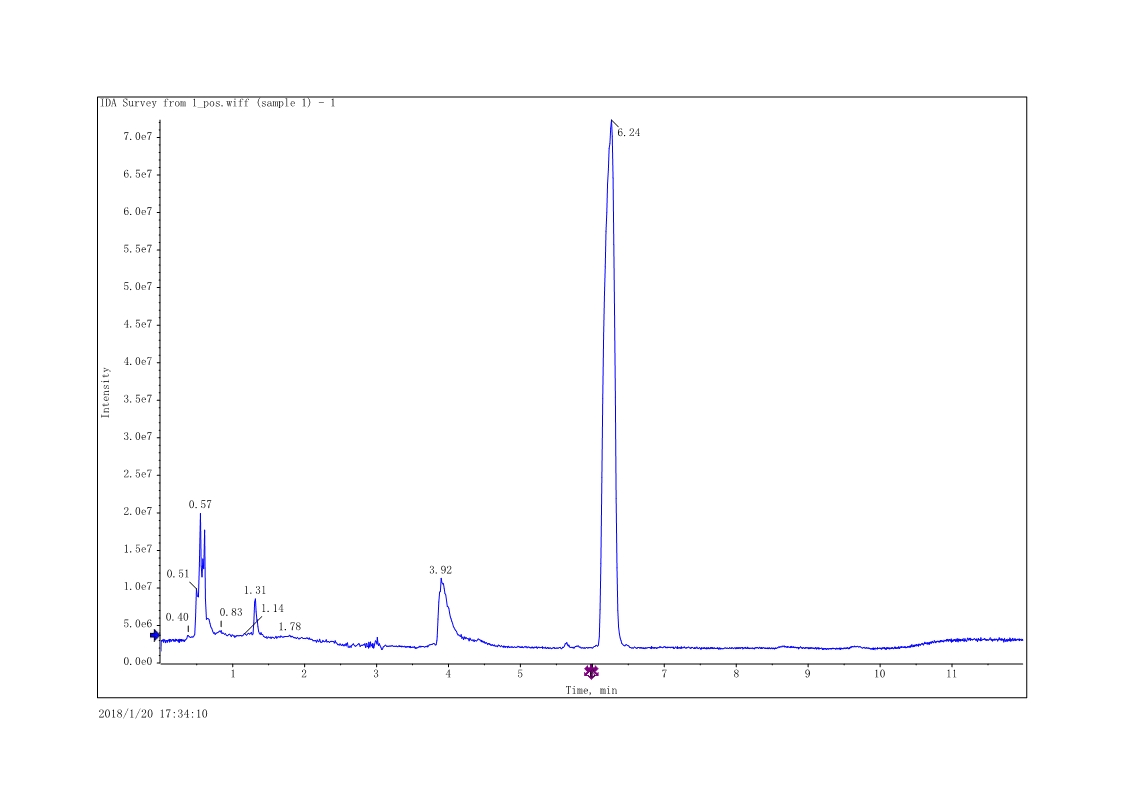


**Figure S6.** Total positive ion flow with CER-VU method.


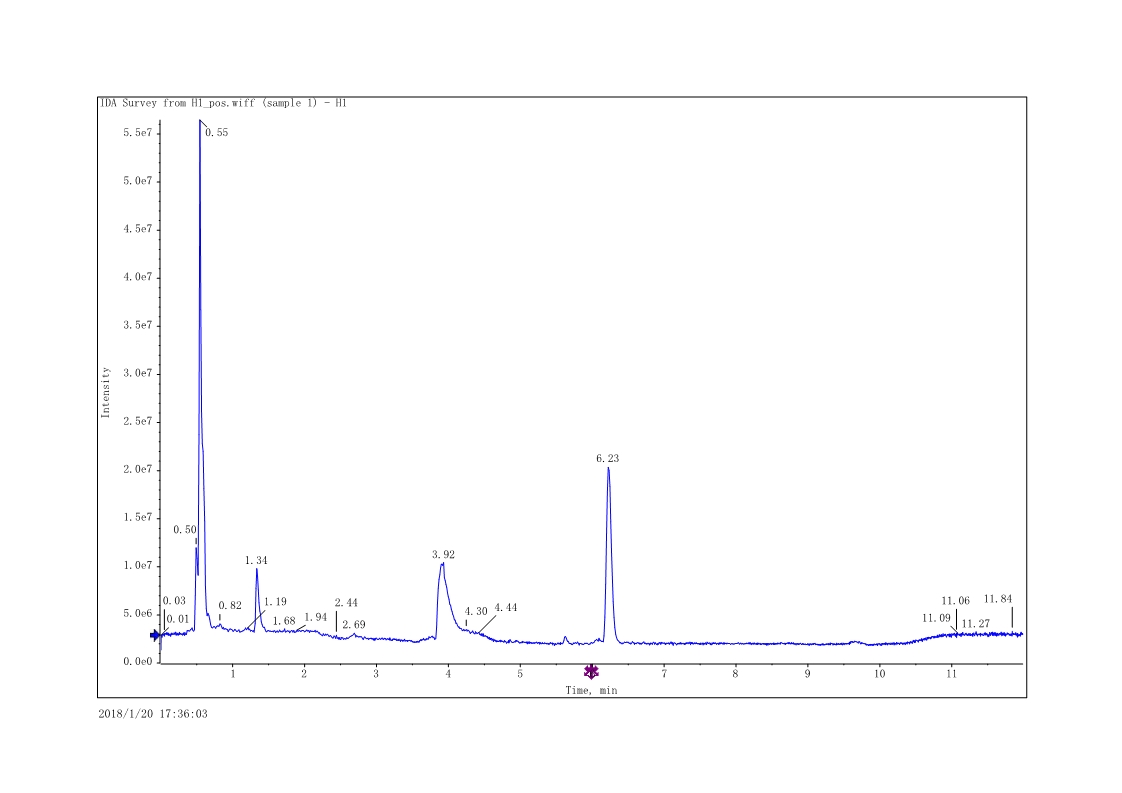


**Figure S7.** Total positive ion flow with VU method.


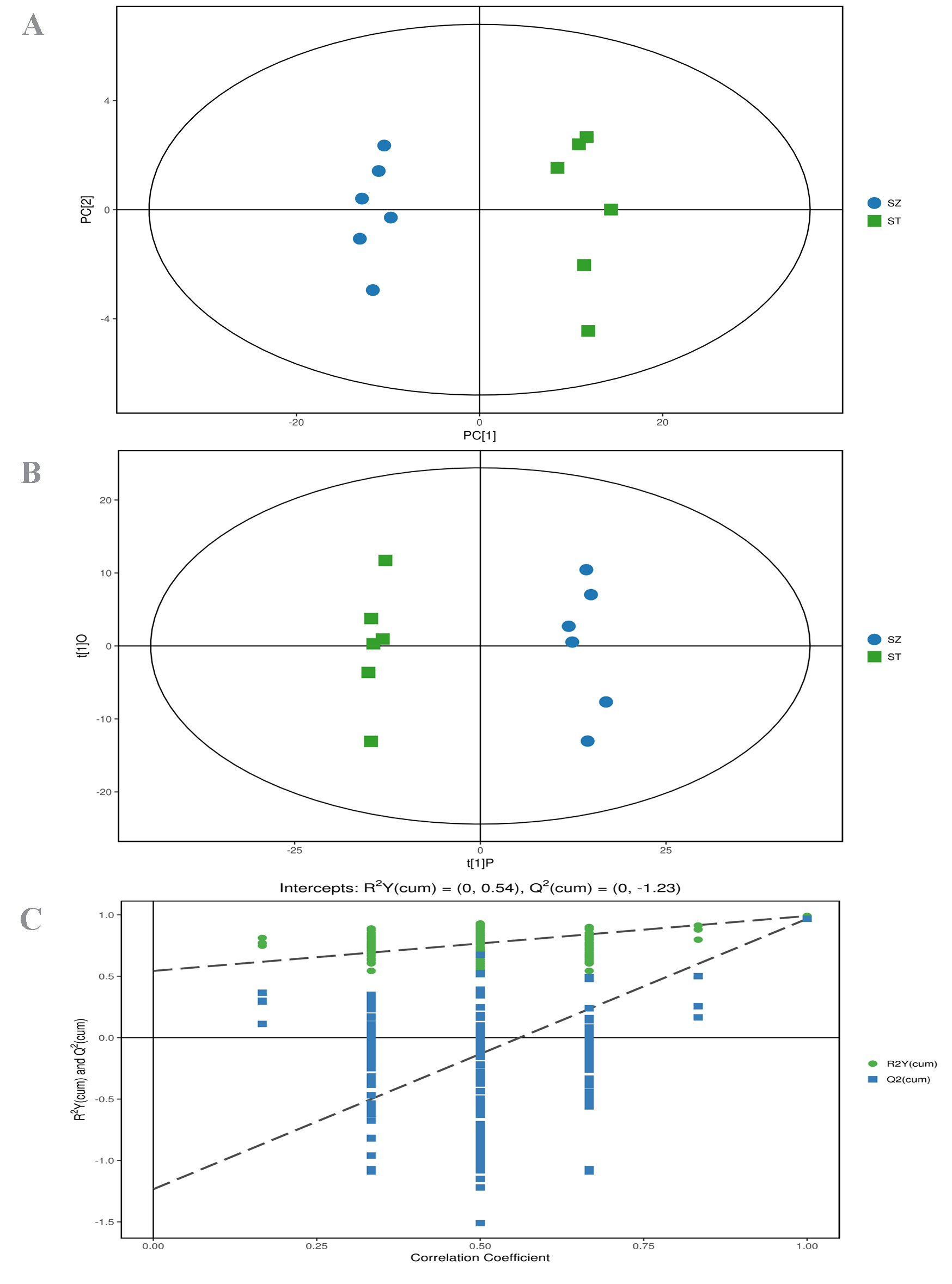


**Figure S8.** (A) PCA score plot, (B) OPLS-DA score plot, (C) OPLS-DA permutation plot of SZ versus ST in negative mode.


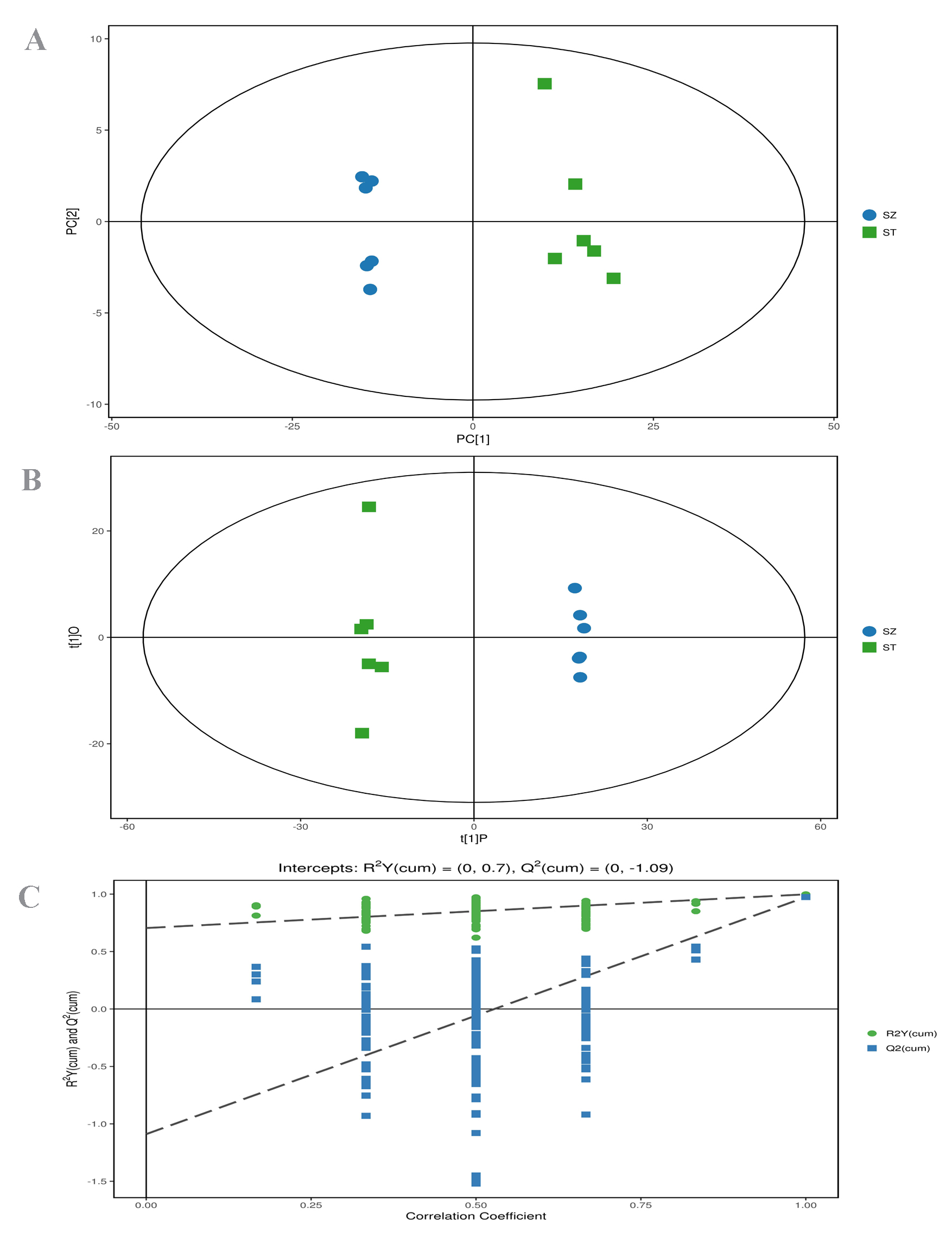


**Figure S9.** (A) PCA score plot, (B) OPLS-DA score plot, (C) OPLS-DA permutation plot of SZ versus ST in positive mode.

**Table S1.** Comparisons of extraction methods commonly used in EPS extractions of bacterial/fungal biofilms and activated sludges with vary sources.

| Extracted target | Extracted substance | | | | Extraction method/evaluation | | | | | | | | Reason | Ref. |
| --- | --- | --- | --- | --- | --- | --- | --- | --- | --- | --- | --- | --- | --- | --- |
|  | Protein | Carbohydrate | Lipids | DNA | 1.Filtration and  ultrasonication | 2.Dialysis | 3.Steaming | 4.Heating | 5.CER | 6.NaOH | 7.EDTA | 8.Formaldehyde plus |  |  |
| Activated sludge | √ | √ |  |  | + | + | ++ | ++ | +++ | +++ |  |  | Method 6 extracted prolific EPS and caused less-cellular disruption. | 1 |
| Activated sludge | √ | √ |  |  | + (only  ultrasonication) |  |  | ++ | +++ | + |  |  | Method 5 had high yield and minimal disruption of EPS. | 2 |
| Activated sludge | √ | √ | √ | √ |  |  |  | ++ |  |  |  | +++ (plus ultrasonication+NaOH) | Method 8 facilitated the exposure of inclusive EPS and loosen the EPS from cell walls. | 3 |
| Activated sludge | √ | √ |  | √ |  |  |  | ++ |  |  | +++ | +++( plus NaOH) | Method 8 prevented cell lysis and increased the EPS solubility | 4 |
| Activated sludge | √ | √ |  | √ |  |  |  |  | ++ | +++ | ++ |  | Method 6 owned prolific EPS and less-cellular disruption. | 5 |
| Aerobic/sulfalereducing and mtrifyingldenitrifying biofilms | √ | √ | √ | √ | + (only  ultrasonication) |  | ++ |  |  |  | + | +++ (plus centrifugation) | Method 8 had the greatest yield. | 6 |
| Aerobic activated sludge | √ | √ |  | √ |  |  |  |  |  |  |  | +++(plus NaOH and ultrasound) | Method 8 fixed the cell walls to prevent lysis and increased local pH to dissociate acidic groups in EPS. | 7 |
| Extreme acidic microbial biofilms | √ | √ |  | √ |  |  |  | +++ | +++ |  | ++ |  | ND | 8 |
| Alga-bacteria biofilms | √ | √ |  |  | + (only  ultrasonication) |  |  |  |  |  | +++ | +++ (plus NaOH) | ND | 9 |
| Microcystis wesenbergii | √ | √ |  |  |  | ++ |  | ++ | +++ | +++ |  |  | ND | 10 |
| Activated sludge | √ | √ |  | √ | + (only  ultrasonication) |  | +++ |  |  |  | +++ |  | Method 3 had high yield and minimal disruption of EPS | 11 |
| Acidophilic bacterium Acidiphilium | √ | √ |  |  |  |  |  | +++ | + | ++ | +++ |  | Method 7 possessed greater yield and less disruption | 12 |
| Bacillus megaterium TF10 | √ | √ |  | √ | + (only  ultrasonication) |  |  | ++ |  |  | +++ | +++ (plus NaOH) | Method led to less cell lysis and higher active constituents | 13 |
| Thermoacidophilic archaeon Sulfolobus acidocaldarius | √ | √ |  | √ |  |  |  |  | +++ | +++ | + |  | Method 5 produced high EPS yields with no apparent cell lysis and no impact on subsequent biochemical analysis. | 14 |
| Candida albicans Biofilms | √ | √ | √ | √ | ++ (only  ultrasonication) |  |  |  |  |  |  |  | Method 1 does not damage the cell structure | 15 |
| Candida albicans Biofilms | √ | √ |  | √ | + (only filtration) |  |  |  |  |  | +++ |  | Method 7 extracted the highest protein content with no effect on cell viability | 16 |

Legend: +, poor/bad; ++, fair; +++, good; underlined, the best among the methods tested.

Reference

1. Rudd T., Sterritt R. M., Lester J. N. Biotechnology Letters 1963, 5, 327-332.
2. Bo F., Palmgren R., Keiding K., et al. Water Research 1996, 30, 1749-1756.
3. Adav S. S., Lee D. J. Journal of Hazardous Materials 2006, 154, 1120-1022
4. Zhang L., Ren H., Ding L. Water Science & Technology 2012, 66, 1556-1564.
5. Sajjad M., Kim K. S. International Journal of Environmental Science & Technology 2016, 13, 1697-1706.
6. Zhang X., Bishop P. L., Kinkle B. K. Water Science & Technology 1999, 39, 211-216.
7. Adav S. S., Lee D. J. Journal of Hazardous Materials 2008, 154, 1120.
8. Aguilera A., Souza-Egipsy V., Martín-Úriz P. S., et al. Applied Microbiology & Biotechnology 2006, 76, 1079-1066.
9. Pan X. L., Jing L., Zhang D. Y., et al. Water Sa 2010, 36, 111-116.
10. Liu L., Qin B., Zhang Y., et al. Journal of Environmental Sciences 2014, 26, 1725-1732.
11. Brown M. J., Lester J. N. Applied & Environmental Microbiology 1960, 40, 179-165.
12. Tapia J. M., Muñoz J. A., González F., et al. Water Science & Technology 2009, 59, 1959-1967.
13. Sun M., Li W. W., Mu Z. X., et al. Separation & Purification Technology 2012, 95, 216-221.
14. Jachlewski S., Jachlewski W. D., Linne U., et al. Frontiers in Bioengineering & Biotechnology 2015, 3, 123.
15. Zarnowski R., Westler W. M., Lacmbouh G. A., et al. Mbio 2014, 5, 01333-14.
16. Lattif C A. A., Chandra J., Chang J., et al. Open Proteomics Journal 2006, 1, 5-26.

**Table S2.** Untargeted metabolites included in this study.

| **id** | **MS2 name** | **labid** | **m/z** | **rt** | **Exact Mass** | **formula** | **HMDB** | **PubChem** | **ChEBI** | **KEGG COMPOUND ID** | **METLIN** | **CAS** | **Mean** | **class** |
| --- | --- | --- | --- | --- | --- | --- | --- | --- | --- | --- | --- | --- | --- | --- |
| SZ-Metabolite mapping | | | | | | | | | | | | | | |
| 29 | 2-Amino-2-methyl-1,3-propanediol |  | 70.0644031 | 264.2345 | 105.14 | C4H11NO2 |  |  |  |  |  |  | 0.01193529 |  |
| 69 | Dimethylformamide |  | 74.0599817 | 23.972 | 73.1 | C3H7NO |  |  |  |  |  |  | 0.028884818 |  |
| 125 | L-Threonine | S0045 | 84.0434667 | 362.747 | 119.05824 | C4H9NO3 | HMDB00167 | 6288 | 16857 | C00188 | 32 | 72-19-5 | 0.004789417 | Amino acids and derivatives |
| 66 | 1-Aminocyclopropanecarboxylic acid | n00070 | 84.0434705 | 363.419 | 101.04768 | C4H7NO2 | HMDB36458 | 133503 |  |  |  | 22059-21-8 | 0.004789417 |  |
| 159 | Erythritol | L0435 | 87.0430371 | 2.554 | 122.05791 | C4H10O4 | HMDB02994 | 222285 | 17113 | C00503 | 140 | 149-32-6 | 0.003099785 | Carbohydrates and derivatives |
| 158 | Diacetyl | L0440 | 87.0430834 | 46.747 | 86.03678 | C4H6O2 | HMDB03407 | 650 | 16583 | C00741 | 6921 | 431-03-8 | 0.00704332 |  |
| 131 | DL-lactate | S0075 | 89.0240792 | 46.028 | 90.03169 | C3H6O3 | HMDB01311/HMDB00190 | 9378 | 17385 |  | 5723 | 50-21-5 | 0.022199878 |  |
| 183 | L-Alanine | S0087 | 90.0539444 | 338.9715 | 89.04768 | C3H7NO2 | HMDB00161 | 5950 | 16977 | C00041 |  | 56-41-7 | 0.007224394 | Amino acids and derivatives |
| 98 | Sarcosine | S0074 | 90.0539577 | 338.996 | 89.04768 | C3H7NO2 | HMDB00271 | 1088 | 15611 | C00213 | 51 | 107-97-1 | 0.007224394 | Amino acids and derivatives |
| 260 | DL-Norvaline |  | 100.075231 | 19.433 | 117.15 | C5H11NO2 |  |  |  |  |  |  | 0.090986785 | Amino acids and derivatives |
| 145 | DL-2,4-Diaminobutyric acid |  | 101.069346 | 363.419 | 118.1344 | C4H10N2O2 |  |  |  |  |  |  | 0.000331074 |  |
| 168 | L-2-Aminobutyric acid |  | 104.068859 | 326.602 | 103.12 | C4H9NO2 |  |  |  |  |  |  | 0.000768976 |  |
| 321 | Dimethylglycine | L0268 | 104.06896 | 283.867 | 103.06333 | C4H9NO2 | HMDB00092 | 673 | 17724 | C01026 | 277 | 1118-68-9 | 0.007725599 | Amino acids and derivatives |
| 328 | Choline | S0110 | 104.10534 | 378.7175 | 104.10754 | C5H14NO | HMDB00097 | 305 | 15354 | C00114 | 56 | 62-49-7 (67-48-1) | 0.014757001 | Lipids and derivatives |
| 182 | Diethanolamine | n00109 | 106.084524 | 320.231 | 105.07898 | C4H11NO2 | HMDB04437 | 8113 | 28123 | C06772 | 3239 | 111-42-2 | 0.001705357 | Lipids and derivatives |
| 368 | Benzylazanium |  | 108.079117 | 5.2755 |  |  |  |  |  |  |  |  | 0.004873013 |  |
| 183 | Uracil | S0002 | 111.019801 | 217.522 | 112.02728 | C4H4N2O2 | HMDB00300 | 1174 | 17568 | C00106 | 258 | 66-22-8 | 0.002341716 | Nucleic acids and derivatives |
| 224 | Creatinine | S0042 | 114.064518 | 338.996 | 113.05891 | C4H7N3O | HMDB00562 | 588 | 16737 | C00791 | 8 | 60-27-5 | 0.001537658 | Amino acids and derivatives |
| 429 | epsilon-Captrolactone |  | 114.067343 | 27.252 | 114.14 | C6H10O2 |  |  |  |  |  |  | 0.002058931 |  |
| 430 | epsilon-Caprolactam |  | 114.089967 | 47.5135 | 113.16 | C6H11NO |  |  |  |  |  |  | 0.027909082 |  |
| 471 | L-Proline | S0088 | 116.068993 | 306.047 | 115.06333 | C5H9NO2 | HMDB00162 | 145742 | 17203 | C00148 | 29 | 147-85-3 | 0.002978809 | Amino acids and derivatives |
| 239 | D-Proline | S0043 | 116.069072 | 304.477 | 115.06333 | C5H9NO2 | HMDB03411 | 8988 | 16313 | C00763 |  | 344-25-2 | 0.013209248 | Amino acids and derivatives |
| 227 | Succinate | S0006 | 117.019283 | 364.931 | 118.02661 | C4H6O4 | HMDB00254 | 1110 | 15741 | C00042 | 114 | 110-15-6 | 0.011003344 |  |
| 508 | Betaine | L0010 | 118.084627 | 432.356 | 117.07898 | C5H11NO2 | HMDB00043 | 247 | 17750 | C00719 | 287 | 107-43-7 (590-46-5) | 0.055851004 |  |
| 522 | 3-Hydroxyisovaleric acid | S0198 | 119.071407 | 235.381 | 118.063 | C5H10O3 | HMDB00754 | 69362 | 37084 |  | 5722 | 625-08-1 | 0.029642764 | Amino acids and derivatives |
| 402 | Benzoic acid | S0321 | 121.029505 | 248.084 | 122.03678 | C7H6O2 | HMDB01870 | 243 | 30746 | C00180 | 1297 | 65-85-0 | 0.09215585 |  |
| 551 | Benzamide |  | 122.058635 | 5.7545 | 121.14 | C7H7NO |  |  |  |  |  |  | 0.000602745 |  |
| 556 | Ethanolamine | S0036 | 122.07966 | 283.265 | 61.05276 | C2H7NO | HMDB00149 | 700 | 16000 | C00189 | 3207 | 141-43-5 | 0.001705357 | Lipids and derivatives |
| 559 | N,N-Dimethylaniline | n00178 | 122.094862 | 47.0445 | 121.08915 | C8H11N | HMDB01020 | 949 | 16269 | C02846 | 5949 | 121-69-7 | 0.001716064 |  |
| 565 | Nicotinamide | S0048 | 123.053473 | 62.3425 | 122.04801 | C6H6N2O | HMDB01406 | 936 | 17154 | C00153 | 1497 | 98-92-0 | 0.002800197 | Nucleic acids and derivatives |
| 577 | Picolinic acid | n00193 | 124.03746 | 201.707 | 123.03203 | C6H5NO2 | HMDB02243 | 1018 | 28747 | C10164 |  | 98-98-6 | 0.002230307 |  |
| 607 | Taurine | S0009 | 126.020074 | 286.343 | 125.01467 | C2H7NO3S | HMDB00251 | 1123 | 15891 | C00245 | 31 | 107-35-7 | 0.311501503 | Amino acids and derivatives |
| 494 | Glyceric acid | S0120 | 127.000807 | 373.864 | 106.02661 | C3H6O4 | HMDB00139 | 439194 | 32398 | C00258 | 280 | 473-81-4 | 0.106244639 | Lipids and derivatives |
| 499 | Dihydrothymine | S0158 | 127.051102 | 365.055 | 128.05858 | C5H8N2O2 | HMDB00079 | 93556 | 27468 | C00906 | 5135 | 696-04-8 | 0.005118804 | Nucleic acids and derivatives |
| 521 | Mevalonic acid | S0218 | 129.055615 | 18.513 | 148.07356 | C6H12O4 | HMDB00227 | 449 | 25351 | C00418 | 127 | 150-97-0 | 0.056290407 |  |
| 518 | Dihydroxyfumarate | L0455 | 129.05568 | 63 | 148.071 | C4H4O6 | HMDB02050 |  | 4593 | C00975 | 6460 |  | 0.064923493 |  |
| 519 | alpha-Ketocaproic acid | L0079 | 129.055694 | 43.537 | 130.063 | C6H10O3 | HMDB01864 | 159664 | 17308 | C00902 | 4144 | 2492-75-3 (13022-85-0) | 0.032805651 |  |
| 341 | Imidazole | S0037 | 129.064479 | 352.91 | 68.03745 | C3H4N2 | HMDB01525 | 795 | 16069 | C01589 | 4190 | 288-32-4 | 0.002738332 |  |
| 658 | Ala-Gly |  | 129.064511 | 352.6395 | 146.14 | C5H10N2O3 |  |  |  |  |  |  | 0.002738332 | Amino acids and derivatives |
| 672 | Pyroglutamic acid |  | 130.048426 | 386.904 | 129.12 | C5H7NO3 |  |  |  |  |  |  | 0.008534967 | Amino acids and derivatives |
| 351 | 3-Furancarboxylic acid |  | 130.048441 | 386.7395 | 112.08 | C5H4O3 |  |  |  |  |  |  | 0.003636564 |  |
| 681 | .beta.-Homoproline |  | 130.084645 | 331 |  |  |  |  |  |  |  |  | 0.00020579 | Amino acids and derivatives |
| 718 | Hydroxyproline | S0051 | 132.06388 | 359.125 | 131.05824 | C5H9NO3 | HMDB00725 | 5810 | 18095 | C01157 | 257 | 51-35-4 | 0.001234012 | Amino acids and derivatives |
| 719 | Creatine | S0047 | 132.076449 | 338.9585 | 131.06948 | C4H9N3O2 | HMDB00064 | 586 | 16919 | C00300 | 7 | 57-00-1 | 0.095024356 | Amino acids and derivatives |
| 722 | L-Isoleucine | S0089 | 132.100376 | 269.364 | 131.09463 | C6H13NO2 | HMDB00172 | 6306 | 17191 | C00407 | 5193 | 73-32-5 | 0.002268186 | Amino acids and derivatives |
| 723 | Pyrrolidine | n00006 | 132.100493 | 62.3425 | 71.0735 | C4H9N | HMDB31641 | 31268 | 33135 |  |  | 123-75-1 | 0.001549342 |  |
| 752 | Methyl acetoacetate |  | 134.079614 | 338.6615 | 117 | C5H8O3 |  |  |  |  |  |  | 0.000442194 |  |
| 780 | L-Threonate | L0376 | 136.037234 | 88.8045 | 136.03717 | C4H8O5 | HMDB00943 |  |  | C01620 |  | 70753-61-6 | 0.004512853 | Carbohydrates and derivatives |
| 783 | Adenine | S0054 | 136.059802 | 191.702 | 135.0545 | C5H5N5 | HMDB00034 | 190 | 16708 | C00147 | 85 | 73-24-5 | 0.001673882 | Nucleic acids and derivatives |
| 431 | Dopamine | L0111 | 136.074037 | 295.4525 | 153.07898 | C8H11NO2 | HMDB00073 | 681 | 18243 | C03758 | 64 | 51-61-6 (62-31-7) | 0.00072415 | Amino acids and derivatives |
| 805 | Hypoxanthine | S0012 | 137.044454 | 164.423 | 136.03851 | C5H4N4O | HMDB00157 | 790 | 17368 | C00262 | 83 | 68-94-0 | 0.003806936 | Nucleic acids and derivatives |
| 810 | Terpineol | n00423 | 137.130909 | 33.681 | 154.13576 | C10H18O | HMDB04043 | 442501 | 300 | C09902 |  | 98-55-5 | 0.000782114 |  |
| 654 | 4-Nitrophenol |  | 138.019684 | 44.818 | 139.11 | C6H5NO3 |  |  |  |  |  |  | 0.086922089 |  |
| 817 | Anthranilic acid (Vitamin L1) | S0363 | 138.053297 | 278.9085 | 137.04768 | C7H7NO2 | HMDB01123 | 227 | 30754 | C00108 | 6018 | 118-92-3 | 0.000444159 |  |
| 823 | Phenol | S0142 | 139.010743 | 235.0425 | 94.04187 | C6H6O | HMDB00228 | 996 | 15882 | C00146 | 128 | 108-95-2 | 0.001502583 |  |
| 826 | Urocanic acid | S0242 | 139.048653 | 274.395 | 138.04293 | C6H6N2O2 | HMDB00301 | 736715 | 30817 | C00785 | 298 | 104-98-3 | 0.000401849 | Amino acids and derivatives |
| 678 | 2-Oxoadipic acid | L0329 | 141.017953 | 373.37 | 160.03717 | C6H8O5 | HMDB00225 | 71 | 15753 | C00322 | 5234 | 3184-35-8 | 25.19438044 |  |
| 863 | 1,3-Diaminopropane | S0309 | 141.037759 | 223.027 | 74.0844 | C3H10N2 | HMDB00002 | 428 | 15725 | C00986 | 5081 | 109-76-2 | 0.008301345 |  |
| 935 | 3-(2-Hydroxyethyl)indole |  | 144.078974 | 87.031 | 161.2 | C10H11NO |  |  |  |  |  |  | 0.00340059 |  |
| 930 | Cyclohexylamine | n00061 | 144.079109 | 46.697 | 99.1048 | C6H13N | HMDB31404 | 7965 | 15773 | C00571 |  | 108-91-8 | 0.003117702 |  |
| 488 | Citraconic acid | n00245 | 146.04586 | 379.988 | 130.02661 | C5H6O4 | HMDB00634 | 643798 | 17626 | C02226 | 5607 | 498-23-7 | 0.003063507 |  |
| 547 | (3-Carboxypropyl)trimethylammonium cation |  | 146.11579 | 365.9825 | 210 | C9H20NO2.Cl |  |  |  |  |  |  | 7.4022E-05 |  |
| 976 | L-Glutamine | S0055 | 147.074772 | 362.7235 | 146.06914 | C5H10N2O3 | HMDB00641 | 5961 | 18050 | C00064 | 5614 | 56-85-9 | 0.023357657 | Amino acids and derivatives |
| 564 | Formylanthranilic acid | S0342 | 148.037842 | 46.713 | 165.04259 | C8H7NO3 | HMDB04089 | 101399 | 36575 | C05653 |  | 3342-77-6 | 0.000609605 |  |
| 582 | L-Methionine | S0057 | 150.056698 | 281.313 | 149.05105 | C5H11NO2S | HMDB00696 | 6137 | 16643 | C00073 | 5664 | 63-68-3 | 0.000613808 | Amino acids and derivatives |
| 1023 | Triethanolamine | n00366 | 150.111259 | 295.384 | 149.10519 | C6H15NO3 | HMDB32538 | 7618 | 28621 | C06771 |  | 102-71-6 | 0.002844054 | Lipids and derivatives |
| 810 | 6-Mercaptopurine |  | 151.006882 | 73.3015 | 152.18 | C5H4N4S |  |  |  |  |  |  | 0.047944938 | Nucleic acids and derivatives |
| 1054 | 2-Hydroxyadenine | S0183 | 152.055094 | 220.567 | 151.04941 | C5H5N5O | HMDB00403 | 76900 | 62462 |  | 5392 | 3373-53-3 | 0.000408606 | Nucleic acids and derivatives |
| 1055 | N-Methylanthranilic Acid |  | 152.068942 | 221.863 | 151.16 | C8H9NO2 |  |  |  |  |  |  | 0.001272783 |  |
| 875 | 3-Aminopropanesulphonic Acid |  | 155.046609 | 303.046 |  |  |  |  |  |  |  |  | 0.021983418 | Amino acids and derivatives |
| 1177 | Phenylacetylglycine | S0200 | 158.058614 | 51.343 | 193.07389 | C10H11NO3 | HMDB00821 | 68144 | 27480 | C05598 | 4237 | 500-98-1 | 0.002697618 |  |
| 1179 | L-Citrulline | S0063 | 158.09064 | 303.718 | 175.09569 | C6H13N3O3 | HMDB00904 | 9750 | 16349 | C00327 | 16 | 372-75-8 | 0.001104867 | Amino acids and derivatives |
| 715 | Tyramine | S0124 | 160.07407 | 44.862 | 137.08406 | C8H11NO | HMDB00306 | 5610 | 15760 | C00483 | 60 | 51-67-2 | 0.000438192 | Amino acids and derivatives |
| 1260 | L-Carnitine | S0060 | 162.112136 | 347.489 | 161.10519 | C7H15NO3 | HMDB00062 | 2724480 | 11060 | C00318 | 52 | 541-15-1 | 0.033956773 |  |
| 756 | Butyl lactate | L0276 | 164.126356 | 336.55 | 146.09429 | C7H14O3 | HMDB40254 | 8738 |  |  |  | 138-22-7 | 0.000815644 |  |
| 1057 | 1,2-Benzenedicarboxylic acid |  | 165.020121 | 248.084 | 166.13 | C8H6O4 |  |  |  |  |  |  | 0.090250667 |  |
| 1317 | L-Phenylalanine | S0061 | 166.087569 | 202.038 | 165.07898 | C9H11NO2 | HMDB00159 | 6140 | 17295 | C00079 | 28 | 63-91-2 | 0.003377833 | Amino acids and derivatives |
| 716 | L-Asparagine | S0104 | 169.006269 | 118.576 | 132.05349 | C4H8N2O3 | HMDB00168 | 6267 | 17196 | C00152 | 14 | 70-47-3 | 0.016124296 | Amino acids and derivatives |
| 1378 | Pyridoxine | S0080 | 170.079692 | 130.112 | 169.07389 | C8H11NO3 | HMDB00239 | 1054 | 16709 | C00314 | 5245 | 65-23-6 | 0.00449362 |  |
| 1434 | Gly-Pro |  | 173.087809 | 27.255 | 172.18 | C7H12N2O3 |  |  |  |  |  |  | 0.001273804 | Amino acids and derivatives |
| 1470 | L-Arginine | S0062 | 175.119137 | 520.651 | 174.11168 | C6H14N4O2 | HMDB00517 | 6322 | 16467 | C00062 | 5502 | 74-79-3 | 0.10130184 | Amino acids and derivatives |
| 1485 | Hippuric acid, methyl ester |  | 176.069223 | 186.8725 | 193.2 | C10H11NO3 |  |  |  |  |  |  | 0.001479883 |  |
| 1312 | D-Mannose | S0138 | 179.056263 | 383.2245 | 180.06339 | C6H12O6 | HMDB00169 | 18950 | 4208 | C00159 |  | 3458-28-4 | 0.002463838 | Carbohydrates and derivatives |
| 836 | myo-Inositol | S0024 | 179.056381 | 296.757 | 180.06339 | C6H12O6 | HMDB00211 |  | 17268 | C00137 | 5221 | 87-89-8 | 0.00092475 | Lipids and derivatives |
| 859 | D-Threitol | S0351 | 181.072213 | 291.954 | 122.05791 | C4H10O4 | HMDB04136 | 169019 | 48300 | C16884 |  | 2418-52-2 | 0.004199081 | Carbohydrates and derivatives |
| 1349 | L-Iditol | L0396 | 181.072216 | 291.986 | 182.07904 | C6H14O6 | HMDB11632 | 5460044 | 18202 | C01507 |  | 488-45-9 | 0.004199081 | Carbohydrates and derivatives |
| 1577 | L-Tyrosine | S0064 | 182.079718 | 296.1835 | 181.07389 | C9H11NO3 | HMDB00158 | 6057 | 17895 | C00082 | 34 | 60-18-4 | 0.000815077 | Amino acids and derivatives |
| 1609 | 4-Pyridoxic acid | L0261 | 184.05904 | 42.844 | 183.05316 | C8H9NO4 | HMDB00017 | 6723 | 17405 | C00847 | 239 | 82-82-6 | 0.008162165 |  |
| 1611 | Phosphorylcholine | L0132 | 184.072773 | 497.869 | 183.06605 | C5H14NO4P | HMDB01565 | 1014 | 18132 | C00588 | 6326 | 107-73-3 (72556-74-2) | 0.014362179 | Lipids and derivatives |
| 1628 | 2-Methyl-3-hydroxybutyric acid | L0197 | 185.016432 | 235.662 | 118.063 | C5H10O3 | HMDB00354 | 160471 | 37051 |  | 3786 | 473-86-9 | 0.001511834 |  |
| 1426 | D-Quinovose | n00510 | 185.04564 | 108.908 | 164.06847 | C6H12O5 |  |  |  | C02522 |  | 2103284 | 0.001004084 | Carbohydrates and derivatives |
| 1470 | Azelaic acid | S0199 | 187.097894 | 323.206 | 188.10486 | C9H16O4 | HMDB00784 | 2266 | 48131 | C08261 | 5750 | 123-99-9 | 0.002527485 |  |
| 1711 | L-Glutamate | S0056 | 189.085868 | 296.31 | 147.05316 | C5H9NO4 | HMDB00148 | 33032 | 16015 | C00025 | 5174 | 56-86-0 | 0.000263472 | Amino acids and derivatives |
| 1058 | N-Acetylglutamine | S0265 | 189.085875 | 296.571 | 188.07971 | C7H12N2O4 | HMDB06029 | 25561 |  |  |  | 2490-97-3 | 0.000263472 | Amino acids and derivatives |
| 1718 | N6-Acetyl-L-lysine | L0011 | 189.121885 | 342.69 | 188.11609 | C8H16N2O3 | HMDB00206 | 92832 | 17752 | C02727 | 5216 | 692-04-6 | 0.000630982 | Amino acids and derivatives |
| 975 | Citrate | S0027 | 191.019993 | 509.223 | 192.02701 | C6H8O7 | HMDB00094 | 311 | 30769 | C00158 | 124 | 77-92-9 | 0.062011618 |  |
| 1538 | Quinate | L0443 | 191.056767 | 346.679 | 192.06339 | C7H12O6 | HMDB03072 |  | 17521 | C00296 | 3329 | 77-95-2 | 0.001927105 | Amino acids and derivatives |
| 1087 | Quinaldic acid | n00570 | 191.080092 | 41.495 | 173.04768 | C10H7NO2 | HMDB00842 | 7124 | 18386 | C06325 | 5805 | 34249 | 0.000800555 |  |
| 1797 | Vanillin | L0436 | 194.079874 | 186.993 | 152.04735 | C8H8O3 | HMDB12308 | 1183 | 18346 | C00755 |  | 121-33-5 | 0.001025375 |  |
| 1805 | Agmatine | S0191 | 194.137459 | 270.9145 | 130.12185 | C5H14N4 | HMDB01432 | 199 | 17431 | C00179 | 3523 | 306-60-5 (2482-00-0) | 0.001470695 | Amino acids and derivatives |
| 1850 | Pro-Val |  | 197.127154 | 49.24 | 214.26 | C10H18N2O3 |  |  |  |  |  |  | 0.00163417 | Amino acids and derivatives |
| 1705 | L-homocysteic acid | S0114 | 199.043105 | 46.657 | 183.02015 | C4H9NO5S | HMDB02205 | 177491 | 488363 | C16511 | 6545 | 14857-77-3 | 0.004602791 | Amino acids and derivatives |
| 1896 | p-CHLOROPHENYLALANINE |  | 200.045988 | 320.093 | 199.63 | C9H10ClNO2 |  |  |  |  |  |  | 0.003149967 | Amino acids and derivatives |
| 1743 | Dicamba |  | 200.951726 | 180.015 | 221.04 | C8H6Cl2O3 |  |  |  |  |  |  | 0.048018081 |  |
| 1759 | 3-Phosphoserine | S0065 | 201.026372 | 381.916 | 185.00893 | C3H8NO6P | HMDB00272 | 106 | 37712 | C01005 | 6338 | 407-41-0 | 0.051826736 | Amino acids and derivatives |
| 1761 | 1,7-Dimethylxanthine | S0370 | 201.038349 | 31.303 | 180.06473 | C7H8N4O2 | HMDB01860 | 4687 | 25858 | C13747 | 1457 | 611-59-6 | 0.136866682 | Nucleic acids and derivatives |
| 1946 | 3-Hydroxybenzoate | S0388 | 202.044414 | 235.0425 | 138.0317 | C7H6O3 | HMDB02466 | 7420 | 30764 | C00587 | 6690 | 36320 | 0.290392619 |  |
| 1994 | Acetylcarnitine | L0133 | 204.122115 | 298.7825 | 203.11576 | C9H17NO4 | HMDB00201 | 1 |  | C02571 | 5213 | 870-77-9 (2504-11-2) | 0.006801222 |  |
| 1817 | Pantothenol | S0314 | 204.124701 | 73.083 | 205.13141 | C9H19NO4 | HMDB04231 | 4678 | 895821 | C05944 | 7032 | 81-13-0 | 0.00826222 |  |
| 1861 | Clopidol |  | 207.012904 | 32.888 | 192.04 | C7H7Cl2NO |  |  |  |  |  |  | 0.037547567 |  |
| 1863 | Malonic acid | S0204 | 207.013049 | 104.9615 | 104.01096 | C3H4O4 | HMDB00691 | 867 | 30794 | C00383 | 3237 | 141-82-2 | 0.017440132 |  |
| 2052 | Monomethyl glutaric acid | S0247 | 210.077938 | 70.662 | 146.05791 | C6H10O4 | HMDB00858 | 73917 | 1E+06 |  | 5821 | 1501-27-5 | 0.00120735 |  |
| 2066 | L-Serine | S0090 | 211.096955 | 65.329 | 105.04259 | C3H7NO3 | HMDB00187 | 5951 | 17115 | C00065 | 5203 | 56-45-1 | 0.011045432 | Amino acids and derivatives |
| 2070 | Nabumetone |  | 211.108397 | 32.633 | 228.29 | C15H16O2 |  |  |  |  |  |  | 0.002809535 |  |
| 2006 | sn-Glycerol 3-phosphoethanolamine | S0362 | 214.049692 | 383.839 | 215.05588 | C5H14NO6P | HMDB59660 | 5459861 | 16929 |  |  |  | 0.002193792 | Lipids and derivatives |
| 2025 | 2-keto-D-Gluconic acid |  | 215.019321 | 57.098 | 426.34 | C12H18CaO14 |  |  |  |  |  |  | 0.048969478 | Carbohydrates and derivatives |
| 2182 | N-.alpha.-Acetyl-L-arginine |  | 217.127813 | 360.4435 | 216.2376 | C8H16N4O3 |  |  |  |  |  |  | 0.000973626 | Amino acids and derivatives |
| 1397 | Dodecanoic acid | S0184 | 218.210228 | 93.426 | 200.17763 | C12H24O2 | HMDB00638 | 3893 | 30805 | C02679 | 5611 | 143-07-7 | 0.003678913 | Lipids and derivatives |
| 2232 | Ketorolac |  | 220.08008 | 33.44 | 255.27 | C15H13NO3 |  |  |  |  |  |  | 0.000705606 |  |
| 2234 | Isovalerylglycine | S0195 | 220.116735 | 257.3925 | 159.08954 | C7H13NO3 | HMDB00678 | 546304 |  |  | 5647 | 16284-60-9 | 0.000940131 | Amino acids and derivatives |
| 2269 | Acetylcholine | L0265 | 222.028049 | 236.3065 | 145.11028 | C7H15NO2 | HMDB00895 | 187 | 15355 | C01996 | 57 | 60-31-1 | 0.00235001 | Lipids and derivatives |
| 2285 | Glycerol | S0302 | 223.062733 | 32.963 | 92.04735 | C3H8O3 | HMDB00131 | 753 | 17522 | C00116 | 105 | 56-81-5 | 0.005479251 | Lipids and derivatives |
| 2265 | 3-Indolepropionic acid | n00704 | 226.028236 | 165.1445 | 189.07898 | C11H11NO2 | HMDB02302 |  |  |  |  | 830-96-6 | 0.007947868 |  |
| 2287 | Marmesin |  | 227.075267 | 31.468 |  | C14H14O4 |  |  |  |  |  |  | 0.424983296 |  |
| 2299 | Myristic acid | S0207 | 227.201562 | 18.555 | 228.20893 | C14H28O2 | HMDB00806 | 11005 | 28875 | C06424 | 196 | 544-63-8 | 0.046228089 | Lipids and derivatives |
| 1537 | (R)-mevalonic acid 5-Phosphate | L0453 | 229.140849 | 37.1645 | 228.137 | C6H13O7P | HMDB01343 | 439400 | 17436 | C01107 | 6177 |  | 0.001396012 |  |
| 2381 | Pectin (Galacturonic acid) | S0251 | 230.990958 | 31.633 | 194.04265 | C6H10O7 | HMDB03402 | 441476 | 47954 | C08348 | 6916 | 9000-69-5 | 0.044716331 | Carbohydrates and derivatives |
| 2388 | Maleic acid | S0004 | 231.019771 | 111.524 | 116.01096 | C4H4O4 | HMDB00176 | 444266 | 18300 | C01384 | 4198 | 110-16-7 | 0.007379976 |  |
| 2453 | Lys-Cys |  | 232.116864 | 197.2225 |  |  |  |  |  |  |  |  | 0.002387797 | Amino acids and derivatives |
| 2552 | Alizarin | S0274 | 239.039813 | 33.503 | 240.04226 | C14H8O4 |  |  |  | C01474 |  | 72-48-0 | 0.029685758 |  |
| 2555 | D-Fructose | S0236 | 239.078138 | 383.137 | 180.06339 | C6H12O6 | HMDB00660 | 439709 | 28645 | C00095 | 135 | 57-48-7 | 0.002463838 | Carbohydrates and derivatives |
| 1666 | 4-Methoxycinnamic acid | S0234 | 239.094443 | 27.255 | 178.063 | C10H10O3 | HMDB02040 | 699414 | 260249 |  | 6453 | 830-09-1 | 0.000809899 | Amino acids and derivatives |
| 2616 | Fenoprofen |  | 241.084297 | 99.433 | 242.27 | C15H14O3 |  |  |  |  |  |  | 0.006714764 |  |
| 2646 | N-Acetyl-D-galactosamine | L0466 | 242.177642 | 33.27 | 221.2078 | C8H15NO6 | HMDB00212 | 84265 | 40356 | C01132 | 5222 |  | 0.014183528 | Carbohydrates and derivatives |
| 1741 | Lumichrome | S0293 | 243.086447 | 62.409 | 242.08038 | C12H10N4O2 |  |  |  | C01727 |  | 1086-80-2 | 0.000529815 |  |
| 2689 | 2-Deoxy-D-glucose 6-phosphate |  | 244.037328 | 33.8725 |  | C6H13O8P |  |  |  |  |  |  | 0.013546431 | Carbohydrates and derivatives |
| 2696 | Arg-Ser |  | 244.139354 | 401.9715 |  |  |  |  |  |  |  |  | 0.000326007 | Amino acids and derivatives |
| 2747 | Arg-Ala |  | 246.15485 | 412.4295 | 305.33 | C11H23N5O5 |  |  |  |  |  |  | 0.000492783 | Amino acids and derivatives |
| 2751 | Lys-Val |  | 246.180076 | 545.829 |  | C11H23N3O3 |  |  |  |  |  |  | 0.000705529 | Amino acids and derivatives |
| 2775 | Pro-Met |  | 247.114425 | 84.066 |  |  |  |  |  |  |  |  | 0.001575587 | Amino acids and derivatives |
| 2870 | His-Pro |  | 252.12194 | 46.4785 |  | C11H16N4O3 |  |  |  |  |  |  | 0.00524812 | Amino acids and derivatives |
| 2872 | Acetyl Tyrosine Ethyl Ester |  | 252.128825 | 26.592 | 269.29 | C13H19NO5 |  |  |  |  |  |  | 0.001498291 | Amino acids and derivatives |
| 1902 | D-Ribulose 5-phosphate | L0289 | 253.013459 | 366.768 | 230.01916 | C5H11O8P | HMDB00618/HMDB01548 | 53477706 | 17797 | C00199 | 5591 | 4151-19-3 (4300-28-1) | 0.000325169 | Carbohydrates and derivatives |
| 2829 | cis-9-Palmitoleic acid | L0166 | 253.2182 | 2.631 | 254.22458 | C16H30O2 | HMDB03229 | 445638 | 28716 | C08362 | 188 | 373-49-9 | 0.006510487 | Lipids and derivatives |
| 2865 | Barbituric acid | S0381 | 255.033613 | 33.724 | 128.02219 | C4H4N2O3 | HMDB41833 | 6211 | 16294 | C00813 |  | 67-52-7 | 0.049880799 |  |
| 2912 | GW 9662 |  | 257.014938 | 31.177 | 276.68 | C13H9ClN2O3 |  |  |  |  |  |  | 0.049880799 |  |
| 2913 | Alternariol |  | 257.050261 | 32.888 | 258.23 | C14H10O5 |  |  |  |  |  |  | 0.037402621 |  |
| 3008 | Glycerophosphocholine | S0106 | 258.108928 | 381.3055 | 258.11065 | C8H21NO6P+ | HMDB00086 | 71920 | 16870 | C00670 | 370 | 28319-77-9 | 0.002173647 | Lipids and derivatives |
| 2947 | D-Erythrose 4-phosphate | L0290 | 259.023796 | 441.0865 | 200.00859 | C4H9O7P | HMDB01321 | 122357 | 48153 | C00279 | 6158 | 585-18-2 (103302-15-4) | 0.048775592 | Carbohydrates and derivatives |
| 2949 | Fluorouracil |  | 259.029967 | 35.328 | 130.08 | C4H3FN2O2 |  |  |  |  |  |  | 0.015007236 | Nucleic acids and derivatives |
| 2024 | alpha-D-Glucose 1-phosphate | L0283 | 261.036104 | 441.73 | 260.02972 | C6H13O9P | HMDB01586 | 439165 | 16077 | C00103 | 6331 | 56401-20-8(59-56-3) | 0.025097345 | Carbohydrates and derivatives |
| 3076 | D-Biotin | L0014 | 262.127785 | 382.6745 | 244.08817 | C10H16N2O3S | HMDB00030 | 171548 | 15956 | C00120 | 243 | 58-85-5 | 0.000899971 |  |
| 3131 | Thiamine | S0085 | 265.110706 | 362.01 | 265.11231 | C12H17N4OS | HMDB00235 | 1130 | 18385 | C00378 | 5242 | 59-43-8 (67-03-8) | 0.00148736 |  |
| 3103 | Inosine | S0032 | 267.074572 | 212.626 | 268.08077 | C10H12N4O5 | HMDB00195 | 6021 | 17596 | C00294 | 84 | 58-63-9 | 0.008368626 | Nucleic acids and derivatives |
| 3182 | Ibuprofen | n00816 | 267.158353 | 37.1305 | 206.13068 | C13H18O2 | HMDB01925 | 3672 | 5855 | C01588 | 572 | 15687-27-1 | 0.003094803 | Amino acids and derivatives |
| 3197 | Adenosine | L0016 | 268.103464 | 167.7785 | 267.09675 | C10H13N5O4 | HMDB00050 | 60961 | 16335 | C00212 | 86 | 58-61-7 | 0.008166426 | Nucleic acids and derivatives |
| 3157 | Heptadecanoic acid | S0367 | 269.249796 | 9.9525 | 270.25588 | C17H34O2 | HMDB02259 | 10465 | 32365 |  | 6578 | 506-12-7 | 0.000760452 | Lipids and derivatives |
| 3270 | Phenylpropionylglycine | L0200 | 271.110081 | 187.665 | 207.08954 | C11H13NO3 | HMDB00860 | 152323 | 266653 |  | 5823 | 56613-60-6 | 0.000635076 | Amino acids and derivatives |
| 2117 | 16-Hydroxypalmitic acid | S0320 | 271.229379 | 67.374 | 272.23514 | C16H32O3 | HMDB06294 | 7058075 | 55329 | C18218 |  | 506-13-8 | 0.012233164 | Lipids and derivatives |
| 3357 | Palmitic acid | S0135 | 274.273996 | 79.1015 | 256.24023 | C16H32O2 | HMDB00220 | 985 | 15756 | C00249 | 187 | 21096 | 0.32737572 | Lipids and derivatives |
| 3429 | L-Saccharopine | L0155 | 277.138416 | 431.1275 | 276.13214 | C11H20N2O6 | HMDB00279 | 160556 | 16927 | C00449 | 383 | ?997-68-2 | 0.000669617 | Amino acids and derivatives |
| 3313 | Phthalic acid Mono-2-ethylhexyl Ester |  | 277.146201 | 13.531 | 278.34 | C16H22O4 |  |  |  |  |  |  | 0.003503481 |  |
| 3345 | Thymidine | S0030 | 279.040466 | 158.9085 | 242.09027 | C10H14N2O5 | HMDB00273 | 5789 | 17748 | C00214 | 3375 | 50-89-5 | 0.131132398 | Nucleic acids and derivatives |
| 3397 | Oleic acid | L0123 | 281.24957 | 3.041 | 282.25588 | C18H34O2 | HMDB00207 | 445639 | 16196 | C00712 | 190 | 112-80-1 | 0.131132398 | Lipids and derivatives |
| 3548 | 2'-O-methyladenosine | L0234 | 282.118526 | 103.6715 | 281.11241 | C11H15N5O4 | HMDB04326 | 317398 | 119928 |  |  | 2140-79-6 | 0.001331879 | Nucleic acids and derivatives |
| 3554 | Ethyl 3-hydroxybutyrate |  | 282.190159 | 261.371 | 132.16 | C6H12O3 |  |  |  |  |  |  | 0.000618243 |  |
| 3614 | Embelin |  | 293.182812 | 25.074 | 294.39 | C17H26O4 |  |  |  |  |  |  | 0.274121225 |  |
| 3652 | 9(S)-HODE |  | 295.228613 | 58.603 | 296.44 | C18H32O3 |  |  |  |  |  |  | 0.032102121 | Lipids and derivatives |
| 3703 | Nname,cis-9,10-Epoxystearic acid |  | 297.24503 | 50.6555 | 298.46 | C18H34O3 |  |  |  |  |  |  | 0.053091162 | Lipids and derivatives |
| 3860 | S-Methyl-5'-thioadenosine | L0036 | 298.095791 | 100.9985 | 297.08956 | C11H15N5O3S | HMDB01173 | 439176 | 17509 | C00170 | 3425 | 2457-80-9 | 0.003620913 | Nucleic acids and derivatives |
| 3762 | Palmitaldehyde | L0346 | 299.260683 | 56.8775 | 240.24532 | C16H32O | HMDB01551 | 984 | 17600 | C00517 | 6317 | 629-80-1 | 0.008400302 |  |
| 2574 | (Z)-6-Octadecenoic acid |  | 300.288955 | 169.155 | 282.46 | C18H34O2 |  |  |  |  |  |  | 0.000130436 | Lipids and derivatives |
| 3816 | Oxazepam |  | 302.067624 | 167.7945 | 286.71 | C15H11ClN2O2 |  |  |  |  |  |  | 0.012489914 |  |
| 3959 | Sphinganine | L0159 | 302.303739 | 133.4335 | 301.29808 | C18H39NO2 | HMDB00269 | 91486 | 16566 | C00836 | 5268 | 764-22-7 | 0.000511065 | Lipids and derivatives |
| 3958 | Stearic acid | S0233 | 302.304399 | 74.592 | 284.27153 | C18H36O2 | HMDB00827 | 5281 | 28842 | C01530 | 189 | 21128 | 4.259769966 | Lipids and derivatives |
| 3833 | Arachidonic Acid (peroxide free) | L0145 | 303.23459 | 38.408 | 304.24023 | C20H32O2 | HMDB01043 | 444899 | 15843 | C00219 | 193 | 506-32-1 | 0.021428304 | Lipids and derivatives |
| 3880 | Dihomo-gamma-Linolenic Acid |  | 305.249698 | 77.819 | 306.48 | C20H34O2 |  |  |  |  |  |  | 0.016878173 | Lipids and derivatives |
| 4119 | Nadolol | n01434 | 310.200533 | 483.518 | 309.19401 | C17H27NO4 | HMDB15334 | 39147 | 127570 |  |  | 42200-33-9 | 0.004110521 |  |
| 3983 | Phytanic acid | L0401 | 311.29742 | 38.418 | 312.30283 | C20H40O2 | HMDB00801 | 26840 | 16285 | C01607 | 5765 | 14721-66-5 | 0.001818473 | Lipids and derivatives |
| 4285 | Phytosphingosine | L0377 | 318.29941 | 153.7685 | 317.29299 | C18H39NO3 | HMDB04610 | 122121 | 46961 | C12144 | 7066 | 554-62-1 | 0.000288272 | Lipids and derivatives |
| 4346 | Erucic acid | S0288 | 321.308298 | 80.722 | 338.31848 | C22H42O2 | HMDB02068 | 5281116 | 28792 | C08316 | 6470 | 112-86-7 | 0.007726598 | Amino acids and derivatives |
| 4206 | Hexadecanedioic acid |  | 323.170307 | 47.958 | 286.41 | C16H30O4 |  |  |  |  |  |  | 0.025677671 | Lipids and derivatives |
| 4444 | D-Glucose 6-phosphate | L0272 | 326.984163 | 443.066 | 260.02972 | C6H13O9P | HMDB01401 | 5958 | 4170 | C00092 | 145 | 56-73-5 | 0.013546431 | Carbohydrates and derivatives |
| 4514 | 9-Decen-1-ol |  | 330.335985 | 38.1825 | 156.27 | C10H20O |  |  |  |  |  |  | 0.000651511 | Lipids and derivatives |
| 4590 | Heptanoic acid, ethyl ester |  | 334.294018 | 41.082 | 158.24 | C9H18O2 |  |  |  |  |  |  | 0.000893771 |  |
| 4600 | beta-Nicotinamide D-ribonucleotide | L0168 | 335.063031 | 477.6765 | 335.06443 | C11H16N2O8P+ | HMDB59645 | 14181 | 14648 | C00455 |  | 1094-61-7 | 0.000943994 | Nucleic acids and derivatives |
| 4657 | (+)-Methamphetamine |  | 337.200506 | 34.4955 | 149.23 | C10H15N |  |  |  |  |  |  | 0.006131632 |  |
| 4679 | Erucamide | L0202 | 338.343074 | 34.201 | 337.33446 | C22H43NO |  |  |  |  |  | 112-84-5 | 0.006763227 | Amino acids and derivatives |
| 4509 | Norethindrone Acetate |  | 339.201939 | 46.7295 | 340.46 | C22H28O3 |  |  |  |  |  |  | 0.363239033 | Lipids and derivatives |
| 4554 | Sucrose | S0033 | 341.110963 | 389.377 | 342.11622 | C12H22O11 | HMDB00258 | 5988 | 17992 | C00089 | 137 | 57-50-1 | 0.012134866 | Carbohydrates and derivatives |
| 4799 | Stearamide | S0286 | 344.315728 | 54.83 | 283.28751 | C18H37NO | HMDB34146 | 31292 |  | C13846 |  | 124-26-5 | 0.000402804 | Lipids and derivatives |
| 4822 | 3',5'-Cyclic guanosine monophosphate | L0381 | 346.053781 | 307.1345 | 345.04744 | C10H12N5O7P | HMDB01314 |  |  | C00942 |  | 7665-99-8 | 0.001031192 | Nucleic acids and derivatives |
| 4638 | Adenosine monophosphate (AMP) | L0018 | 346.057465 | 421.8835 | 347.06309 | C10H14N5O7P | HMDB00045 | 6083 | 16027 | C00020 | 5111 | 61-19-8 (149022-20-8) | 0.005920548 | Nucleic acids and derivatives |
| 4848 | 3'-O-methyladenosine | L0251 | 348.069353 | 475.1595 | 281.11241 | C11H15N5O4 |  | 82530 |  |  |  | 10300-22-8 | 0.001092939 | Nucleic acids and derivatives |
| 4847 | Adenosine 3'-monophosphate | L0392 | 348.069616 | 421.5825 | 347.06309 | C10H14N5O7P | HMDB03540 | 41211 | 28931 | C01367 |  | 84-21-9 | 0.003007118 | Nucleic acids and derivatives |
| 3131 | Tetrahydrocorticosterone |  | 349.246447 | 24.4615 | 350.49 | C21H34O4 |  |  |  |  |  |  | 0.007759069 | Lipids and derivatives |
| 5055 | Pentadecanoic Acid | S0364 | 357.093381 | 234.446 | 242.22458 | C15H30O2 | HMDB00826 | 13849 |  | C16537 | 5789 | 1002-84-2 | 0.066265723 | Lipids and derivatives |
| 5098 | Behenic acid | S0350 | 358.367322 | 37.7205 | 340.33413 | C22H44O2 | HMDB00944 | 8215 | 28941 | C08281 | 260 | 112-85-6 | 0.006977793 | Lipids and derivatives |
| 5141 | Isomaltose | S0341 | 360.148755 | 388.4445 | 342.11622 | C12H22O11 | HMDB02923 | 439193 | 28189 | C00252 | 412 | 499-40-1 | 0.000734723 | Carbohydrates and derivatives |
| 4973 | Tetracosanoic acid | L0302 | 367.360191 | 38.081 | 368.36543 | C24H48O2 | HMDB02003 | 11197 | 28866 | C08320 | 6427 | 557-59-5 | 0.014023728 | Lipids and derivatives |
| 5429 | Norharmane |  | 375.097446 | 350.6805 | 168.19 | C11H8N2 |  |  |  |  |  |  | 0.001376961 |  |
| 5564 | Pantoprazole |  | 383.072447 | 143.758 | 383.37 | C16H15F2N3O4S |  |  |  |  |  |  | 0.0004383 |  |
| 5347 | 5(S)-HpETE |  | 395.245539 | 36.943 | 336.47 | C20H32O4 |  |  |  |  |  |  | 0.008765035 | Lipids and derivatives |
| 5349 | Hexacosanoic acid | L0300 | 395.391906 | 37.818 | 396.39673 | C26H52O2 | HMDB02356 | 10469 | 31009 |  | 6642 | 506-46-7 | 0.00668207 | Lipids and derivatives |
| 3673 | Trehalose | S0313 | 401.132467 | 389.142 | 342.11622 | C12H22O11 | HMDB00975 | 7427 | 16551 | C01083 | 5913 | 99-20-7 | 0.010742397 | Carbohydrates and derivatives |
| 6278 | D-Ribulose 1,5-bisphosphate | L0394 | 424.8638 | 381.3975 | 309.98549 | C5H12O11P2 | HMDB35796 | 151261 | 17173 | C01182 | 5594 | 14689-84-0 | 0.002812015 | Carbohydrates and derivatives |
| 6557 | Tangeritin |  | 439.084493 | 234.7575 | 372.37 | C20H20O7 |  |  |  |  |  |  | 0.038050771 |  |
| 6181 | Fludrocortisone acetate |  | 459.158713 | 25.333 | 422.49 | C23H31FO6 |  |  |  |  |  |  | 0.004835142 | Lipids and derivatives |
| 5154 | 1-Palmitoyl-sn-glycero-3-phosphocholine |  | 496.338752 | 188.515 | 495.63 | C24H50NO7P |  |  |  |  |  |  | 0.000160537 | Lipids and derivatives |
| 9447 | UDP-N-acetylglucosamine | L0303 | 608.088514 | 407.4495 | 607.08158 | C17H27N3O17P2 | HMDB00290 | 445675 | 16264 | C00043 | 5281 | 528-04-1 (91183-98-1) | 0.000318415 | Carbohydrates and derivatives |
| 7468 | Glutathione disulfide | L0008 | 611.149846 | 477.2185 | 612.15197 | C20H32N6O12S2 | HMDB03337 | 975 | 17858 | C00127 | 6893 | 27025-41-8 | 0.003502439 | Amino acids and derivatives |
| 10333 | Nicotinamide adenine dinucleotide (NAD) | L0009 | 664.116479 | 419.632 | 663.10913 | C21H27N7O14P2 | HMDB00902 | 5893 | 15846 | C00003 | 5858 | 53-84-9 | 0.000454055 | Nucleic acids and derivatives |
| 11567 | PC(16:0/16:0) | S0151 | 756.555267 | 142.6985 | 733.56216 | C40H80NO8P | HMDB00564 | 452110 |  | C00157 | 5548 | 63-89-8 | 0.000990609 | Lipids and derivatives |
| 11590 | Thioetheramide-PC |  | 758.570021 | 142.965 | 789.27 | C45H93N2O4PS |  |  |  |  |  |  | 0.001579628 |  |
| 11952 | 1,2-dioleoyl-sn-glycero-3-phosphatidylcholine |  | 786.601458 | 141.595 | 786.11 | C44H84NO8P |  |  |  |  |  |  | 0.000836429 | Lipids and derivatives |
| 12197 | 1-Stearoyl-2-oleoyl-sn-glycerol 3-phosphocholine (SOPC) | L0411 | 810.597798 | 37.629 | 787.60911 | C44H86NO8P | HMDB08038 |  |  |  |  |  | 0.000216746 | Lipids and derivatives |
|  |  |  |  |  |  |  |  |  |  |  |  |  |  |  |
| ST-Metabolite Mapping | | | | | | | | | | | | | | |
| 25 | 2-Amino-2-methyl-1,3-propanediol |  | 70.06446367 | 304.3545 | 105.14 | C4H11NO2 |  |  |  |  |  |  | 0.012106918 |  |
| 99 | L-Threonine | S0045 | 84.0435499 | 365.614 | 119.05824 | C4H9NO3 | HMDB00167 | 6288 | 16857 | C00188 | 32 | 72-19-5 | 0.012447263 | Amino acids and derivatives |
| 127 | Diacetyl | L0440 | 87.04305906 | 16.126 | 86.03678 | C4H6O2 | HMDB03407 | 650 | 16583 | C00741 | 6921 | 431-03-8 | 0.007985932 |  |
| 135 | DL-lactate | S0075 | 89.02373624 | 16.311 | 90.03169 | C3H6O3 | HMDB01311/HMDB00190 | 9378 | 17385 |  | 5723 | 50-21-5 | 0.06526192 |  |
| 98 | Sarcosine | S0074 | 90.05395771 | 338.996 | 89.04768 | C3H7NO2 | HMDB00271 | 1088 | 15611 | C00213 | 51 | 107-97-1 | 0.005618951 | Lipids and derivatives |
| 151 | L-Alanine | S0087 | 90.0539995 | 338.7655 | 89.04768 | C3H7NO2 | HMDB00161 | 5950 | 16977 | C00041 |  | 56-41-7 | 0.005618951 | Amino acids and derivatives |
| 221 | DL-2,4-Diaminobutyric acid |  | 101.069409 | 365.541 | 118.1344 | C4H10N2O2 |  |  |  |  |  |  | 0.000793915 |  |
| 168 | L-2-Aminobutyric acid |  | 104.0688588 | 326.602 |  |  |  |  |  |  |  |  | 0.001040131 | Carbohydrates and derivatives |
| 270 | Choline | S0110 | 104.1056933 | 285.9665 | 104.10754 | C5H14NO | HMDB00097 | 305 | 15354 | C00114 | 56 | 62-49-7 (67-48-1) | 0.026942526 | Lipids and derivatives |
| 286 | Diethanolamine | n00109 | 106.0845297 | 321.3125 | 105.07898 | C4H11NO2 | HMDB04437 | 8113 | 28123 | C06772 | 3239 | 111-42-2 | 0.001355709 | Lipids and derivatives |
| 336 | Benzylazanium |  | 108.0789873 | 47.257 | 108.161 | C7H10N+ |  |  |  |  |  |  | 0.004199956 |  |
| 274 | Uracil | S0002 | 111.0198225 | 214.2865 | 112.02728 | C4H4N2O2 | HMDB00300 | 1174 | 17568 | C00106 | 258 | 66-22-8 | 0.004598021 | Nucleic acids and derivatives |
| 383 | Creatinine | S0042 | 114.0645024 | 338.7665 | 113.05891 | C4H7N3O | HMDB00562 | 588 | 16737 | C00791 | 8 | 60-27-5 | 0.001100643 | Amino acids and derivatives |
| 385 | epsilon-Caprolactam |  | 114.0900903 | 47.324 | 113.16 | C6H11NO |  |  |  |  |  |  | 0.028761197 |  |
| 239 | D-Proline | S0043 | 116.0690723 | 304.477 | 115.06333 | C5H9NO2 | HMDB03411 | 8988 | 16313 | C00763 |  | 344-25-2 | 0.020431695 |  |
| 412 | L-Proline | S0088 | 116.0691282 | 304.3545 | 115.06333 | C5H9NO2 | HMDB00162 | 145742 | 17203 | C00148 | 29 | 147-85-3 | 0.020431695 | Amino acids and derivatives |
| 360 | Succinate | S0006 | 117.0192961 | 365.5965 | 118.02661 | C4H6O4 | HMDB00254 | 1110 | 15741 | C00042 | 114 | 110-15-6 | 0.018692052 |  |
| 444 | Betaine | L0010 | 118.0846454 | 387.9295 | 117.07898 | C5H11NO2 | HMDB00043 | 247 | 17750 | C00719 | 287 | 107-43-7 (590-46-5) | 0.027258551 |  |
| 463 | Arecoline | n00443 | 120.0791632 | 253.6455 | 155.09463 | C8H13NO2 | HMDB30353 | 2230 | 101022 | C10129 |  | 63-75-2 | 0.003366625 |  |
| 258 | Benzoic acid |  | 121.0293709 | 247.746 | 122.12 | C7H6O2 |  |  |  |  |  |  | 0.013455562 |  |
| 485 | N,N-Dimethylaniline | n00178 | 122.0947543 | 46.547 | 121.08915 | C8H11N | HMDB01020 | 949 | 16269 | C02846 | 5949 | 121-69-7 | 0.001621638 |  |
| 491 | Nicotinamide | S0048 | 123.0539251 | 63.0025 | 122.04801 | C6H6N2O | HMDB01406 | 936 | 17154 | C00153 | 1497 | 98-92-0 | 0.013530503 | Nucleic acids and derivatives |
| 504 | Picolinic acid | n00193 | 124.0374415 | 200.3025 | 123.03203 | C6H5NO2 | HMDB02243 | 1018 | 28747 | C10164 |  | 98-98-6 | 0.000924715 |  |
| 436 | Taurine | S0009 | 125.0160026 | 36.0085 | 125.01467 | C2H7NO3S | HMDB00251 | 1123 | 15891 | C00245 | 31 | 107-35-7 | 0.095346951 | Amino acids and derivatives |
| 481 | Barbituric acid | S0381 | 127.0130157 | 36.112 | 128.02219 | C4H4N2O3 | HMDB41833 | 6211 | 16294 | C00813 |  | 67-52-7 | 0.03054563 |  |
| 542 | 4-Guanidinobutyric acid | L0158 | 128.0804427 | 240.601 | 145.08513 | C5H11N3O2 | HMDB03464 | 500 | 15728 | C01035 | 6938 | 463-00-3 | 0.001526382 |  |
| 555 | Imidazole | S0037 | 129.0643641 | 352.474 | 68.03745 | C3H4N2 | HMDB01525 | 795 | 16069 | C01589 | 4190 | 288-32-4 | 0.002660848 |  |
| 351 | 3-Furancarboxylic acid |  | 130.0484412 | 386.7395 | 112.08 | C5H4O3 |  |  |  |  |  |  | 0.008729742 |  |
| 565 | Pyroglutamic acid |  | 130.0484807 | 385.733 | 129.11 | C5H7NO3 |  |  |  |  |  |  | 0.008729742 | Amino acids and derivatives |
| 566 | L-Glutamate | S0056 | 130.0493057 | 365.519 | 147.05316 | C5H9NO4 | HMDB00148 | 33032 | 16015 | C00025 | 5174 | 56-86-0 | 0.050620052 | Amino acids and derivatives |
| 383 | Hydroxyproline | S0051 | 132.0637805 | 359.82 | 131.05824 | C5H9NO3 | HMDB00725 | 5810 | 18095 | C01157 | 257 | 51-35-4 | 0.000584748 | Amino acids and derivatives |
| 603 | N-Acetyl-L-alanine | S0203 | 132.0641305 | 343.466 | 131.05824 | C5H9NO3 | HMDB00766 | 88064 | 40992 |  | 5733 | 97-69-8 | 0.004140003 | Amino acids and derivatives |
| 604 | Creatine | S0047 | 132.0759597 | 338.7655 | 131.06948 | C4H9N3O2 | HMDB00064 | 586 | 16919 | C00300 | 7 | 57-00-1 | 0.074544742 | Amino acids and derivatives |
| 388 | Pyrrolidine | n00006 | 132.1003827 | 61.988 | 71.0735 | C4H9N | HMDB31641 | 31268 | 33135 |  |  | 123-75-1 | 0.000585826 | Amino acids and derivatives |
| 610 | L-Leucine | S0052 | 132.1004637 | 269.96 | 131.09463 | C6H13NO2 | HMDB00687 | 6106 | 15603 | C00123 | 24 | 61-90-5 | 0.004495538 | Amino acids and derivatives |
| 565 | Adenine | S0054 | 134.0473274 | 167.4495 | 135.0545 | C5H5N5 | HMDB00034 | 190 | 16708 | C00147 | 85 | 73-24-5 | 0.014507524 | Nucleic acids and derivatives |
| 640 | Mandelonitrile | S0386 | 134.0583724 | 132.615 | 133.05276 | C8H7NO | HMDB60486 | 9548674 | 18450 | C00561 |  | 532-28-5 | 0.002172362 |  |
| 641 | Methyl acetoacetate |  | 134.079839 | 338.7655 | 116.12 | C5H8O3 |  |  |  |  |  |  | 0.000365807 |  |
| 578 | Hypoxanthine | S0012 | 135.0310603 | 165.8885 | 136.03851 | C5H4N4O | HMDB00157 | 790 | 17368 | C00262 | 83 | 68-94-0 | 0.002656502 | Nucleic acids and derivatives |
| 660 | Dopamine | L0111 | 136.0740528 | 295.935 | 153.07898 | C8H11NO2 | HMDB00073 | 681 | 18243 | C03758 | 64 | 51-61-6 (62-31-7) | 0.000762641 | Amino acids and derivatives |
| 628 | Urocanic acid | S0242 | 137.0351677 | 169.915 | 138.04293 | C6H6N2O2 | HMDB00301 | 736715 | 30817 | C00785 | 298 | 104-98-3 | 0.00337749 | Amino acids and derivatives |
| 452 | Anthranilic acid (Vitamin L1) | S0363 | 138.0533045 | 278.597 | 137.04768 | C7H7NO2 | HMDB01123 | 227 | 30754 | C00108 | 6018 | 118-92-3 | 0.000363886 |  |
| 696 | Trigonelline | L0464 | 138.0534144 | 278.921 | 137.136 | C7H7NO2 | HMDB00875 | 5570 | 18123 | C01004 | 273 |  | 0.000363886 |  |
| 453 | Phenol | S0142 | 139.0107932 | 235.038 | 94.04187 | C6H6O | HMDB00228 | 996 | 15882 | C00146 | 128 | 108-95-2 | 0.001494254 |  |
| 717 | 1-Aminocyclopropanecarboxylic acid | n00070 | 140.0134696 | 234.864 | 101.04768 | C4H7NO2 | HMDB36458 | 133503 |  |  |  | 22059-21-8 | 0.00075132 | Amino acids and derivatives |
| 657 | 2-Oxoadipic acid | L0329 | 141.0165818 | 124.035 | 160.03717 | C6H8O5 | HMDB00225 | 71 | 15753 | C00322 | 5234 | 3184-35-8 | 0.271059565 |  |
| 479 | 1,3-Diaminopropane | S0309 | 141.037882 | 222.774 | 74.0844 | C3H10N2 | HMDB00002 | 428 | 15725 | C00986 | 5081 | 109-76-2 | 0.007787367 |  |
| 789 | 3-(2-Hydroxyethyl)indole |  | 144.0791055 | 80.313 | 161.2 | C10H11NO |  |  |  |  |  |  | 0.003615931 |  |
| 521 | Cyclohexylamine | n00061 | 144.079129 | 46.1125 | 99.1048 | C6H13N | HMDB31404 | 7965 | 15773 | C00571 |  | 108-91-8 | 0.003296367 | Amino acids and derivatives |
| 790 | D-Cycloserine | n00073 | 144.0798978 | 21.317 | 102.04293 | C3H6N2O2 | HMDB14405 | 6234 | 40009 | C08057 |  | 68-41-7 | 0.004172057 | Amino acids and derivatives |
| 723 | L-Glutamine | S0055 | 145.0616619 | 366.7915 | 146.06914 | C5H10N2O3 | HMDB00641 | 5961 | 18050 | C00064 | 5614 | 56-85-9 | 0.039214152 | Amino acids and derivatives |
| 725 | Mesaconic acid | L0375 | 146.0457513 | 379.5565 | 130.02661 | C5H6O4 | HMDB00749 | 638129 | 16600 | C01732 | 4130 | 498-24-8 | 0.004788327 |  |
| 488 | Citraconic acid | n00245 | 146.0458603 | 379.988 | 130.02661 | C5H6O4 | HMDB00634 | 643798 | 17626 | C02226 | 5607 | 498-23-7 | 0.004788327 | Amino acids and derivatives |
| 820 | (3-Carboxypropyl)trimethylammonium cation |  | 146.1158408 | 368.345 |  |  |  |  |  |  |  |  | 0.000710826 |  |
| 842 | Formylanthranilic acid | S0342 | 148.0378392 | 47.715 | 165.04259 | C8H7NO3 | HMDB04089 | 101399 | 36575 | C05653 |  | 3342-77-6 | 0.001223635 |  |
| 582 | L-Methionine | S0057 | 150.0566983 | 281.313 | 149.05105 | C5H11NO2S | HMDB00696 | 6137 | 16643 | C00073 | 5664 | 63-68-3 | 0.000606972 | Amino acids and derivatives |
| 869 | Triethanolamine | n00366 | 150.1108581 | 166.827 | 149.10519 | C6H15NO3 | HMDB32538 | 7618 | 28621 | C06771 |  | 102-71-6 | 0.001327825 | Lipids and derivatives |
| 782 | Ribitol | S0174 | 151.0618016 | 229.561 | 152.06848 | C5H12O5 | HMDB00508 |  | 15963 | C00474 | 316 | 488-81-3 | 0.084943085 | Carbohydrates and derivatives |
| 605 | 2-Hydroxyadenine |  | 152.0551261 | 220.6505 | 151.13 | C5H5N5O |  |  |  |  |  |  | 0.000569057 |  |
| 837 | 3-Hydroxyisovaleric acid | S0198 | 155.0097816 | 214.359 | 118.063 | C5H10O3 | HMDB00754 | 69362 | 37084 |  | 5722 | 625-08-1 | 0.029602529 | Amino acids and derivatives |
| 997 | L-Citrulline | S0063 | 158.0909379 | 302.753 | 175.09569 | C6H13N3O3 | HMDB00904 | 9750 | 16349 | C00327 | 16 | 372-75-8 | 0.000545062 | Amino acids and derivatives |
| 1029 | Tyramine | S0124 | 160.0740537 | 45.461 | 137.08406 | C8H11NO | HMDB00306 | 5610 | 15760 | C00483 | 60 | 51-67-2 | 0.001746943 | Amino acids and derivatives |
| 1059 | L-Carnitine | S0060 | 162.1121161 | 347.6165 | 161.10519 | C7H15NO3 | HMDB00062 | 2724480 | 11060 | C00318 | 52 | 541-15-1 | 0.025662601 |  |
| 971 | L-Phenylalanine | S0061 | 164.0716719 | 254.7185 | 165.07898 | C9H11NO2 | HMDB00159 | 6140 | 17295 | C00079 | 28 | 63-91-2 | 0.006606539 | Amino acids and derivatives |
| 756 | Butyl lactate | L0276 | 164.1263556 | 336.55 | 146.09429 | C7H14O3 | HMDB40254 | 8738 |  |  |  | 138-22-7 | 0.000410079 |  |
| 982 | 1,2-Benzenedicarboxylic acid |  | 165.0195671 | 68.478 | 166.13 | C8H6O4 |  |  |  |  |  |  | 0.01980691 |  |
| 1102 | (R)-3-Hydroxybutyric acid | L0122 | 165.0742368 | 75.451 | 104.04735 | C4H8O3 | HMDB00011 | 92135 | 17066 | C01089 |  | 300-85-6 | 0.000692073 |  |
| 716 | L-Asparagine | S0104 | 169.0062689 | 118.576 | 132.05349 | C4H8N2O3 | HMDB00168 | 6267 | 17196 | C00152 | 14 | 70-47-3 | 0.019061952 | Lipids and derivatives |
| 1181 | Pyridoxine | S0080 | 170.0796389 | 132.6095 | 169.07389 | C8H11NO3 | HMDB00239 | 1054 | 16709 | C00314 | 5245 | 65-23-6 | 0.008437858 |  |
| 1240 | Desoxypeganine |  | 172.0956321 | 47.211 |  |  |  |  |  |  |  |  | 0.001815732 |  |
| 1110 | Xylitol | S0322 | 173.045539 | 97.764 | 152.06848 | C5H12O5 | HMDB02917 | 6912 | 1305691 | C00379 |  | 87-99-0 | 0.018783538 | Carbohydrates and derivatives |
| 1142 | Fosfomycin |  | 174.9546722 | 575.539 | 138.06 | C3H7O4P |  |  |  |  |  |  | 0.028209375 |  |
| 1321 | L-Arginine | S0062 | 175.1179404 | 521.209 | 174.11168 | C6H14N4O2 | HMDB00517 | 6322 | 16467 | C00062 | 5502 | 74-79-3 | 0.040150104 |  |
| 1334 | Hippuric acid, methyl ester |  | 176.069372 | 39.2545 | 193.2 | C10H11NO3 |  |  |  |  |  |  | 0.003950283 | Amino acids and derivatives |
| 1357 | Ornithine | S0208 | 177.0580337 | 27.378 | 132.08988 | C5H12N2O2 | HMDB00214 | 6262 | 15729 | C01602 | 27 | 70-26-8 (3184-13-2) | 0.000305691 | Amino acids and derivatives |
| 1224 | myo-Inositol | S0024 | 179.056535 | 383.2135 | 180.06339 | C6H12O6 | HMDB00211 |  | 17268 | C00137 | 5221 | 87-89-8 | 0.018169096 | Lipids and derivatives |
| 1262 | L-Iditol | L0396 | 181.0722074 | 291.6155 | 182.07904 | C6H14O6 | HMDB11632 | 5460044 | 18202 | C01507 |  | 488-45-9 | 0.004638629 | Carbohydrates and derivatives |
| 859 | D-Threitol | S0351 | 181.072213 | 291.954 | 122.05791 | C4H10O4 | HMDB04136 | 169019 | 48300 | C16884 |  | 2418-52-2 | 0.004638629 |  |
| 873 | 4-Pyridoxic acid | L0261 | 182.04621 | 36.645 | 183.05316 | C8H9NO4 | HMDB00017 | 6723 | 17405 | C00847 | 239 | 82-82-6 | 0.004360613 |  |
| 1421 | L-Tyrosine | S0064 | 182.0797499 | 295.935 | 181.07389 | C9H11NO3 | HMDB00158 | 6057 | 17895 | C00082 | 34 | 60-18-4 | 0.001035467 | Lipids and derivatives |
| 1449 | Phosphorylcholine | L0132 | 184.0723486 | 497.674 | 183.06605 | C5H14NO4P | HMDB01565 | 1014 | 18132 | C00588 | 6326 | 107-73-3 (72556-74-2) | 0.006650218 | Amino acids and derivatives |
| 996 | 2-Methyl-3-hydroxybutyric acid | L0197 | 185.0164231 | 235.656 | 118.063 | C5H10O3 | HMDB00354 | 160471 | 37051 |  | 3786 | 473-86-9 | 0.001526645 |  |
| 1351 | Azelaic acid | S0199 | 187.0979887 | 323.439 | 188.10486 | C9H16O4 | HMDB00784 | 2266 | 48131 | C08261 | 5750 | 123-99-9 | 0.002040851 |  |
| 1058 | N-Acetylglutamine | S0265 | 189.0858753 | 296.571 | 188.07971 | C7H12N2O4 | HMDB06029 | 25561 |  |  |  | 2490-97-3 | 0.000126685 |  |
| 1060 | N6-Acetyl-L-lysine | L0011 | 189.1218846 | 342.508 | 188.11609 | C8H16N2O3 | HMDB00206 | 92832 | 17752 | C02727 | 5216 | 692-04-6 | 0.000226808 | Amino acids and derivatives |
| 975 | Citrate | S0027 | 191.0199926 | 509.223 | 192.02701 | C6H8O7 | HMDB00094 | 311 | 30769 | C00158 | 124 | 77-92-9 | 0.030385605 | Carbohydrates and derivatives |
| 1399 | Isocitrate | S0115 | 191.0199981 | 505.1925 | 192.02701 | C6H8O7 | HMDB00193 | 1198 | 30887 | C00311 | 3328 | 320-77-4 (20226-99-7) | 0.030385605 |  |
| 977 | Quinate | L0443 | 191.0566523 | 346.251 | 192.06339 | C7H12O6 | HMDB03072 |  | 17521 | C00296 | 3329 | 77-95-2 | 0.001155427 | Amino acids and derivatives |
| 1561 | Quinaldic acid | n00570 | 191.0799908 | 41.5685 | 173.04768 | C10H7NO2 | HMDB00842 | 7124 | 18386 | C06325 | 5805 | 34249 | 0.002607168 |  |
| 1121 | Vanillin | L0436 | 194.0798742 | 186.9315 | 152.04735 | C8H8O3 | HMDB12308 | 1183 | 18346 | C00755 |  | 121-33-5 | 0.000329001 |  |
| 1596 | Phenylacetylglycine | S0200 | 194.0801274 | 183.767 | 193.07389 | C10H11NO3 | HMDB00821 | 68144 | 27480 | C05598 | 4237 | 500-98-1 | 0.000330622 |  |
| 1125 | Agmatine | S0191 | 194.1374587 | 270.9145 | 130.12185 | C5H14N4 | HMDB01432 | 199 | 17431 | C00179 | 3523 | 306-60-5 (2482-00-0) | 0.000886943 | Amino acids and derivatives |
| 1639 | Pro-Val |  | 197.1273639 | 49.1335 | 214.26 | C10H18N2O3 |  |  |  |  |  |  | 0.000841546 |  |
| 1511 | O-Phospho-L-threonine | L0209 | 198.0147191 | 87.527 | 199.02458 | C4H10NO6P | HMDB11185 | 3246323 | 37525 | C12147 |  | 1114-81-4 | 0.060380395 | Amino acids and derivatives |
| 1529 | L-homocysteic acid | S0114 | 199.0437902 | 32.485 | 183.02015 | C4H9NO5S | HMDB02205 | 177491 | 488363 | C16511 | 6545 | 14857-77-3 | 0.004648231 | Amino acids and derivatives |
| 1675 | p-CHLOROPHENYLALANINE |  | 200.0460277 | 153.886 | 199.63 | C9H10ClNO2 |  |  |  |  |  |  | 0.006010343 | Amino acids and derivatives |
| 1207 | 9-Decen-1-ol |  | 201.1260993 | 35.338 | 156.27 | C10H20O |  |  |  |  |  |  | 0.000508224 | Lipids and derivatives |
| 1720 | 3-Hydroxybenzoate | S0388 | 202.0441932 | 234.856 | 138.0317 | C7H6O3 | HMDB02466 | 7420 | 30764 | C00587 | 6690 | 36320 | 0.289828138 |  |
| 1626 | Pantothenol | S0314 | 204.1246345 | 75.016 | 205.13141 | C9H19NO4 | HMDB04231 | 4678 | 895821 | C05944 | 7032 | 81-13-0 | 0.008672618 |  |
| 1658 | Malonic acid | S0204 | 207.0130993 | 100.97 | 104.01096 | C3H4O4 | HMDB00691 | 867 | 30794 | C00383 | 3237 | 141-82-2 | 0.006992297 |  |
| 1662 | D-Arabinono-1,4-lactone |  | 207.051396 | 79.4435 | 148.114 | C5H8O5 |  |  |  |  |  |  | 0.003869385 | Carbohydrates and derivatives |
| 1844 | Monomethyl glutaric acid | S0247 | 210.0780619 | 71.166 | 146.05791 | C6H10O4 | HMDB00858 | 73917 | 1007322 |  | 5821 | 1501-27-5 | 0.000587368 | Lipids and derivatives |
| 1852 | Sinapyl alcohol | L0398 | 211.0970336 | 65.8875 | 210.08921 | C11H14O4 | HMDB13070 | 5280507 | 64557 | C02325 |  | 537-33-7 | 0.009498359 |  |
| 1297 | sn-Glycerol 3-phosphoethanolamine | S0362 | 214.0496384 | 383.698 | 215.05588 | C5H14NO6P | HMDB59660 | 5459861 | 16929 |  |  |  | 0.000899802 | Lipids and derivatives |
| 1901 | Lys-Cys |  | 214.1060216 | 355.6195 |  |  |  |  |  |  |  |  | 0.001689466 | Amino acids and derivatives |
| 1385 | N-.alpha.-Acetyl-L-arginine |  | 217.1278121 | 360.682 | 216.24 | C8H16N4O3 |  |  |  |  |  |  | 0.000396458 |  |
| 1952 | Dodecanoic acid | S0184 | 218.2102258 | 97.163 | 200.17763 | C12H24O2 | HMDB00638 | 3893 | 30805 | C02679 | 5611 | 143-07-7 | 0.00407574 | Lipids and derivatives |
| 2015 | Acetylcholine | L0265 | 222.0279607 | 235.5075 | 145.11028 | C7H15NO2 | HMDB00895 | 187 | 15355 | C01996 | 57 | 60-31-1 | 0.002824691 | Lipids and derivatives |
| 2026 | Glycerol | S0302 | 223.0626897 | 33.678 | 92.04735 | C3H8O3 | HMDB00131 | 753 | 17522 | C00116 | 105 | 56-81-5 | 0.005524313 | Amino acids and derivatives |
| 1942 | Myristoleic acid | n00940 | 225.1868538 | 47.223 | 226.19328 | C14H26O2 | HMDB02000 | 5281119 | 27781 | C08322 | 6424 | 544-64-9 | 0.009400833 | Lipids and derivatives |
| 1959 | 3-Indolepropionic acid | n00704 | 226.0282946 | 153.4195 | 189.07898 | C11H11NO2 | HMDB02302 |  |  |  |  | 830-96-6 | 0.010880528 |  |
| 2146 | (R)-mevalonic acid 5-Phosphate | L0453 | 229.1410959 | 37.166 | 228.137 | C6H13O7P | HMDB01343 | 439400 | 17436 | C01107 | 6177 |  | 0.001278507 |  |
| 1670 | D-Mannose | S0138 | 239.0781884 | 383.01 | 180.06339 | C6H12O6 | HMDB00169 | 18950 | 4208 | C00159 |  | 3458-28-4 | 0.003804086 | Carbohydrates and derivatives |
| 2188 | D-Fructose | S0236 | 239.0782091 | 382.9005 | 180.06339 | C6H12O6 | HMDB00660 | 439709 | 28645 | C00095 | 135 | 57-48-7 | 0.003804086 | Carbohydrates and derivatives |
| 2310 | 4-Methoxycinnamic acid | S0234 | 239.0943569 | 27.979 | 178.063 | C10H10O3 | HMDB02040 | 699414 | 260249 |  | 6453 | 830-09-1 | 0.001101071 | Amino acids and derivatives |
| 2204 | O-Succinyl-L-homoserine | L0319 | 240.0449109 | 157.6145 | 219.07429 | C8H13NO6 |  |  |  | C01118 |  | 1492-23-5 | 0.215256056 | Amino acids and derivatives |
| 2243 | Thiopental |  | 241.1027074 | 34.902 | 242.338 | C11H18N2O2S |  |  |  |  |  |  | 0.019469801 |  |
| 2251 | Pentadecanoic Acid | S0364 | 241.2182 | 48.015 | 242.22458 | C15H30O2 | HMDB00826 | 13849 |  | C16537 | 5789 | 1002-84-2 | 0.076357347 | Lipids and derivatives |
| 2269 | N-Acetyl-D-galactosamine | L0459 | 242.1772894 | 122.2185 | 221.2078 | C8H15NO6 | HMDB00212 | 14035695 | 17994 | C01132 |  |  | 0.017989583 | Carbohydrates and derivatives |
| 2421 | Lumichrome | S0293 | 243.0863975 | 63.0025 | 242.08038 | C12H10N4O2 |  |  |  | C01727 |  | 1086-80-2 | 0.000866481 |  |
| 1760 | Arg-Ser |  | 244.1396341 | 402.013 |  |  |  |  |  |  |  |  | 0.000330606 | Amino acids and derivatives |
| 2469 | D-Biotin | L0014 | 245.0944427 | 247.111 | 244.08817 | C10H16N2O3S | HMDB00030 | 171548 | 15956 | C00120 | 243 | 58-85-5 | 0.000503297 |  |
| 1802 | Arg-Ala |  | 246.155049 | 412.689 | 305.33 | C11H23N5O5 |  |  |  |  |  |  | 0.000330606 | Amino acids and derivatives |
| 1805 | Lys-Val |  | 246.1800724 | 546.737 | 245.32 | C11H23N3O3 |  |  |  |  |  |  | 0.000226386 | Amino acids and derivatives |
| 2496 | Myristic acid | S0207 | 246.2420319 | 87.955 | 228.20893 | C14H28O2 | HMDB00806 | 11005 | 28875 | C06424 | 196 | 544-63-8 | 0.006450887 | Lipids and derivatives |
| 2330 | Sedoheptulose |  | 247.017673 | 375.6955 | 210.18186 | C7H14O7 |  |  |  |  |  |  | 0.004976358 | Carbohydrates and derivatives |
| 1827 | Pro-Met |  | 247.1145493 | 84.583 | 382.47 | C18H26N2O5S |  |  |  |  |  |  | 0.000367168 | Lipids and derivatives |
| 1902 | D-Ribulose 5-phosphate | L0289 | 253.0134591 | 366.768 | 230.01916 | C5H11O8P | HMDB00618/HMDB01548 | 53477706 | 17797 | C00199 | 5591 | 4151-19-3 (4300-28-1) | 0.000433619 | Carbohydrates and derivatives |
| 2401 | cis-9-Palmitoleic acid | L0166 | 253.2180199 | 4.971 | 254.22458 | C16H30O2 | HMDB03229 | 445638 | 28716 | C08362 | 188 | 373-49-9 | 0.004927448 | Lipids and derivatives |
| 2693 | Glycerophosphocholine | S0106 | 258.1096488 | 379.5545 | 258.11065 | C8H21NO6P+ | HMDB00086 | 71920 | 16870 | C00670 | 370 | 28319-77-9 | 0.002706196 |  |
| 2491 | D-Erythrose 4-phosphate | L0290 | 259.0236103 | 441.0255 | 200.00859 | C4H9O7P | HMDB01321 | 122357 | 48153 | C00279 | 6158 | 585-18-2 (103302-15-4) | 0.011640063 | Carbohydrates and derivatives |
| 2024 | alpha-D-Glucose 1-phosphate | L0283 | 261.0361036 | 441.73 | 260.02972 | C6H13O9P | HMDB01586 | 439165 | 16077 | C00103 | 6331 | 56401-20-8(59-56-3) | 0.011640063 |  |
| 2808 | Thiamine | S0085 | 265.110637 | 366.584 | 265.11231 | C12H17N4OS | HMDB00235 | 1130 | 18385 | C00378 | 5242 | 59-43-8 (67-03-8) | 0.002672275 | Nucleic acids and derivatives |
| 2602 | Adenosine | L0016 | 266.0913736 | 167.655 | 267.09675 | C10H13N5O4 | HMDB00050 | 60961 | 16335 | C00212 | 86 | 58-61-7 | 0.001924435 | Lipids and derivatives |
| 2614 | Inosine | S0032 | 267.0743108 | 212.044 | 268.08077 | C10H12N4O5 | HMDB00195 | 6021 | 17596 | C00294 | 84 | 58-63-9 | 0.007221867 | Nucleic acids and derivatives |
| 2625 | Hexadecanedioic acid |  | 267.1974797 | 64.8545 | 286.407 | C16H30O4 |  |  |  |  |  |  | 0.015944345 | Lipids and derivatives |
| 2687 | 16-Hydroxypalmitic acid | S0320 | 271.2286613 | 39.17 | 272.23514 | C16H32O3 | HMDB06294 | 7058075 | 55329 | C18218 |  | 506-13-8 | 0.004382819 | Lipids and derivatives |
| 2963 | Palmitic acid | S0135 | 274.2741744 | 81.5525 | 256.24023 | C16H32O2 | HMDB00220 | 985 | 15756 | C00249 | 187 | 21096 | 0.722796889 | Amino acids and derivatives |
| 3014 | L-Saccharopine | L0155 | 277.1386086 | 430.292 | 276.13214 | C11H20N2O6 | HMDB00279 | 160556 | 16927 | C00449 | 383 | ?997-68-2 | 0.000289385 | Nucleic acids and derivatives |
| 3030 | Pro-Tyr |  | 278.1223656 | 488.591 | 278.3 | C14H18N2O4 |  |  |  |  |  |  | 0.003026624 | Amino acids and derivatives |
| 2790 | Thymidine | S0030 | 279.0402185 | 158.421 | 242.09027 | C10H14N2O5 | HMDB00273 | 5789 | 17748 | C00214 | 3375 | 50-89-5 | 0.009053904 | Nucleic acids and derivatives |
| 2797 | Linoleic acid | L0126 | 279.2343039 | 38.733 | 280.24023 | C18H32O2 | HMDB00673 | 5280450 | 17351 | C01595 | 191 | 60-33-3 | 0.121300811 | Lipids and derivatives |
| 2827 | Oleic acid | L0123 | 281.2498137 | 38.4415 | 282.25588 | C18H34O2 | HMDB00207 | 445639 | 16196 | C00712 | 190 | 112-80-1 | 0.004927448 | Lipids and derivatives |
| 3120 | 2'-O-methyladenosine | L0234 | 282.1186567 | 103.938 | 281.11241 | C11H15N5O4 | HMDB04326 | 317398 | 119928 |  |  | 2140-79-6 | 0.001264594 |  |
| 3126 | Butoxyacetic acid | n00265 | 282.1901235 | 166.492 | 132.07864 | C6H12O3 | HMDB41844 | 41958 |  |  |  | 2516-93-0 | 0.000343981 |  |
| 3125 | Ethyl 3-hydroxybutyrate |  | 282.1902484 | 262.7615 | 132.16 | C6H12O3 |  |  |  |  |  |  | 0.001011006 | Nucleic acids and derivatives |
| 2867 | Stearic acid | S0233 | 283.2669205 | 40.013 | 284.27153 | C18H36O2 | HMDB00827 | 5281 | 28842 | C01530 | 189 | 21128 | 2.179211417 | Carbohydrates and derivatives |
| 2871 | Pyridoxal 5'-phosphate | L0176 | 283.977591 | 54.456 | 247.02458 | C8H10NO6P | HMDB01491 | 1051 | 18405 | C00018 | 6275 | 54-47-7 | 0.00517231 |  |
| 2924 | 16b-Hydroxyestradiol |  | 287.1701062 | 34.7275 | 288.381 | C18H24O3 |  |  |  |  |  |  | 0.004011317 | Lipids and derivatives |
| 3230 | Heptadecanoic acid | S0367 | 288.2887471 | 78.6325 | 270.25588 | C17H34O2 | HMDB02259 | 10465 | 32365 |  | 6578 | 506-12-7 | 0.000589028 | Lipids and derivatives |
| 2952 | 3'-Hydroxyropivacaine |  | 289.1856228 | 33.105 | 290.40054 | C17H26N2O2 |  |  |  |  |  |  | 0.011826586 |  |
| 3023 | L-Arabinono-1,4-lactone | S0397 | 295.227793 | 37.352 | 148.114 | C5H8O5 |  |  |  | C01114 |  | 51532-86-6 | 0.013246749 |  |
| 3037 | S-Methyl-5'-thioadenosine | L0036 | 296.0837023 | 101.5605 | 297.08956 | C11H15N5O3S | HMDB01173 | 439176 | 17509 | C00170 | 3425 | 2457-80-9 | 0.003054796 | Lipids and derivatives |
| 3062 | Nname,cis-9,10-Epoxystearic acid |  | 297.2447003 | 37.8115 | 298.46076 | C18H34O3 |  |  |  |  |  |  | 0.017778615 | Lipids and derivatives |
| 3441 | (Z)-6-Octadecenoic acid |  | 300.2893904 | 170.9725 | 282.46 | C18H34O2 |  |  |  |  |  |  | 0.006043187 |  |
| 3483 | Sphinganine | L0159 | 302.3047263 | 138.104 | 301.29808 | C18H39NO2 | HMDB00269 | 91486 | 16566 | C00836 | 5268 | 764-22-7 | 0.001858104 | Amino acids and derivatives |
| 3174 | Arachidonic Acid (peroxide free) | L0145 | 303.2345755 | 38.9335 | 304.24023 | C20H32O2 | HMDB01043 | 444899 | 15843 | C00219 | 193 | 506-32-1 | 0.019762376 | Lipids and derivatives |
| 3529 | Felbamate |  | 305.0415357 | 34.57 | 238.24 | C11H14N2O4 |  |  |  |  |  |  | 0.001700918 |  |
| 3592 | Geranylgeraniol |  | 308.2943557 | 34.6185 | 290.48 | C20H34O |  |  |  |  |  |  | 0.00255226 |  |
| 3267 | (4Z,7Z,10Z,13Z,16Z,19Z)-4,7,10,13,1 6,19-Docosahexaenoic acid | L0154 | 309.2199887 | 31.773 | 328.24023 | C22H32O2 | HMDB02183 | 445580 | 28125 | C06429 | 3457 | 6217-54-5 | 0.009004026 | Lipids and derivatives |
| 3442 | 20-HETE |  | 319.2351881 | 30.4785 | 320.47 | C20H32O3 |  |  |  |  |  |  | 0.978895847 | Lipids and derivatives |
| 3794 | Sibutramine |  | 321.2052585 | 35.974 | 279.85 | C17H26ClN |  |  |  |  |  |  | 0.000622491 |  |
| 3799 | Erucic acid | S0288 | 321.3145643 | 33.8635 | 338.31848 | C22H42O2 | HMDB02068 | 5281116 | 28792 | C08316 | 6470 | 112-86-7 | 0.011734348 | Lipids and derivatives |
| 3868 | Glycerol 1-myristate |  | 325.2368779 | 38.865 | 302.45 | C17H34O4 |  |  |  |  |  |  | 0.002489407 | Lipids and derivatives |
| 3953 | Eicosapentaenoic Acid ethyl ester |  | 330.2630345 | 38.5295 | 330.5 | C22H34O2 |  |  |  |  |  |  | 0.001748825 | Lipids and derivatives |
| 3071 | beta-Nicotinamide D-ribonucleotide | L0168 | 335.063009 | 478.6295 | 335.06443 | C11H16N2O8P+ | HMDB59645 | 14181 | 14648 | C00455 |  | 1094-61-7 | 0.000293196 |  |
| 3656 | Erucamide | L0202 | 336.3283486 | 34.0265 | 337.33446 | C22H43NO |  |  |  |  |  | 112-84-5 | 0.057454853 | Amino acids and derivatives |
| 4070 | (+)-Methamphetamine |  | 337.199892 | 34.4595 | 149.23 | C10H15N |  |  |  |  |  |  | 0.005766047 |  |
| 4071 | (2E,6E)-Farnesol | S0396 | 337.2347867 | 37.4395 | 222.3663 | C15H26O | HMDB04305 | 445070 | 16619 | C01126 | 7048 | 4602-84-0 | 0.004530637 | Lipids and derivatives |
| 3751 | Sucrose | S0033 | 341.1109254 | 389.023 | 342.11622 | C12H22O11 | HMDB00258 | 5988 | 17992 | C00089 | 137 | 57-50-1 | 0.004104316 | Carbohydrates and derivatives |
| 3222 | 3',5'-Cyclic guanosine monophosphate | L0381 | 346.0538924 | 307.904 | 345.04744 | C10H12N5O7P | HMDB01314 |  |  | C00942 |  | 7665-99-8 | 0.000246438 | Amino acids and derivatives |
| 4638 | Adenosine monophosphate (AMP) |  | 346.0574652 | 421.8835 | 347.22 | C10H14N5O7P |  |  |  |  |  |  | 0.001924435 | Nucleic acids and derivatives |
| 4241 | Adenosine 3'-monophosphate | L0392 | 348.0693163 | 422.199 | 347.06309 | C10H14N5O7P | HMDB03540 | 41211 | 28931 | C01367 |  | 84-21-9 | 0.001924435 |  |
| 3837 | Tetrahydrocorticosterone |  | 349.2448624 | 24.932 | 350.49 | C21H34O4 |  |  |  |  |  |  | 0.005147384 | Lipids and derivatives |
| 3849 | Docosatrienoic Acid |  | 350.3085362 | 34.4785 | 334.54 | C22H38O2 |  |  |  |  |  |  | 0.009298993 | Lipids and derivatives |
| 3866 | 20-hydroxy LTB4 |  | 351.2230503 | 104.496 |  |  |  |  |  |  |  |  | 0.005101207 | Lipids and derivatives |
| 4375 | Phytanic acid | L0401 | 354.3359871 | 34.2985 | 312.30283 | C20H40O2 | HMDB00801 | 26840 | 16285 | C01607 | 5765 | 14721-66-5 | 0.004497547 | Carbohydrates and derivatives |
| 4388 | Pentoxifylline |  | 355.0638885 | 55.902 | 278.31 | C13H18N4O3 |  |  |  |  |  |  | 0.025204218 | Nucleic acids and derivatives |
| 3962 | Glucosamine | S0082 | 357.1425826 | 159.915 | 179.07937 | C6H13NO5 | HMDB01514 | 439213 | 5417 | C00329 | 266 | 3416-24-8 (66-84-2/14257-69-3) | 0.007992912 | Carbohydrates and derivatives |
| 4479 | Behenic acid | S0350 | 358.3676215 | 71.906 | 340.33413 | C22H44O2 | HMDB00944 | 8215 | 28941 | C08281 | 260 | 112-85-6 | 0.008789276 | Lipids and derivatives |
| 3439 | Isomaltose | S0341 | 360.1488022 | 388.944 | 342.11622 | C12H22O11 | HMDB02923 | 439193 | 28189 | C00252 | 412 | 499-40-1 | 0.000292942 | Carbohydrates and derivatives |
| 4514 | D-(+)-Melibiose | S0303 | 360.1489411 | 389.097 | 342.11622 | C12H22O11 | HMDB00048 | 440658 | 28053 | C05402 | 3478 | 585-99-9 | 0.000292942 | Carbohydrates and derivatives |
| 4084 | Tetracosanoic acid | L0302 | 367.3595224 | 37.903 | 368.36543 | C24H48O2 | HMDB02003 | 11197 | 28866 | C08320 | 6427 | 557-59-5 | 0.006109829 | Lipids and derivatives |
| 4400 | 5(S)-HpETE |  | 395.2460122 | 39.275 | 336.47 | C20H32O4 |  |  |  |  |  |  | 0.01100657 | Lipids and derivatives |
| 3605 | Hexacosanoic acid | L0300 | 395.3918764 | 37.8355 | 396.39673 | C26H52O2 | HMDB02356 | 10469 | 31009 |  | 6642 | 506-46-7 | 0.003546313 |  |
| 3673 | Trehalose | S0313 | 401.1324665 | 389.142 | 342.11622 | C12H22O11 | HMDB00975 | 7427 | 16551 | C01083 | 5913 | 99-20-7 | 0.003525394 |  |
| 3711 | Acetylcarnitine | L0133 | 405.2283703 | 73.565 | 203.11576 | C9H17NO4 | HMDB00201 | 1 |  | C02571 | 5213 | 870-77-9 (2504-11-2) | 0.003054796 | Nucleic acids and derivatives |
| 5735 | Tangeritin |  | 439.0848885 | 233.591 | 372.37 | C20H20O7 |  |  |  |  |  |  | 0.006368182 |  |
| 6485 | Quercitrin | n02460 | 487.0555583 | 35.9785 | 448.10056 | C21H20O11 | HMDB33751 | 5280459 | 17558 | C01750 |  | 522-12-3 | 0.002031158 |  |
| 5154 | 1-Palmitoyl-sn-glycero-3-phosphocholine |  | 496.3387518 | 188.515 | 495.63 | C24H50NO7P |  |  |  |  |  |  | 0.000162226 |  |
| 6407 | UDP-N-acetylglucosamine | L0303 | 608.0889356 | 406.94 | 607.08158 | C17H27N3O17P2 | HMDB00290 | 445675 | 16264 | C00043 | 5281 | 528-04-1 (91183-98-1) | 0.00012116 | Nucleic acids and derivatives |
| 5018 | Glutathione disulfide |  | 611.1496528 | 479.884 | 612.63 | C20H32N6O12S2 |  |  |  |  |  |  | 0.003640736 | Nucleic acids and derivatives |
| 6981 | Nicotinamide adenine dinucleotide (NAD) | L0009 | 664.1167496 | 419.778 | 663.10913 | C21H27N7O14P2 | HMDB00902 | 5893 | 15846 | C00003 | 5858 | 53-84-9 | 0.000133365 | Lipids and derivatives |
| 9873 | PC(16:0/16:0) | S0151 | 756.5550665 | 143.1235 | 733.56216 | C40H80NO8P | HMDB00564 | 452110 |  | C00157 | 5548 | 63-89-8 | 0.000298247 |  |
| 10135 | Thioetheramide-PC |  | 780.5536016 | 37.45 | 789.27 | C45H93N2O4PS |  |  |  |  |  |  | 0.000228166 |  |
| 8067 | 1,2-dioleoyl-sn-glycero-3-phosphatidylcholine |  | 786.6016368 | 141.452 | 786.11 | C44H84NO8P |  |  |  |  |  |  | 0.000455275 | Lipids and derivatives |
| 8241 | 1-Stearoyl-2-oleoyl-sn-glycerol 3-phosphocholine (SOPC) | L0411 | 810.5980963 | 37.498 | 787.60911 | C44H86NO8P | HMDB08038 |  |  |  |  |  | 0.000179333 | Lipids and derivatives |

Note: SZ, VU-CER method;

ST, VU method;

id, The unique data number of this substance in this qualitative analysis;

MS2 name, Material name obtained by qualitative analysis of secondary mass spectrometry;

labid, The number of the substance in the local database;

m/z, Specific charge of the substance;

rt, Retention time of the substance;

Exact Mass, The exact molecular weight of the substance;

Formula, The molecular formula corresponding to the substance;

HMDB, The index of the substance in the HMDB database;

PubChem, The number of this substance in the PubChem database;

ChEBI, The index of this substance in the ChEBI database;

KEGG COMPOUND ID, Index of this substance in the KEGG COMPOUND database;

METLIN, The index of the substance in the METLIN database;

CAS, CAS number of the substance;

mean, Peak area of the substance;

**Table S3.** Differentially expressed metabolites using CER-VU and VU extraction methods.

| MS2 name | Trend | VIP | P-value | Fold change | Category |
| --- | --- | --- | --- | --- | --- |
| p-Chlorophenylalanine | 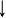 | 1.20 | 9.90×10^-3^ | 0.52 | Amino acids and derivatives |
| L-Phenylalanine | 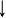 | 1.20 | 9.90×10^-3^ | 0.52 | Amino acids and derivatives |
| L-Glutamine | 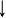 | 1.21 | 7.61×10^-4^ | 0.60 | Amino acids and derivatives |
| L-Tyrosine | 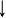 | 1.03 | 1.06×10^-2^ | 0.79 | Amino acids and derivatives |
| L-Pyroglutamic acid | 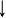 | 1.38 | 4.23×10^-4^ | 0.39 | Amino acids and derivatives |
| Creatine | 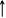 | 1.06 | 8.06×10^-3^ | 1.27 | Amino acids and derivatives |
| L-Citrulline | 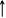 | 1.28 | 2.10×10^-4^ | 2.01 | Amino acids and derivatives |
| L-Arginine | 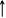 | 1.13 | 1.44×10^-2^ | 2.61 | Amino acids and derivatives |
| L-Saccharopine | 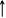 | 1.22 | 8.31×10^-4^ | 2.28 | Amino acids and derivatives |
| L-Proline | 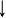 | 1.01 | 3.07×10^-2^ | 2.19 | Amino acids and derivatives |
| Creatinine | 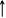 | 1.14 | 2.65E-03 | 1.38 | Amino acids and derivatives |
| D-Proline | 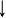 | 1.09 | 7.44E-03 | 0.64 | Amino acids and derivatives |
| Tyramine | 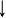 | 1.31 | 7.70E-03 | 0.25 | Amino acids and derivatives |
| N-Acetylglutamine | 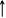 | 1.23 | 8.16E-03 | 2.06 | Amino acids and derivatives |
| N-acetyl-L-lysine | 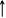 | 1.31 | 2.61E-03 | 2.74 | Amino acids and derivatives |
| N-acetyl-L-arginine | 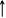 | 1.29 | 2.99E-03 | 2.44 | Amino acids and derivatives |
| Pro-Val | 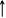 | 1.08 | 8.30E-03 | 1.96 | Amino acids and derivatives |
| Lys-Cys | 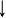 | 1.04 | 1.08E-02 | 0.77 | Amino acids and derivatives |
| Arg-Ser | 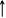 | 1.34 | 2.39E-03 | 2.54 | Amino acids and derivatives |
| Arg-Ala | 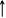 | 1.10 | 6.31E-03 | 1.47 | Amino acids and derivatives |
| Lys-Val | 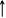 | 1.44 | 2.67E-05 | 3.14 | Amino acids and derivatives |
| Pro-Met | 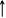 | 1.27 | 1.10E-02 | 4.26 | Amino acids and derivatives |
|  |  |  |  |  |  |
| phosphorylcholine | 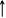 | 1.29 | 2.56×10^-4^ | 2.14 | Lipids and derivatives |
| acetylcholine | 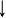 | 1.24 | 3.42×10^-4^ | 0.83 | Lipids and derivatives |
| palmitic acid | 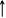 | 1.00 | 1.01×10^-2^ | 1.81 | Lipids and derivatives |
| behenic acid | 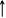 | 1.30 | 1.03×10^-2^ | 21.98 | Lipids and derivatives |
| (Z)-6-Octadecenoic acid | 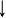 | 1.30 | 1.14×10^-2^ | 0.02 | Lipids and derivatives |
| PC(16:0/16:0) | 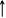 | 1.22 | 1.77×10^-3^ | 3.28 | Lipids and derivatives |
| myo-Inositol | 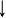 | 1.11 | 3.16×10^-2^ | 0.24 | Lipids and derivatives |
| sn-Glycerol 3-phosphoethanolamine | 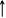 | 1.16 | 3.75×10^-4^ | 2.41 | Lipids and derivatives |
| Hexacosanoic acid | 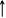 | 1.02 | 1.11×10^-2^ | 1.85 | Lipids and derivatives |
|  |  |  |  |  |  |
| D-Mannose | 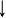 | 1.24 | 1.89×10^-5^ | 0.65 | Carbohydrates and derivatives |
| alpha-D-Glucose 1-phosphate | 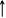 | 1.28 | 7.76×10^-5^ | 4.08 | Carbohydrates and derivatives |
| trehalose | 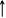 | 1.29 | 2.82×10^-5^ | 3.04 | Carbohydrates and derivatives |
| isomaltose | 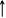 | 1.29 | 2.56×10^-4^ | 2.49 | Carbohydrates and derivatives |
| UDP-N-acetylglucosamine | 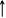 | 1.26 | 4.26×10^-3^ | 2.63 | Carbohydrates and derivatives |
|  |  |  |  |  |  |
| Hippuric acid methyl ester | 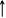 | 1.29 | 2.96×10^-3^ | 4.76 |  |
| 2-Oxoadipic acid | 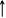 | 1.37 | 3.28×10^-5^ | 7.60 |  |
| 1,2-Benzenedicarboxylic acid | 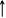 | 1.38 | 2.19×10^-4^ | 9.51 |  |
| Pyridoxine | 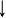 | 1.25 | 2.10×10^-4^ | 0.53 |  |
| L-Carnitine | 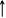 | 1.03 | 8.06×10^-3^ | 1.30 |  |
| Picolinic acid | 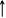 | 1.18 | 1.85×10^-3^ | 2.40 |  |
| Succinate | 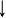 | 1.16 | 4.36×10^-3^ | 0.59 |  |
| Benzoic acid | 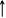 | 1.37 | 2.21×10^-4^ | 6.93 |  |
| Citrate | 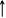 | 1.14 | 1.00×10^-3^ | 2.06 |  |
| Acetylcarnitine | 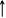 | 1.03 | 3.50×10^-2^ | 1.82 |  |
| 1-Aminocyclopropanecarboxylic acid | 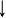 | 1.39 | 2.34×10^-4^ | 0.38 |  |
| DL-2,4-Diaminobutyric acid | 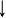 | 1.38 | 1.51×10^-5^ | 0.42 |  |
| 3-Furancarboxylic acid | 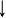 | 1.38 | 9.02×10^-4^ | 0.41 |  |
| Pyrrolidine | 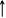 | 1.17 | 1.14×10^-2^ | 2.66 |  |
| (3-Carboxypropyl)trimethylammonium cation | 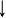 | 1.45 | 1.39×10^-5^ | 0.10 |  |
| Formylanthranilic acid | 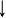 | 1.27 | 4.74×10^-4^ | 0.50 |  |
| Butyl lactate | 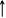 | 1.06 | 9.14×10^-3^ | 1.95 |  |
| Quinaldic acid | 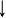 | 1.40 | 1.07×10^-3^ | 0.31 |  |
| Vanillin | 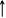 | 1.19 | 4.09×10^-3^ | 3.21 |  |
|  |  |  |  |  |  |
| Adenine | 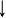 | 1.11 | 3.54×10^-3^ | 0.38 | Nucleic acids and derivatives |
| Hypoxanthine | 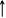 | 1.15 | 7.90×10^-3^ | 1.65 | Nucleic acids and derivatives |
| Thymidine | 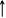 | 1.19 | 3.38×10^-4^ | 1.96 | Nucleic acids and derivatives |
| Adenosine 3'-monophosphate | 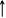 | 1.06 | 2.93×10^-3^ | 1.56 | Nucleic acids and derivatives |
| Nicotinamide | 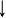 | 1.38 | 9.13×10^-4^ | 0.21 | Nucleic acids and derivatives |
| 3',5'-Cyclic guanosine monophosphate | 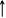 | 1.38 | 4.45×10^-3^ | 3.54 | Nucleic acids and derivatives |
| beta-Nicotinamide D-ribonucleotide | 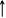 | 1.28 | 4.49×10^-4^ | 3.17 | Nucleic acids and derivatives |
| Nicotinamide adenine dinucleotide | 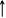 | 1.39 | 8.59×10^-4^ | 3.39 | Nucleic acids and derivatives |
|  |  |  |  |  |  |
